# Supplementary material for: Conserved phosphorylation hotspots in eukaryotic protein domain families
Source: Nat Commun. 2019 Apr 29;10:1977. doi: 10.1038/s41467-019-09952-x (PMC6488607; doi:10.1038/s41467-019-09952-x)
Supplement: Supplementary file 1 — Supplementary Information [file 41467_2019_9952_MOESM1_ESM.docx]

**Conserved phosphorylation hotspots in eukaryotic protein domain families**

Marta J. Strumillo^1^, Michaela Oplová^2,3^, Cristina Viéitez^1,4^, David Ochoa^1^, Mohammed Shahraz^4^, Bede P. Busby^1,4^, Richelle Sopko^5^, Romain A. Studer^1,6^, Norbert Perrimon^5,7,8^, Vikram G. Panse^2^, Pedro Beltrao^1*^

^1^European Molecular Biology Laboratory, European Bioinformatics Institute, Wellcome Genome Campus, Hinxton, CB10 1SD, Cambridge, UK

^2^Institute of Medical Microbiology, University of Zurich, CH-8006 Zurich, Switzerland;

^3^Institute of Biochemistry, ETH Zurich, CH-8093 Zurich, Switzerland

^4^European Molecular Biology Laboratory, Genome Biology Unit, 69117 Heidelberg, Germany

^5^Department of Genetics, Harvard Medical School, 77 Avenue Louis Pasteur, Boston, MA 02115

^6^Corrent address: BenevolentAI, London NW1 1LW, UK.

^7^Drosophila RNAi Screening Center, Harvard Medical School, 77 Avenue Louis Pasteur, Boston, MA 02115

^8^Howard Hughes Medical Institute, 77 Avenue Louis Pasteur, Boston, MA 02115

^*^Corresponding author: pbeltrao@ebi.ac.uk

**Supplementary Information**


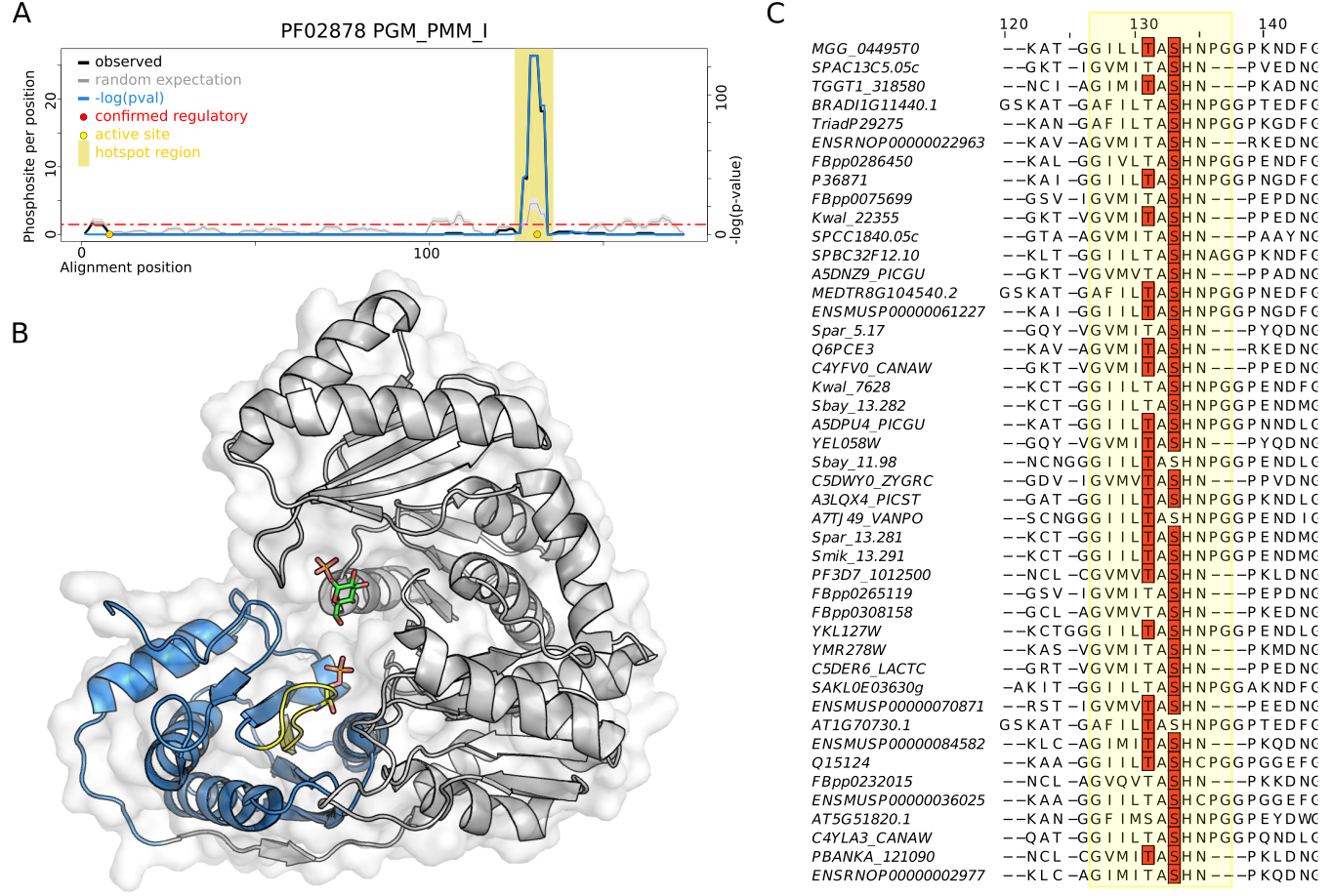


**Supplementary Figure 1 - Hotspot regions at a catalytic serine of the phosphoglucomutase/phosphomannomutase (PGM/PMM) domain (PF02878).** A) The hotspot region is represented in yellow segment and the catalytic serine residue within the hotspot region (yellow sticks) points is shown in the structure (B). This phospho-serine catalytic intermediary is capture in the MS phosphoproteomics data as shown in the alignment (C). There are often phosphorylated threonines next to the serine residue which could be miss-assigned phosphosites, or potentially these could be targeted by kinases.


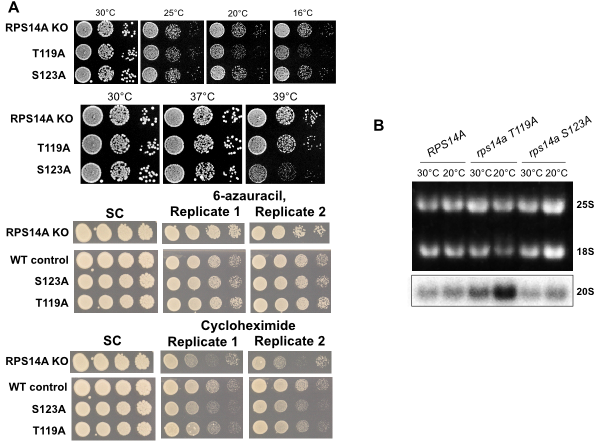


**Supplementary Figure 2 - Phenotypic assays for Rps14A phospho-deficient mutants and 20S pre-rRNA measurements.** A) Growth differences at different temperatures in optimal growth media and between the presence or absence of 6-azauracil and cycloheximide using spot dilution assay. B) Northern blot analyses measuring differences in 20S pre-rRNA levels for the mutant strains. Source data are provided as a Source Data file.


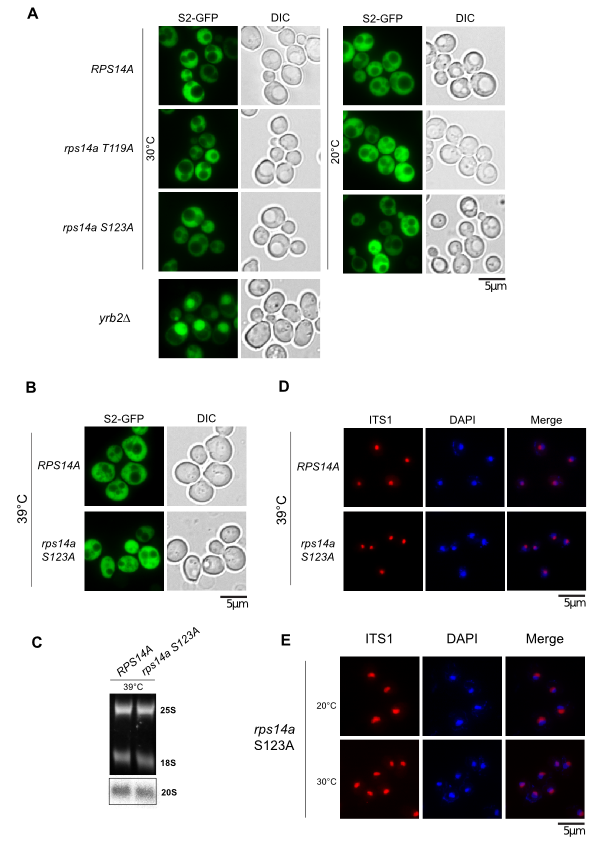


**Supplementary Figure 3 - Assays for Rps14A S123A mutant.** A and B) Early biogenesis defect of the small ribosomal subunit was tested by differences in localization of uS5-GFP for the WT control and the mutant strains at indicated temperatures. C) Northern blot analyses measuring differences in 20S pre-rRNA levels for the mutant strain. Source data are provided as a Source Data file. D and E) in situ hybridization with a Cy3-labeled oligonucleotide complementary to the 5′ sequence portion of ITS1 was assayed different temperatures.

**Supplementary Tables**

**Supplementary Table 1** - The ranked list of the top 50 commonly phosphorylated domains.

| **Pfam Id** | **Pfam domain name** | **Phos. count** | **Domain count** |
| --- | --- | --- | --- |
| PF00069.24 | Protein kinase domain | 2603 | 9063 |
| PF07714.16 | Protein tyrosine kinase | 677 | 2340 |
| PF00076.21 | RNA recognition motif. (a.k.a. RRM, RBD, or RNP domain) | 677 | 6970 |
| PF00443.28 | Ubiquitin carboxyl-terminal hydrolase | 661 | 885 |
| PF00012.19 | Hsp70 protein | 562 | 594 |
| PF07679.15 | Immunoglobulin I-set domain | 544 | 6101 |
| PF01576.18 | Myosin tail | 489 | 157 |
| PF00022.18 | Actin | 469 | 610 |
| PF00063.20 | Myosin head (motor domain) | 459 | 499 |
| PF00041.20 | Fibronectin type III domain | 453 | 4534 |
| PF07690.15 | Major Facilitator Superfamily | 401 | 2385 |
| PF00125.23 | Core histone H2A/H2B/H3/H4 | 397 | 948 |
| PF00071.21 | Ras family | 356 | 1765 |
| PF00183.17 | Hsp90 protein | 349 | 166 |
| PF15440.5 | THRAP3/BCLAF1 family | 333 | 10 |
| PF00307.30 | Calponin homology (CH) domain | 297 | 1094 |
| PF00118.23 | TCP-1/cpn60 chaperonin family | 287 | 531 |
| PF00630.18 | Filamin/ABP280 repeat | 272 | 987 |
| PF00270.28 | DEAD/DEAH box helicase | 251 | 2090 |
| PF00428.18 | 60s Acidic ribosomal protein | 249 | 225 |
| PF00091.24 | Tubulin/FtsZ family, GTPase domain | 247 | 396 |
| PF00433.23 | Protein kinase C terminal domain | 245 | 369 |
| PF03953.16 | Tubulin C-terminal domain | 228 | 357 |
| PF00038.20 | Intermediate filament protein | 222 | 202 |
| PF00400.31 | WD domain, G-beta repeat | 221 | 13638 |
| PF16182.4 | Putative adherens-junction anchoring region of AbLIM | 220 | 18 |
| PF00225.22 | Kinesin motor domain | 215 | 841 |
| PF00435.20 | Spectrin repeat | 208 | 2243 |
| PF00151.18 | Lipase | 206 | 238 |
| PF00244.19 | 14-3-3 protein | 201 | 157 |
| PF00169.28 | PH domain | 200 | 1506 |
| PF00017.23 | SH2 domain | 199 | 718 |
| PF04615.12 | Utp14 protein | 192 | 47 |
| PF05470.11 | Eukaryotic translation initiation factor 3 subunit 8 N-terminus | 181 | 77 |
| PF00013.28 | KH domain | 179 | 1750 |
| PF05793.11 | Transcription initiation factor IIF, alpha subunit (TFIIF-alpha) | 176 | 44 |
| PF02535.21 | ZIP Zinc transporter | 174 | 330 |
| PF01044.18 | Vinculin family | 174 | 105 |
| PF03547.17 | Membrane transport protein | 173 | 157 |
| PF00274.18 | Fructose-bisphosphate aldolase class-I | 173 | 83 |
| PF05001.12 | RNA polymerase Rpb1 C-terminal repeat | 171 | 540 |
| PF02800.19 | Glyceraldehyde 3-phosphate dehydrogenase, C-terminal domain | 170 | 154 |
| PF05110.12 | AF-4 proto-oncoprotein | 163 | 30 |
| PF00168.29 | C2 domain | 162 | 2227 |
| PF00009.26 | Elongation factor Tu GTP binding domain | 161 | 735 |
| PF13499.5 | EF-hand domain pair | 158 | 1791 |
| PF14510.5 | ABC-transporter extracellular N-terminal | 156 | 171 |
| PF12796.6 | Ankyrin repeats (3 copies) | 156 | 3578 |
| PF08065.11 | K167R (NUC007) repeat | 154 | 49 |
| PF03154.14 | Atrophin-1 family | 154 | 27 |
