# Supplementary material for: Conserved phosphorylation hotspots in eukaryotic protein domain families
Source: Nat Commun. 2019 Apr 29;10:1977. doi: 10.1038/s41467-019-09952-x (PMC6488607; doi:10.1038/s41467-019-09952-x)
Supplement: Supplementary file 3 — Description of Additional Supplementary Files [file 41467_2019_9952_MOESM3_ESM.docx]

**Description of Supplementary Files**

**File Name:** Supplementary Data 1

**Description:** List of identified hotspot regions with information on interface contacts and distance to catalytic residues.

**File Name:** Supplementary Data 2

**Description:** List of enrichment plots and structural mapping for all significant hotspot regions.
