# Supplementary material for: Conserved phosphorylation hotspots in eukaryotic protein domain families
Source: Nat Commun. 2019 Apr 29;10:1977. doi: 10.1038/s41467-019-09952-x (PMC6488607; doi:10.1038/s41467-019-09952-x)

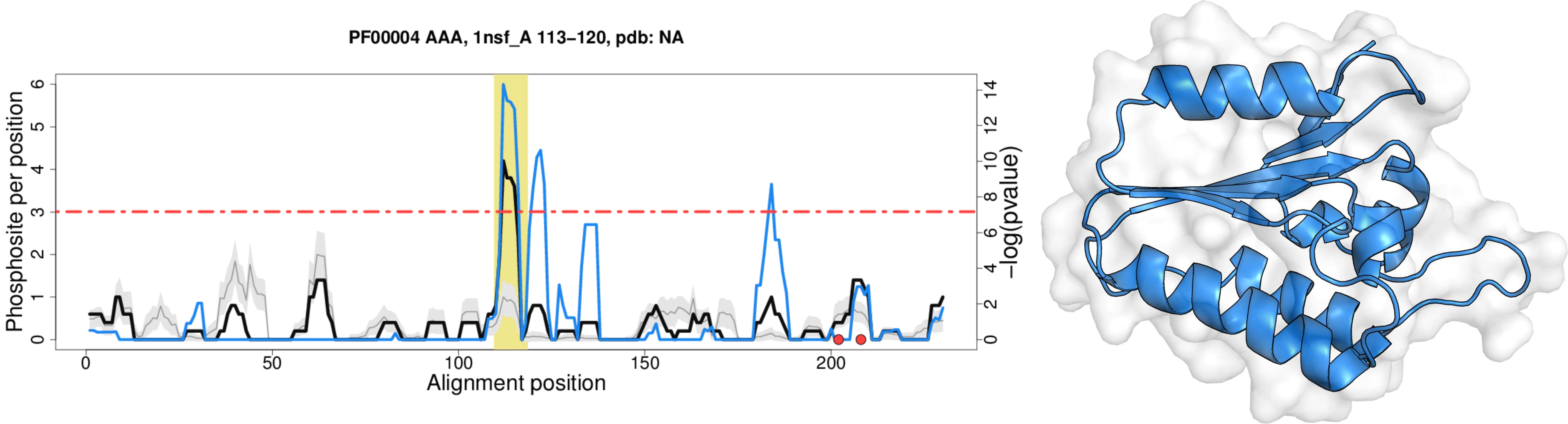
PF00004 AAA, 1nsf_A 113–120, pdb: NA
Phosphosite per position
0
1
2
3
4
5
6
−log(pvalue)
0
2
4
6
8
10
12
14
0
50
100
150
200
Alignment position

PF00005 ABC_tran, 5kpi_A 288–300, pdb: NA

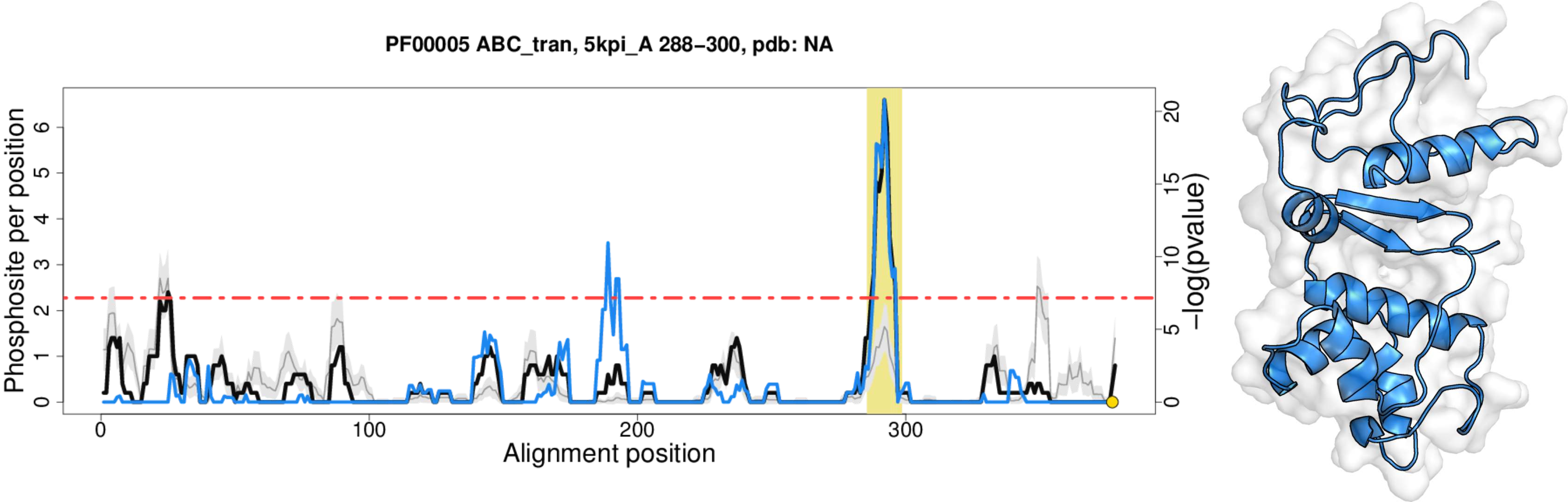

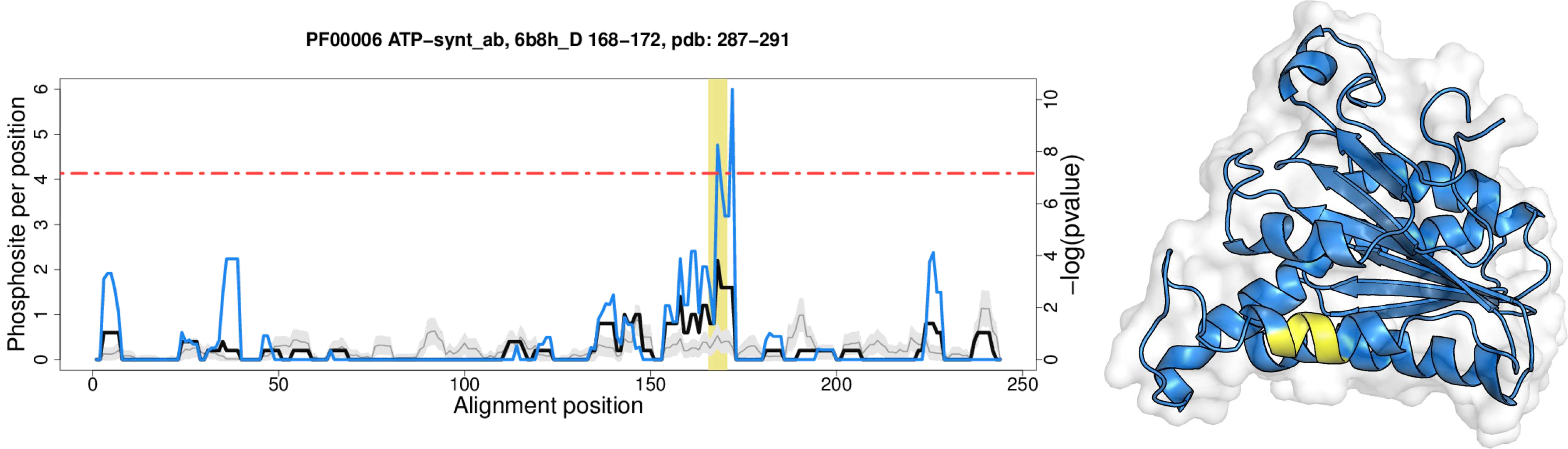

PF00006 ATP−synt_ab, 6b8h_D 168−172, pdb: 287−291
Phosphosite per position
0
1
2
3
4
5
6
−log(pvalue)
0
2
4
6
8
10
Alignment position
0
50
100
150
200
250

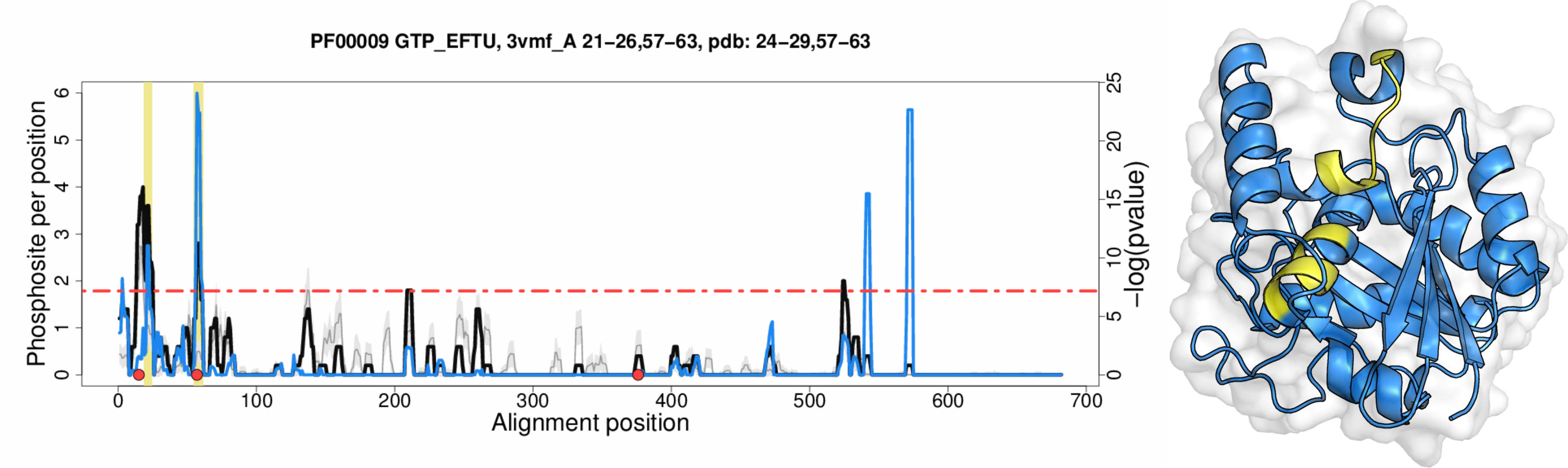
PF00009 GTP_EFTU, 3vmf_A 21–26,57–63, pdb: 24–29,57–63
Phosphosite per position
0
1
2
3
4
5
6
−log(pvalue)
0
5
10
15
20
25
Alignment position
0
100
200
300
400
500
600
700

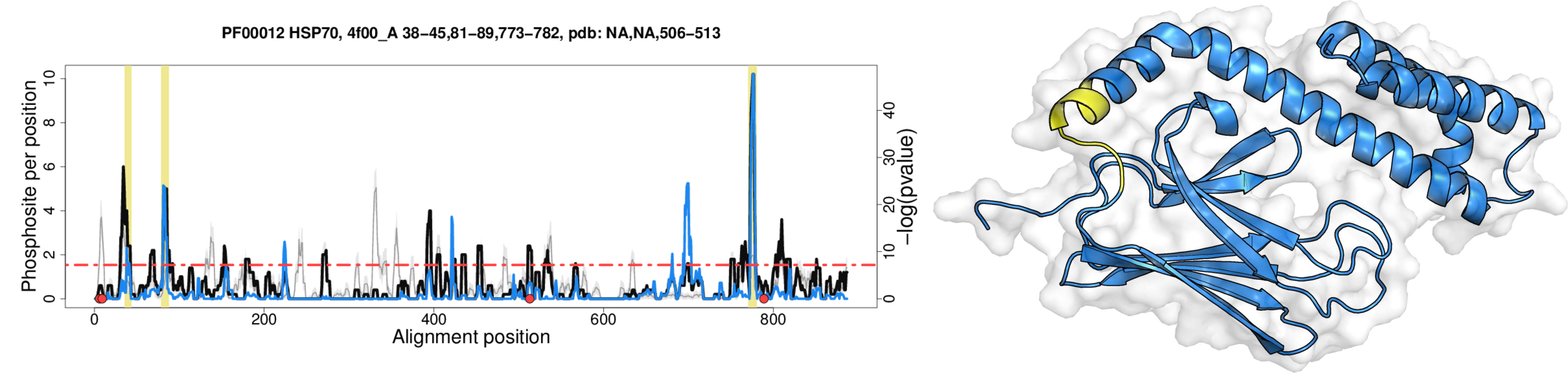

PF00012 HSP70, 4f00_A 38−45,81−89,773−782, pdb: NA,NA,506−513
Phosphosite per position
Alignment position
−log(pvalue)
0
2
4
6
8
10
0
200
400
600
800
0
10
20
30
40

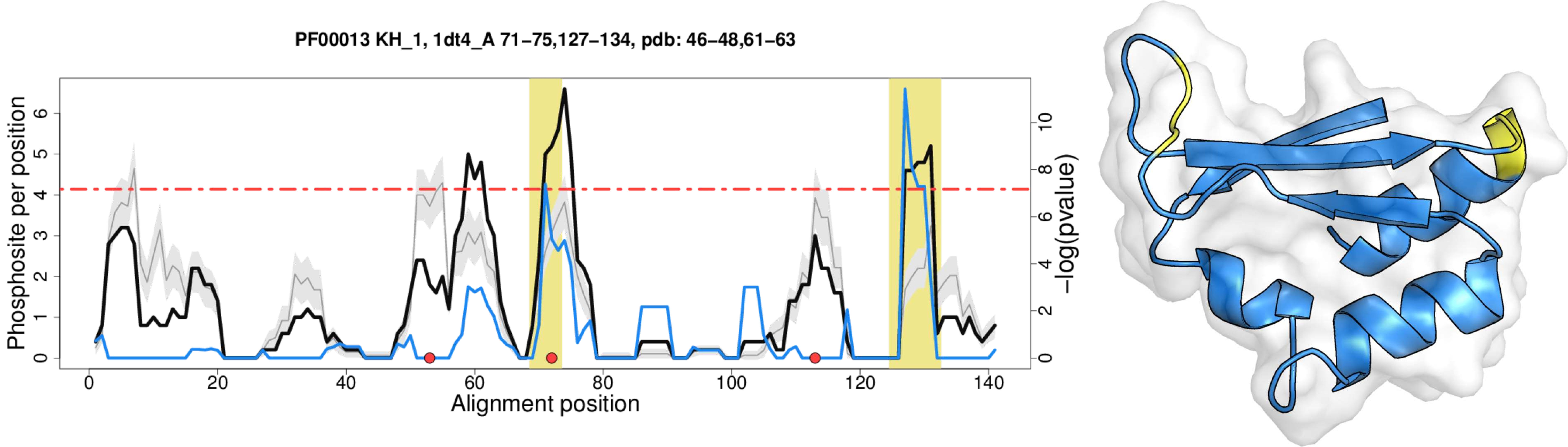
PF00013 KH_1, 1dt4_A 71−75,127−134, pdb: 46−48,61−63
Phosphosite per position
0
1
2
3
4
5
6
−log(pvalue)
0
2
4
6
8
10
0
20
40
60
80
100
120
140
Alignment position

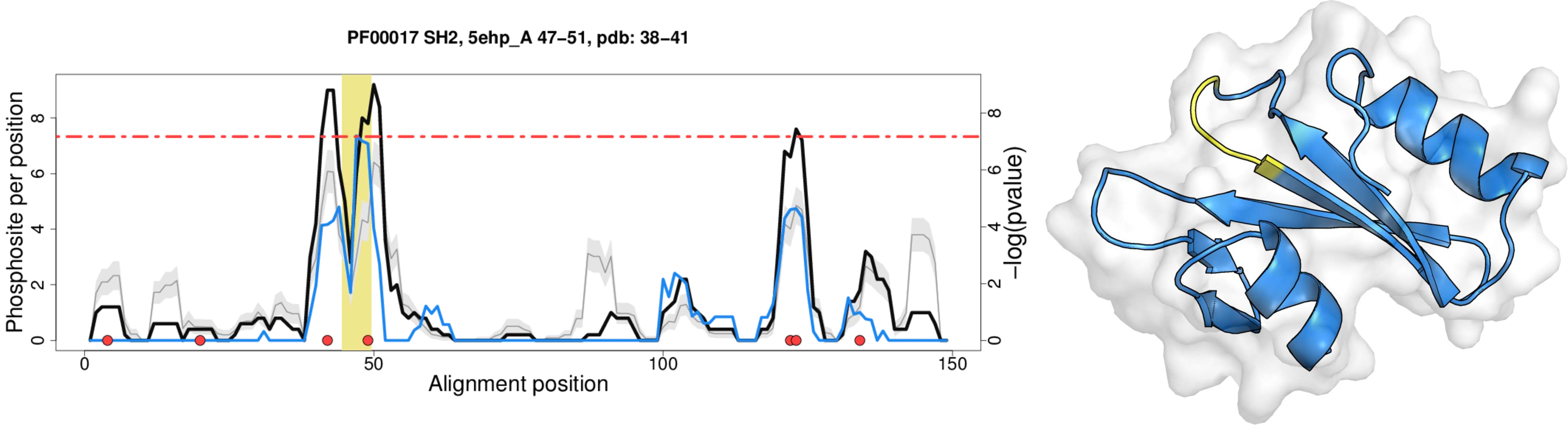
PF00017 SH2, 5ehp_A 47–51, pdb: 38–41
Phosphosite per position
–log(pvalue)
Alignment position
0
50
100
150
0
2
4
6
8

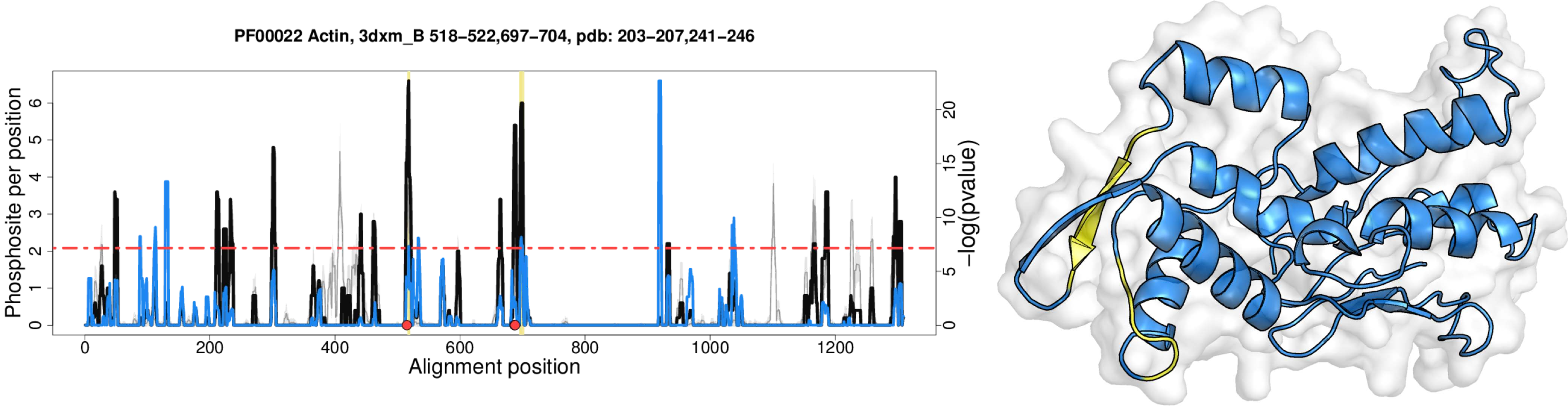

PF00022 Actin, 3dxm_B 518–522,697–704, pdb: 203–207,241–246
Phosphosite per position
0
1
2
3
4
5
6
0
200
400
600
800
1000
1200
Alignment position
–log(pvalue)
0
5
10
15
20

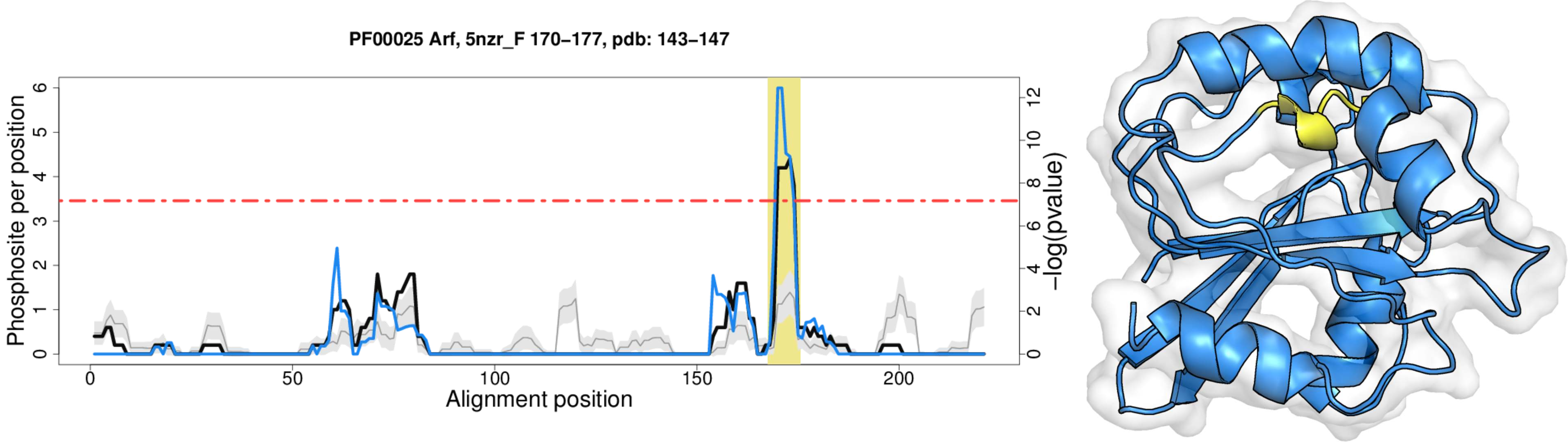
PF00025 Arf, 5nzr_F 170–177, pdb: 143–147
Phosphosite per position
–log(pvalue)
Alignment position

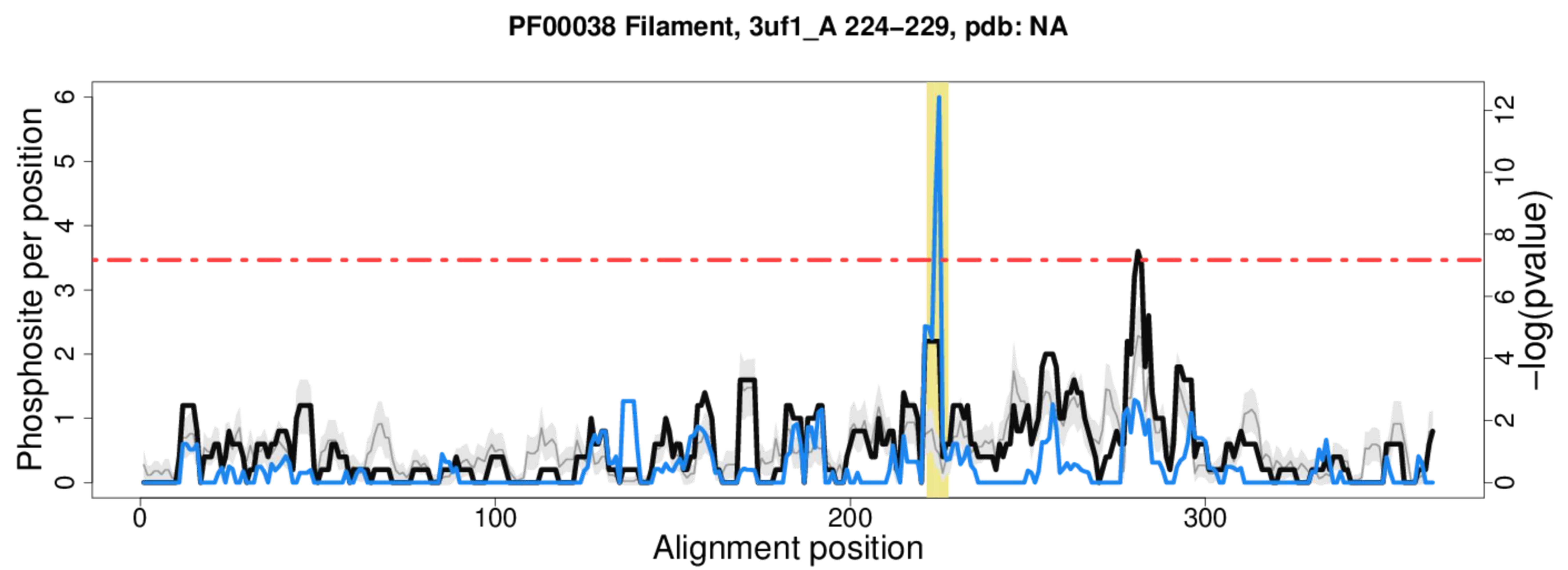

PF00038 Filament, 3uf1_A 224–229, pdb: NA
Phosphosite per position
−log(pvalue)
Alignment position
0
1
2
3
4
5
6
0
2
4
6
8
10
12
0
100
200
300

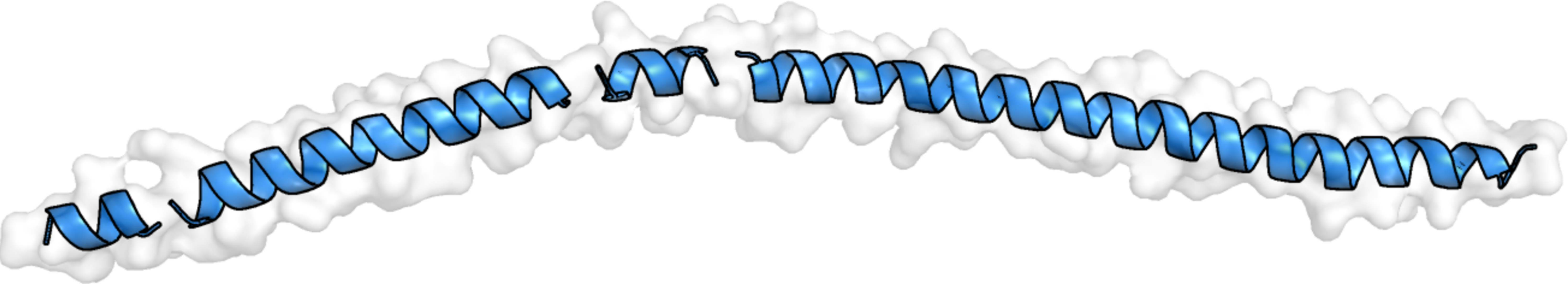

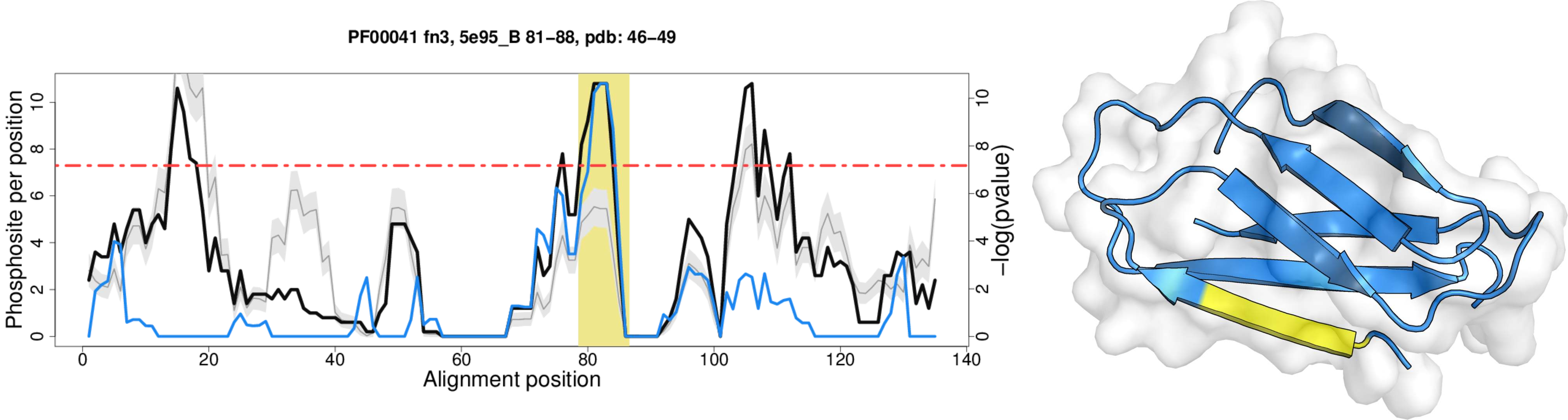
PF00041 fn3, 5e95_B 81–88, pdb: 46–49
Phosphosite per position
0
2
4
6
8
10
-log(pvalue)
0
20
40
60
80
100
120
140
Alignment position

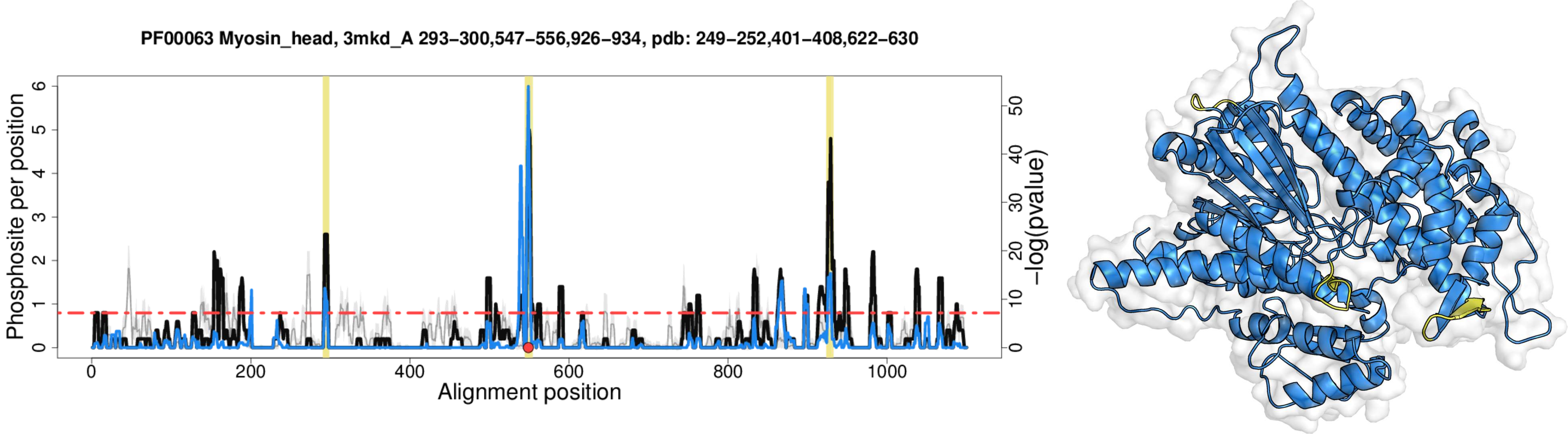
PF00063 Myosin_head, 3mkd_A 293–300,547–556,926–934, pdb: 249–252,401–408,622–630
Phosphosite per position
0
1
2
3
4
5
6
Alignment position
0
200
400
600
800
1000
−log(pvalue)
0
10
20
30
40
50

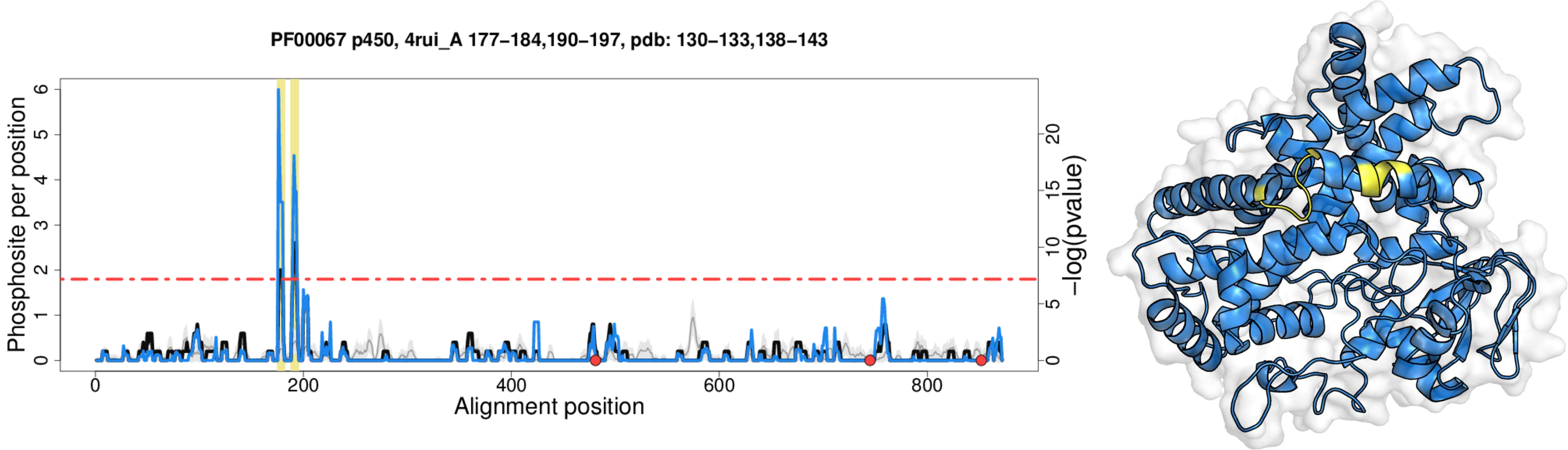
PF00067 p450, 4rui_A 177–184,190–197, pdb: 130–133,138–143
Phosphosite per position
–log(pvalue)
Alignment position
0
1
2
3
4
5
6
0
200
400
600
800
0
5
10
15
20

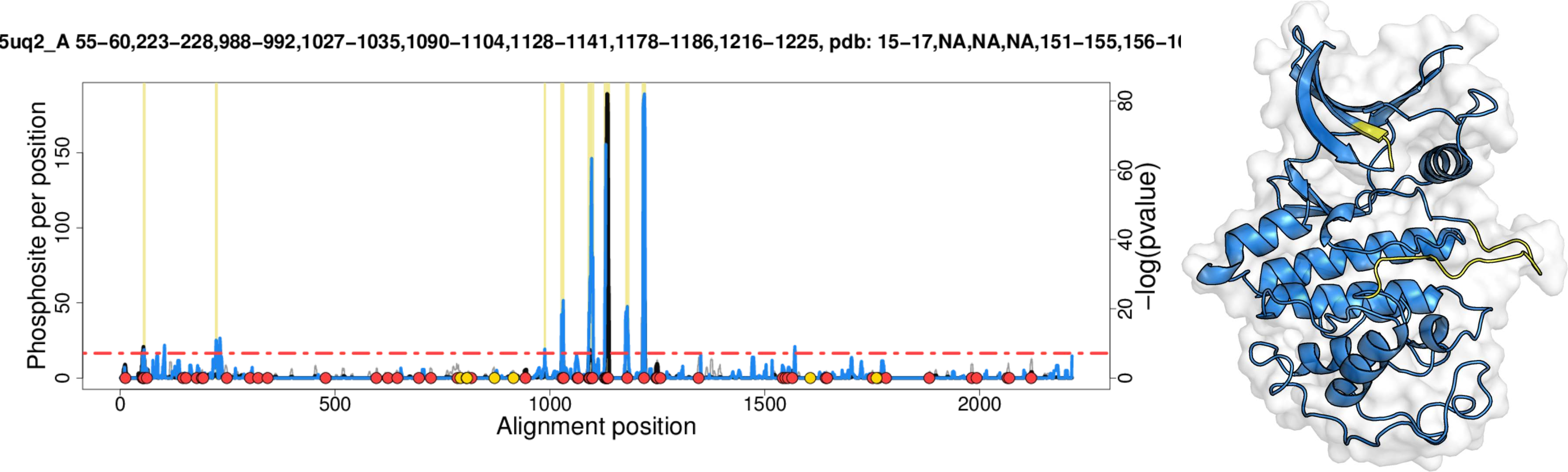
5uq2_A 55−60,223−228,988−992,1027−1035,1090−1104,1128−1141,1178−1186,1216−1225, pdb: 15−17,NA,NA,NA,151−155,156−1
Phosphosite per position
0
50
100
150
−log(pvalue)
0
20
40
60
80
Alignment position
0
500
1000
1500
2000

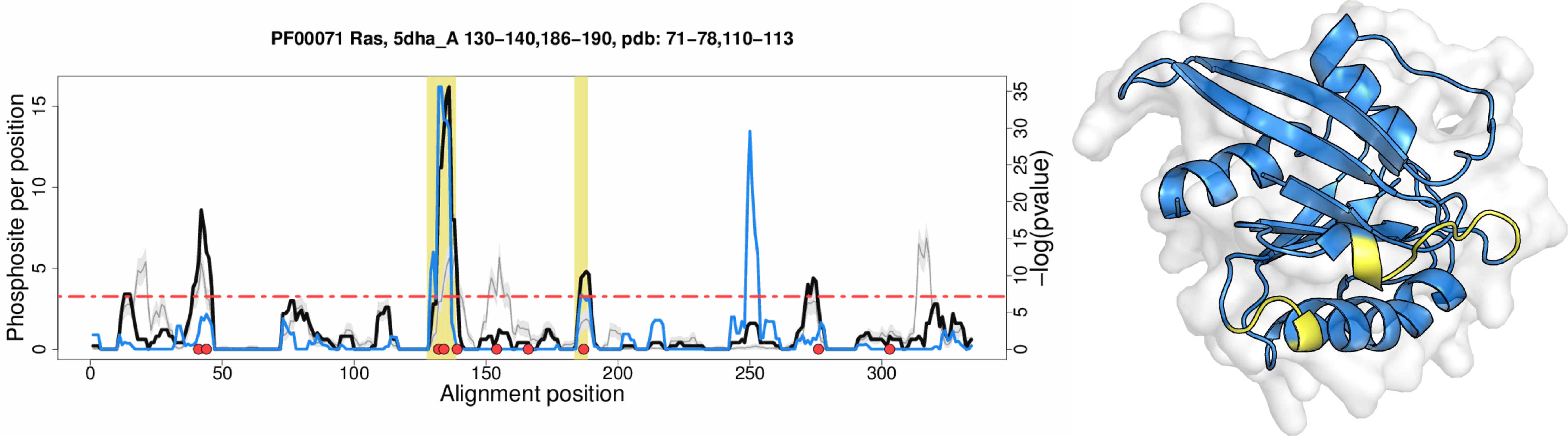
PF00071 Ras, 5dha_A 130−140,186−190, pdb: 71−78,110−113
Phosphosite per position
−log(pvalue)
Alignment position
0
5
10
15
0
50
100
150
200
250
300
0
5
10
15
20
25
30
35

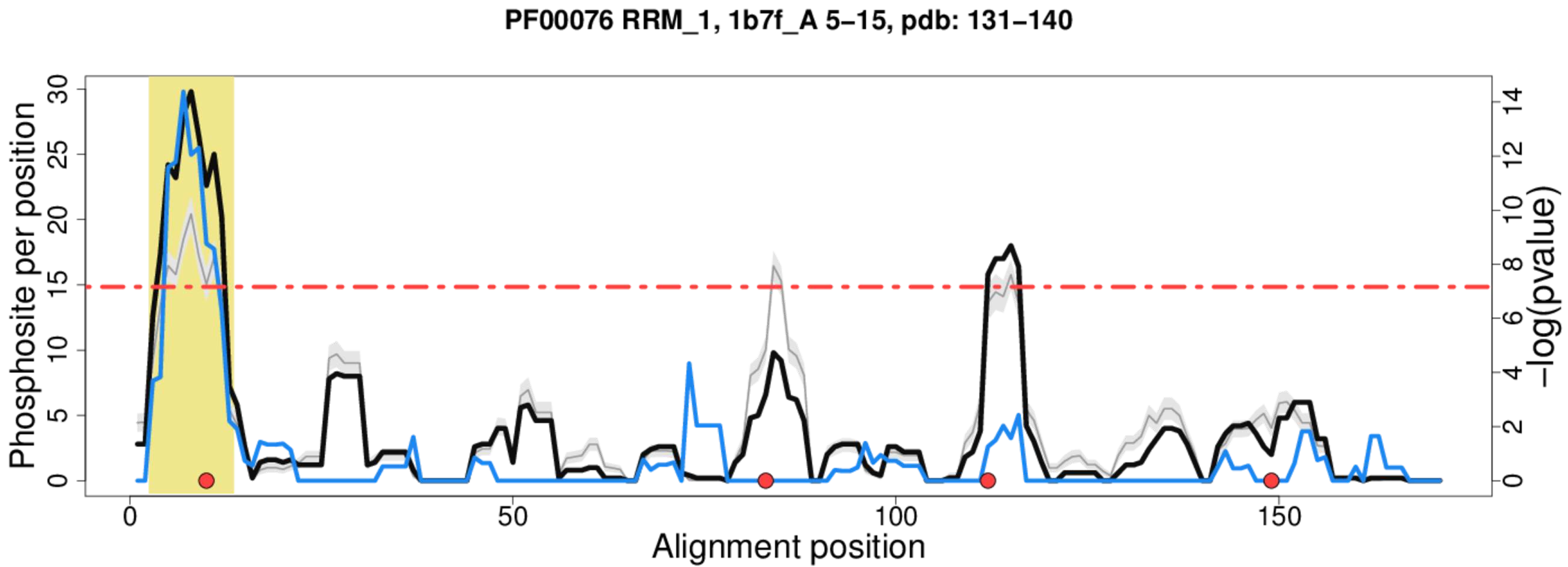

PF00076 RRM_1, 1b7f_A 5–15, pdb: 131–140
Phosphosite per position
−log(pvalue)
Alignment position
0
50
100
150
0
5
10
15
20
25
30
0
2
4
6
8
10
12
14

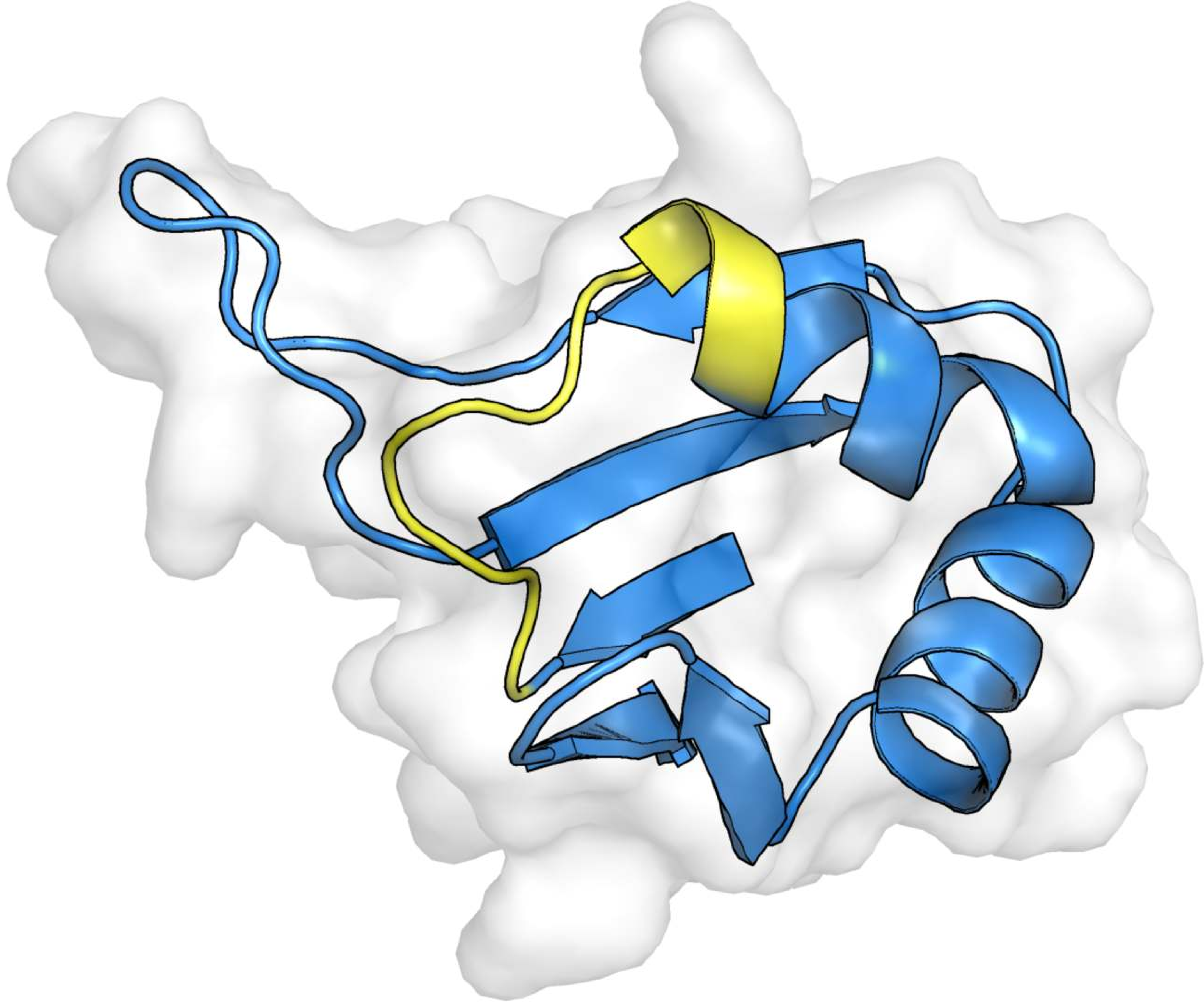

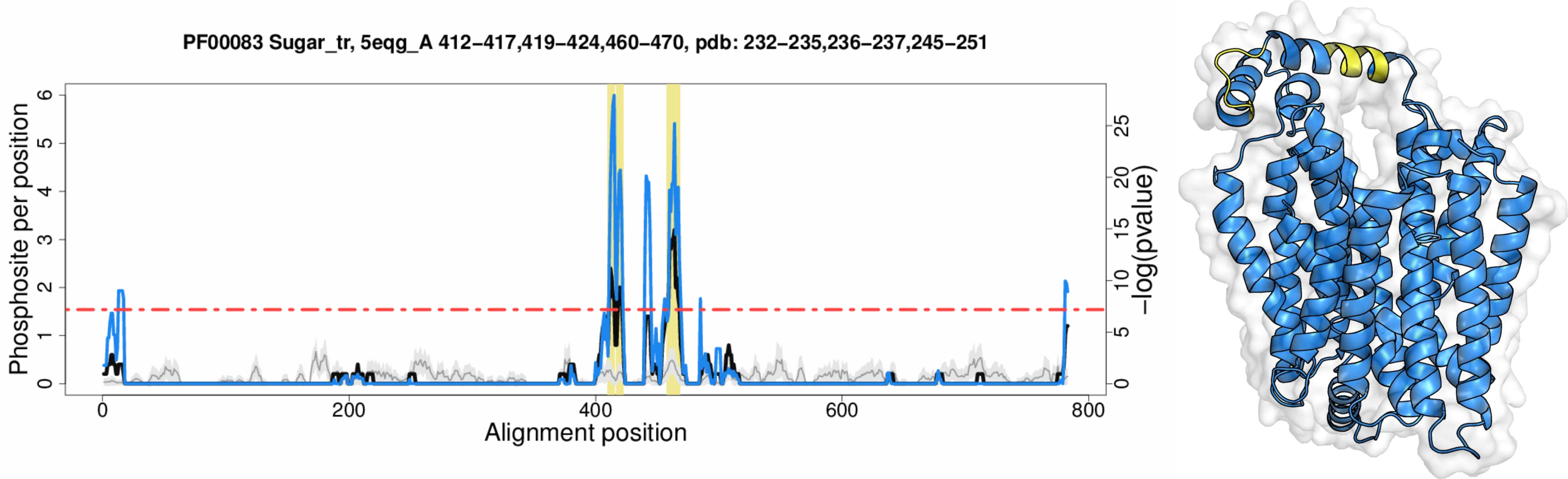

PF00083 Sugar_tr, 5eqg_A 412−417,419−424,460−470, pdb: 232−235,236−237,245−251
Phosphosite per position
−log(pvalue)
Alignment position

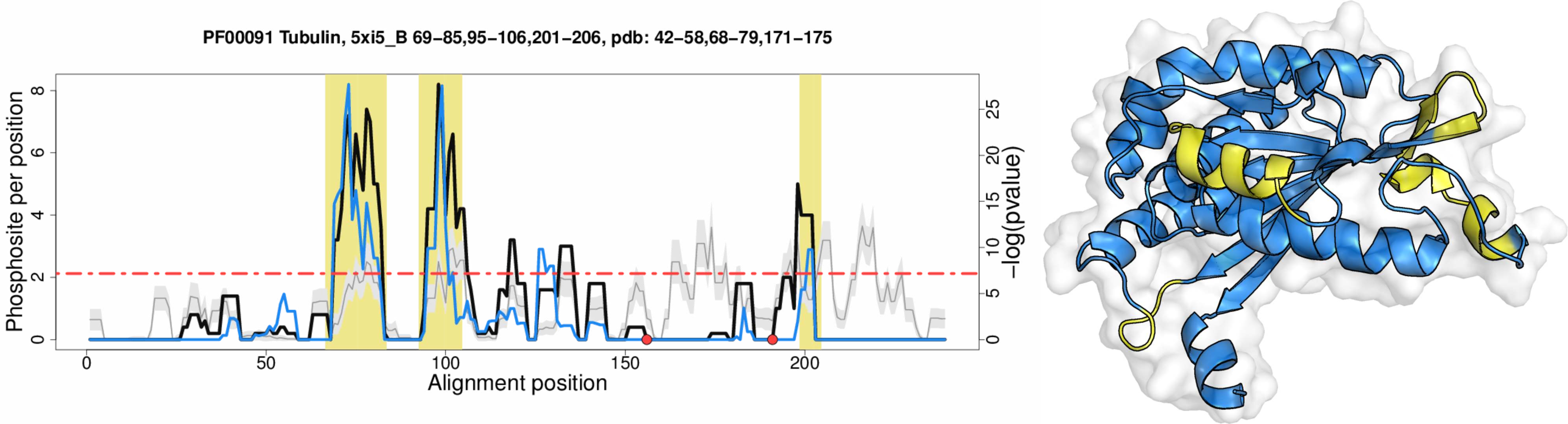
PF00091 Tubulin, 5xi5_B 69−85,95−106,201−206, pdb: 42−58,68−79,171−175
Phosphosite per position
−log(pvalue)
Alignment position
0
50
100
150
200
0
2
4
6
8
0
5
10
15
20
25

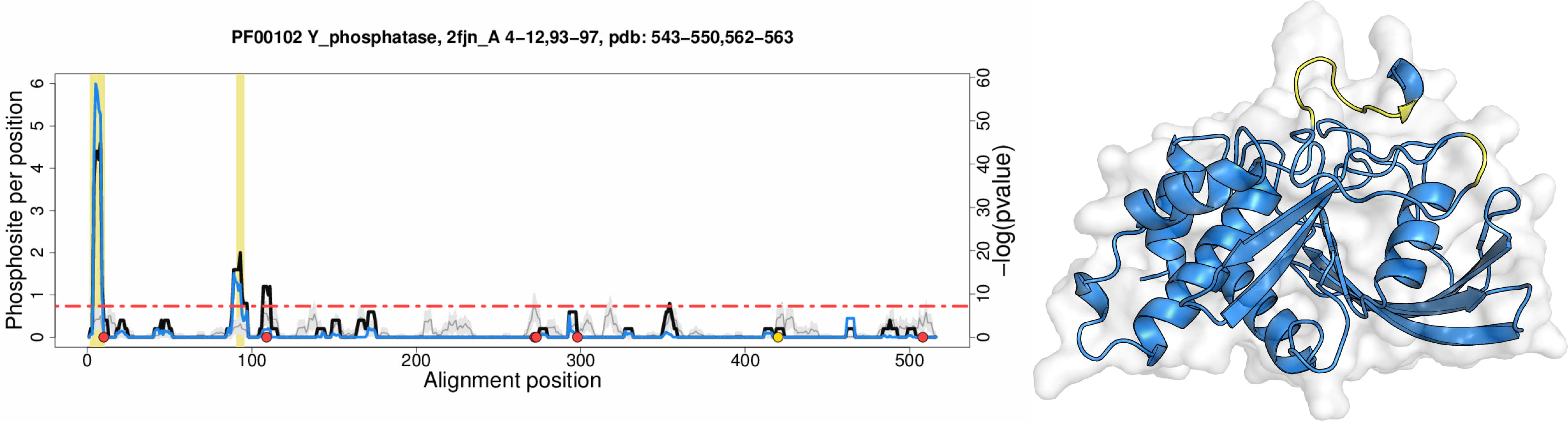
PF00102 Y_phosphatase, 2fjn_A 4−12,93−97, pdb: 543−550,562−563
Phosphosite per position
−log(pvalue)
Alignment position
0
100
200
300
400
500
0
1
2
3
4
5
6
0
10
20
30
40
50
60

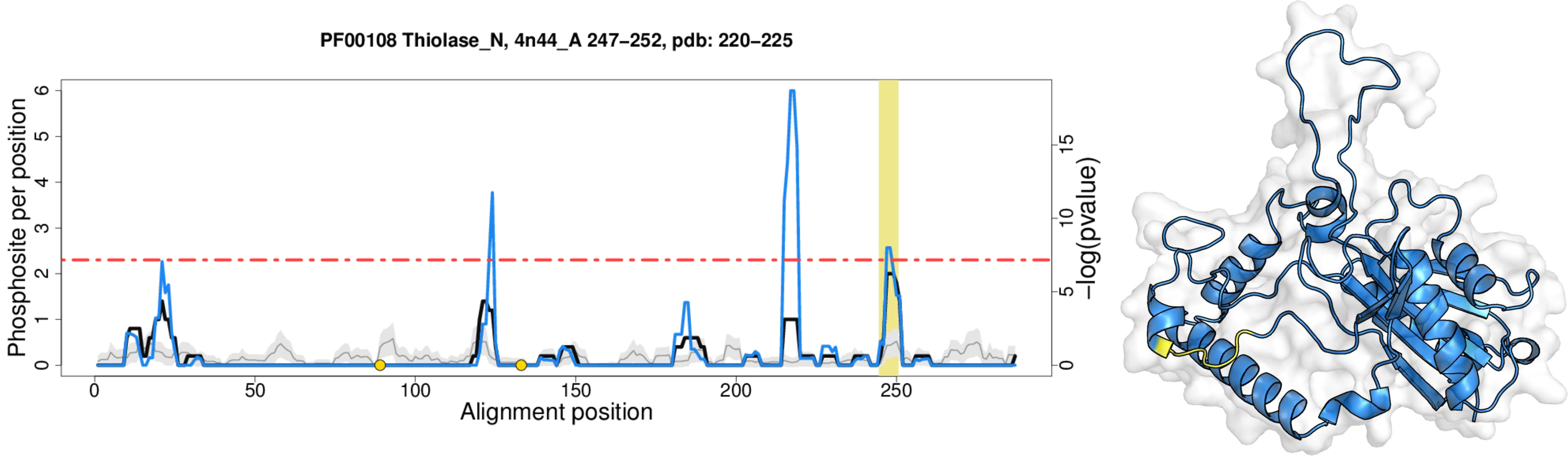
PF00108 Thiolase_N, 4n44_A 247−252, pdb: 220−225
Phosphosite per position
Alignment position
−log(pvalue)
0
1
2
3
4
5
6
50
100
150
200
250
10
15

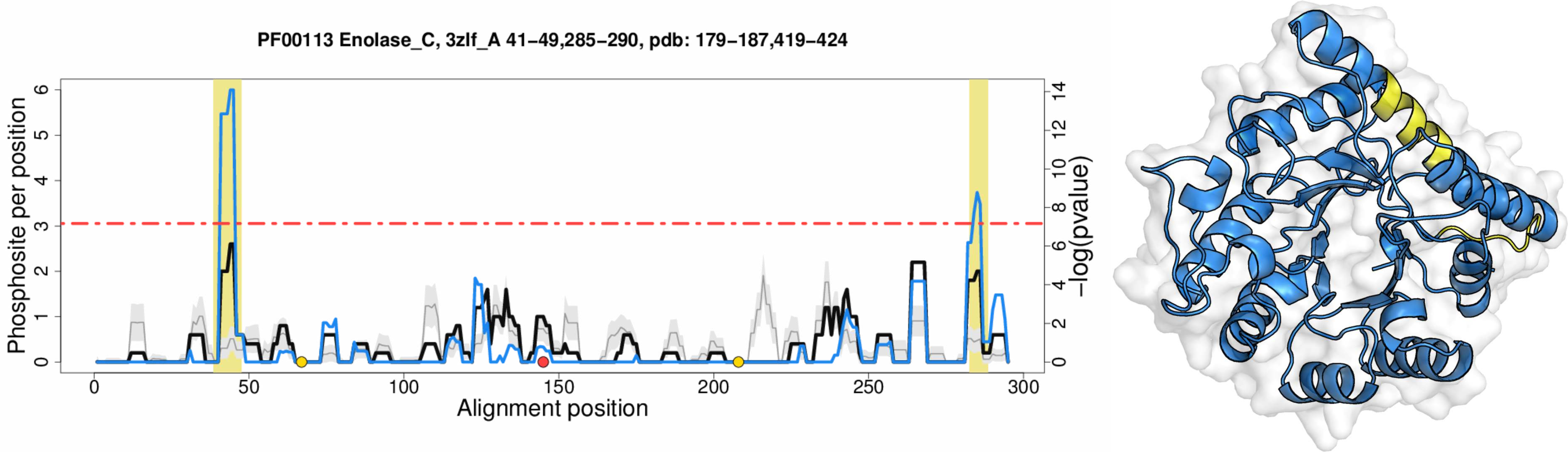
PF00113 Enolase_C, 3zlf_A 41−49,285−290, pdb: 179−187,419−424
Phosphosite per position
0
1
2
3
4
5
6
−log(pvalue)
0
2
4
6
8
10
12
14
Alignment position
0
50
100
150
200
250
300

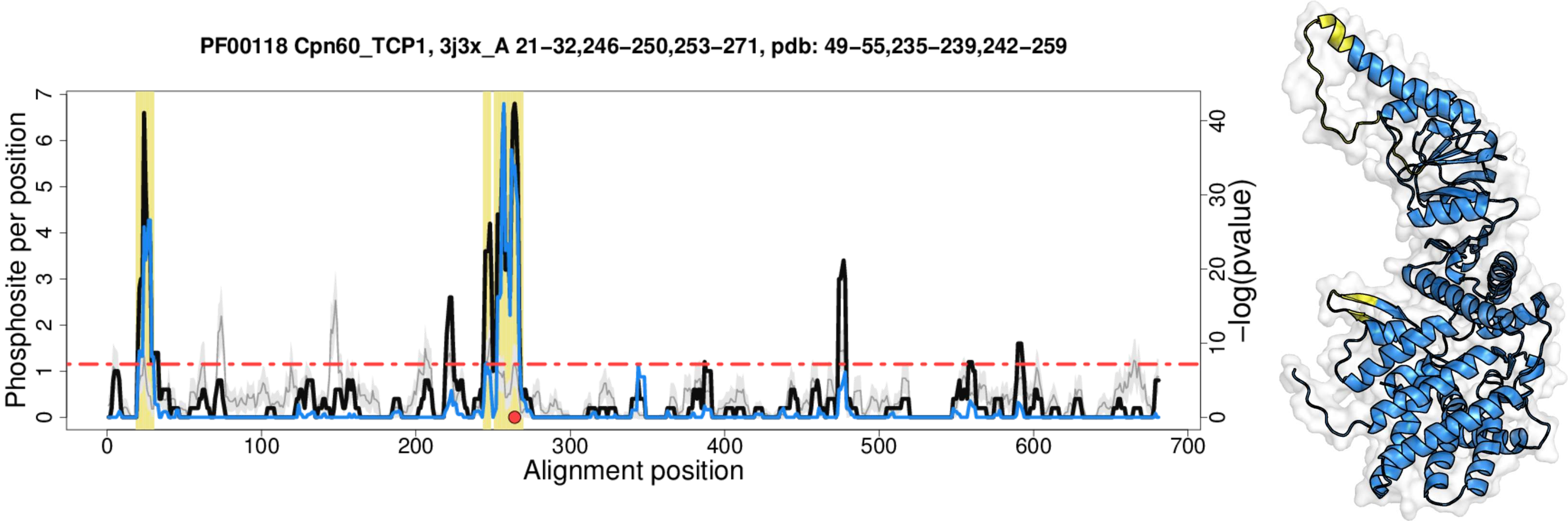
PF00118 Cpn60_TCP1, 3j3x_A 21−32,246−250,253−271, pdb: 49−55,235−239,242−259
Phosphosite per position
Alignment position
−log(pvalue)
0 1 2 3 4 5 6 7
0 100 200 300 400 500 600 700
0 10 20 30 40

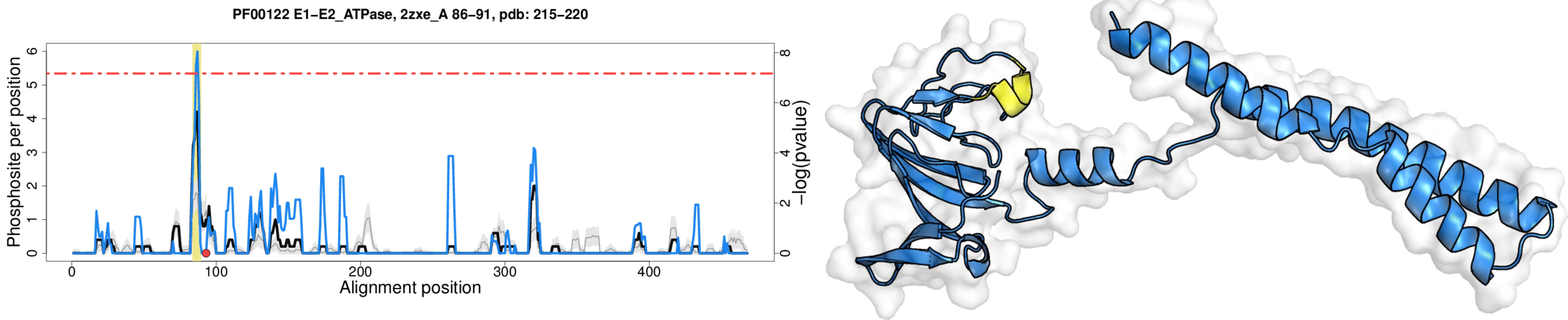
PF00122 E1−E2_ATPase, 2zxe_A 86−91, pdb: 215−220
Phosphosite per position
−log(pvalue)
Alignment position
0
100
200
300
400
0
1
2
3
4
5
6
0
2
4
6
8

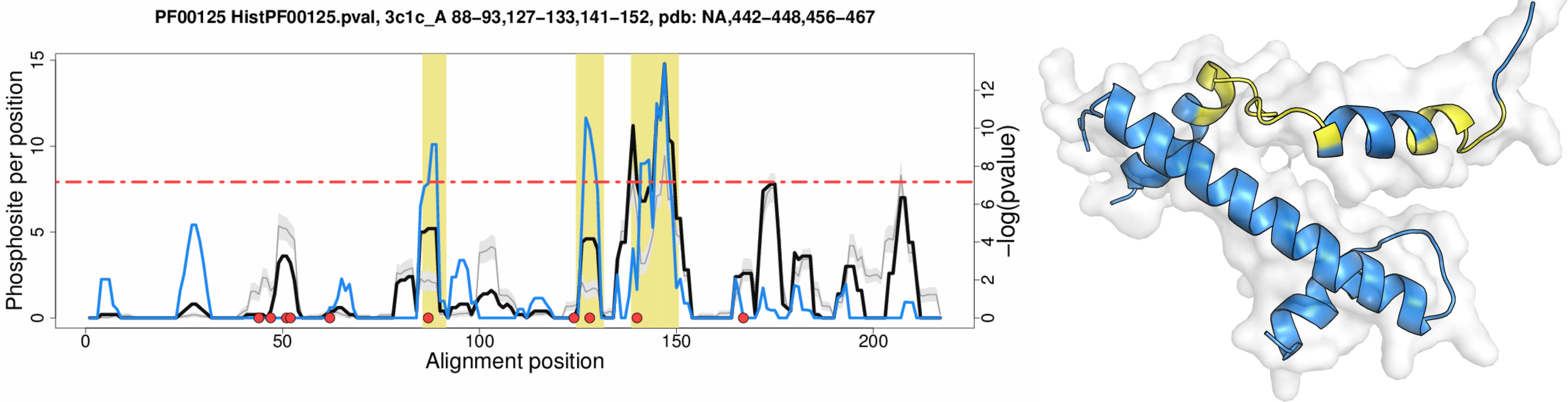
PF00125 HistPF00125.pval, 3c1c_A 88–93,127–133,141–152, pdb: NA,442–448,456–467
Phosphosite per position
0
5
10
15
Alignment position
0
50
100
150
200
−log(pvalue)
0
2
4
6
8
10
12

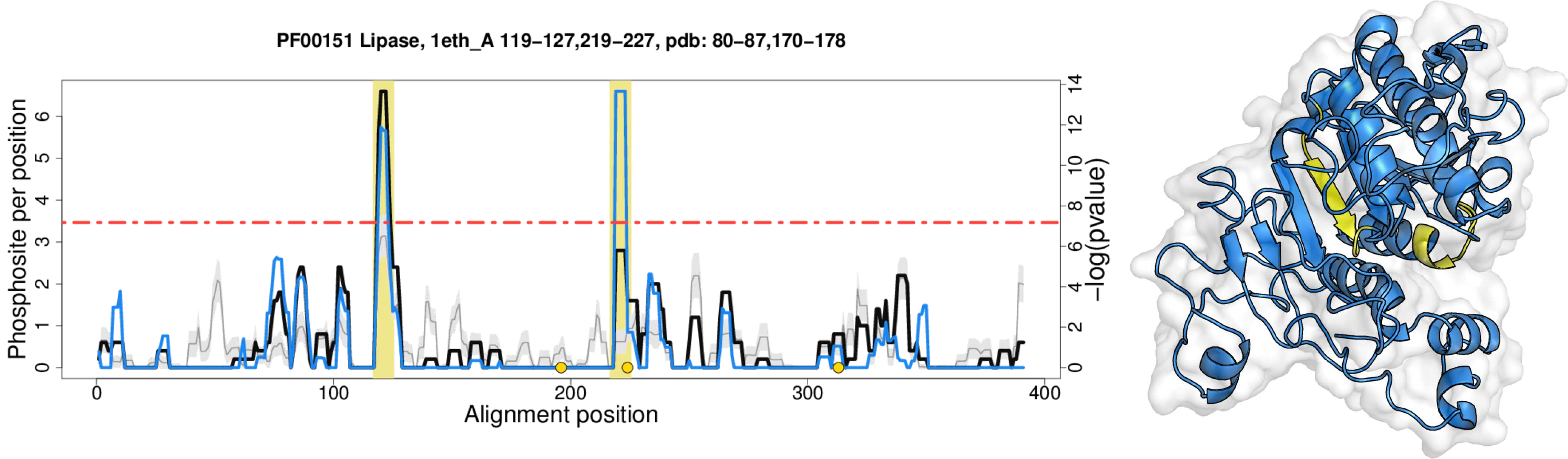
PF00151 Lipase, 1eth_A 119-127,219-227, pdb: 80-87,170-178
Phosphosite per position
-log(pvalue)
Alignment position
0
1
2
3
4
5
6
0
2
4
6
8
10
12
14
0
100
200
300
400

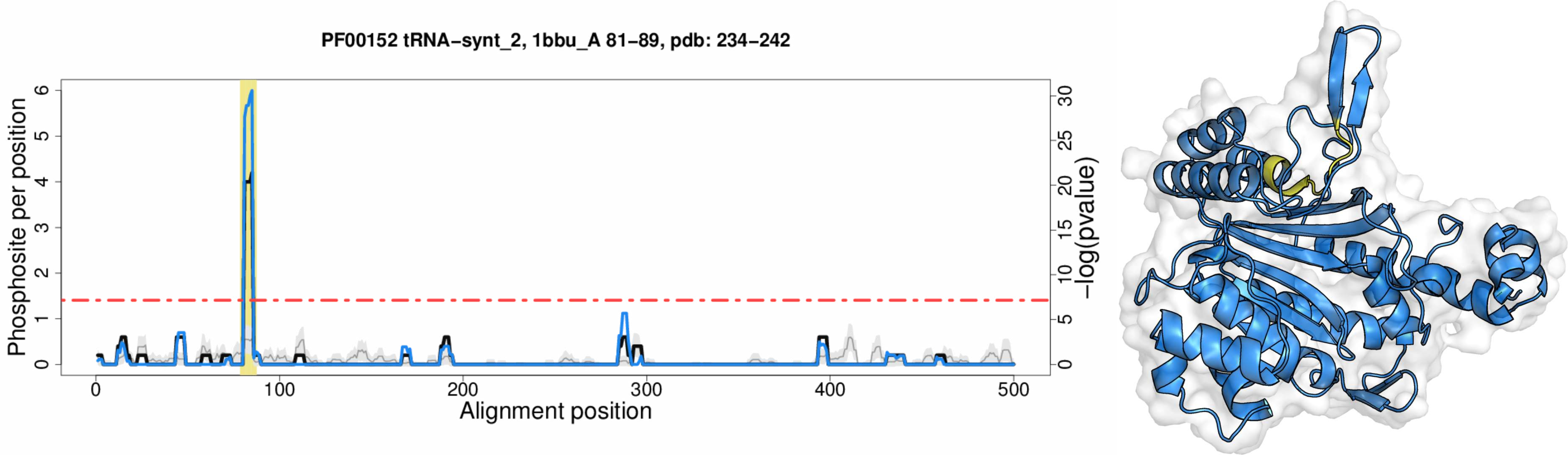

PF00152 tRNA−synt_2, 1bbu_A 81−89, pdb: 234−242
Phosphosite per position
0
1
2
3
4
5
6
−log(pvalue)
0
5
10
15
20
25
30
0
100
200
300
400
500
Alignment position

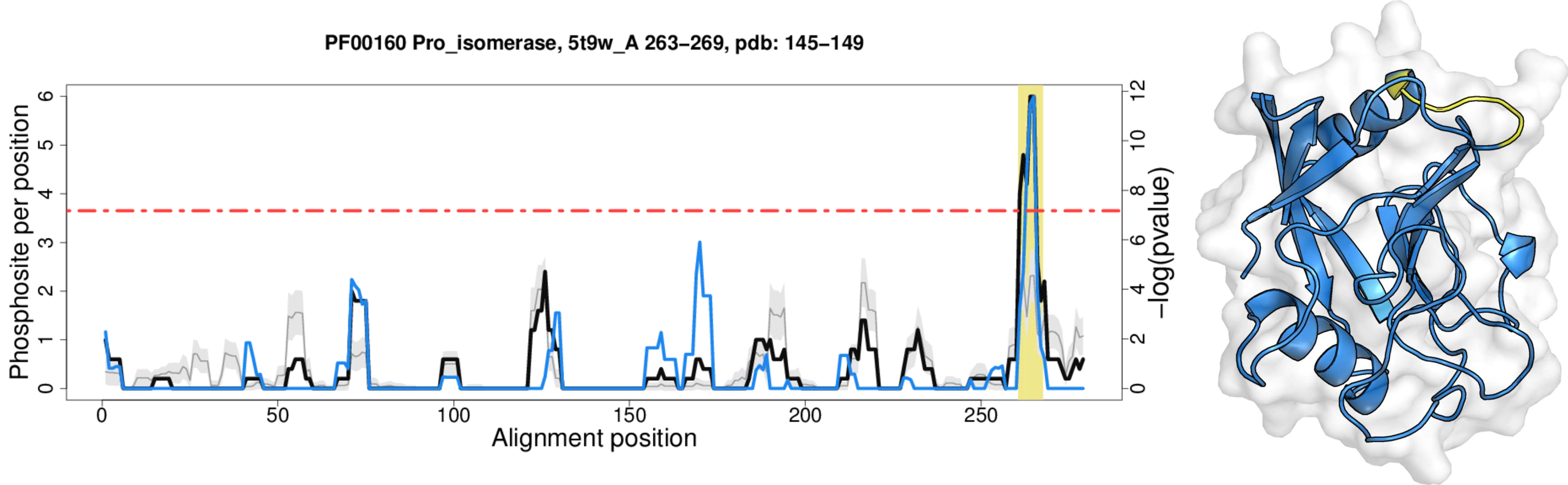

PF00160 Pro_isomerase, 5t9w_A 263–269, pdb: 145–149
Phosphosite per position
0
1
2
3
4
5
6
Alignment position
0
50
100
150
200
250
–log(pvalue)
0
2
4
6
8
10
12

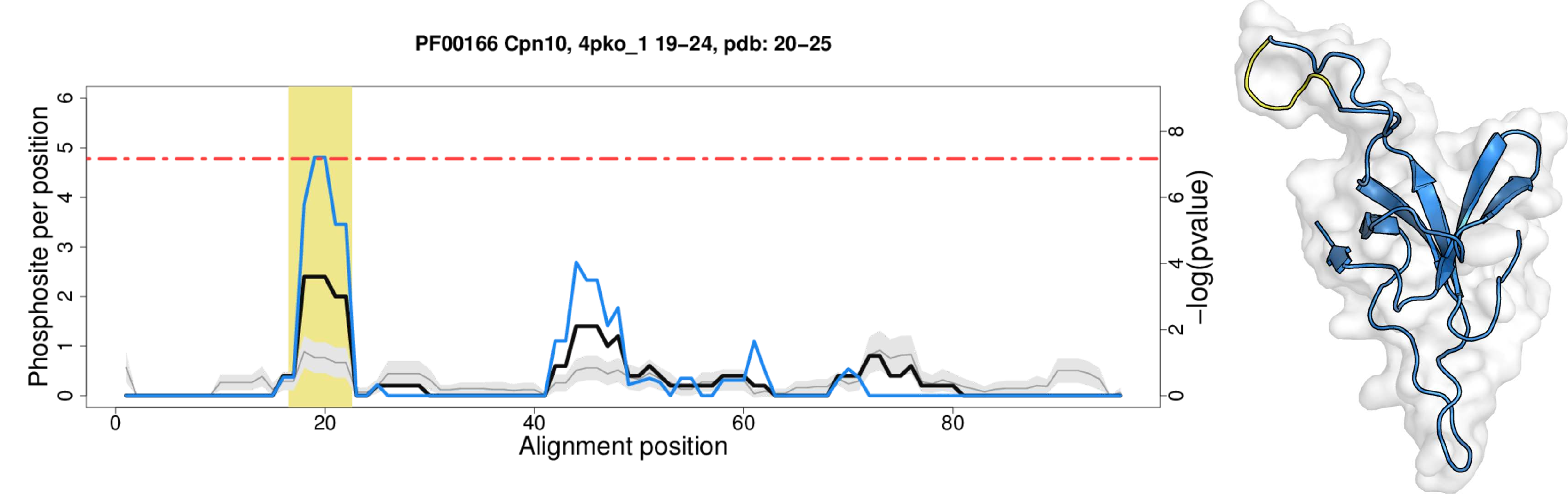

PF00166 Cpn10, 4pko_1 19–24, pdb: 20–25
Phosphosite per position
−log(pvalue)
Alignment position
0
20
40
60
80
0
1
2
3
4
5
6
0
2
4
6
8

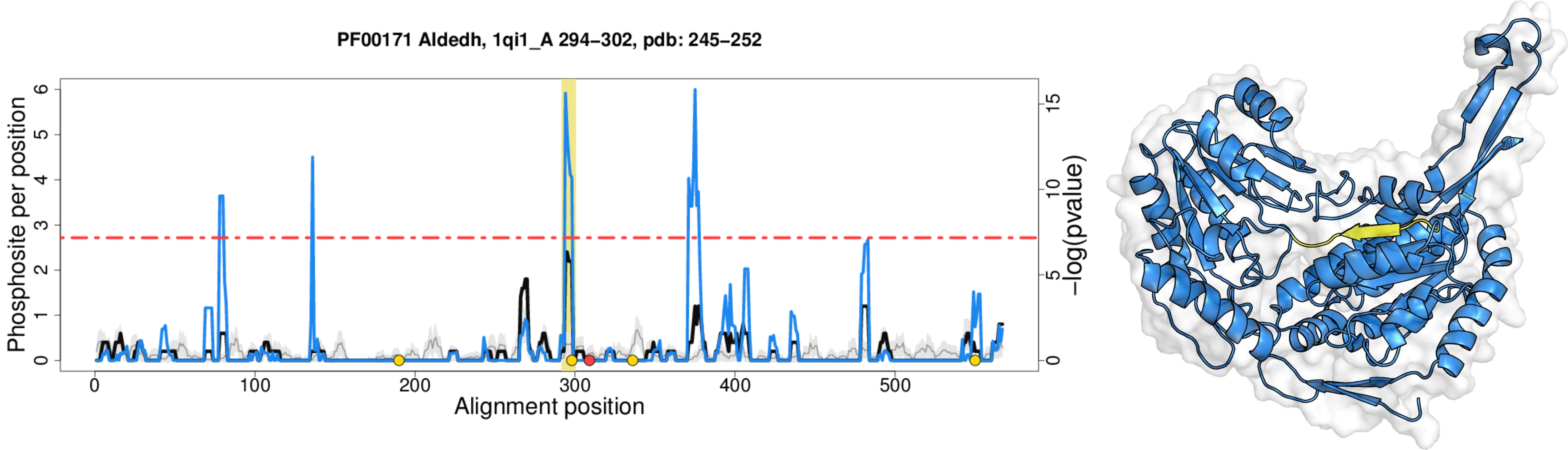
PF00171 Aldedh, 1qi1_A 294–302, pdb: 245–252
Phosphosite per position
0
1
2
3
4
5
6
−log(pvalue)
0
5
10
15
0
100
200
300
400
500
Alignment position

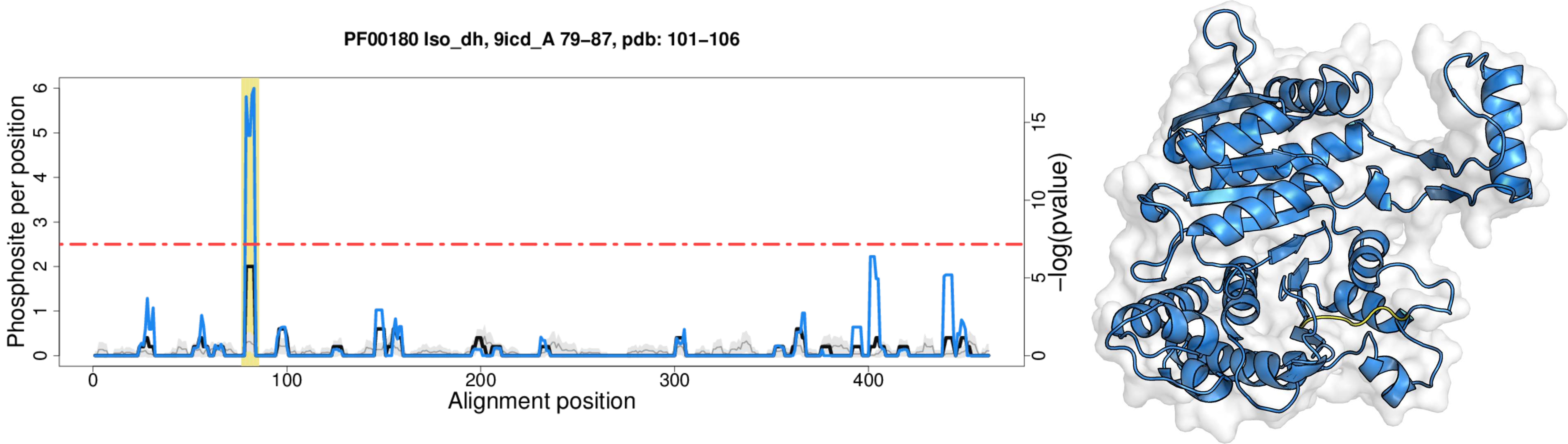
PF00180 Iso_dh, 9icd_A 79–87, pdb: 101–106
Phosphosite per position
0
1
2
3
4
5
6
−log(pvalue)
0
5
10
15
Alignment position
0
100
200
300
400

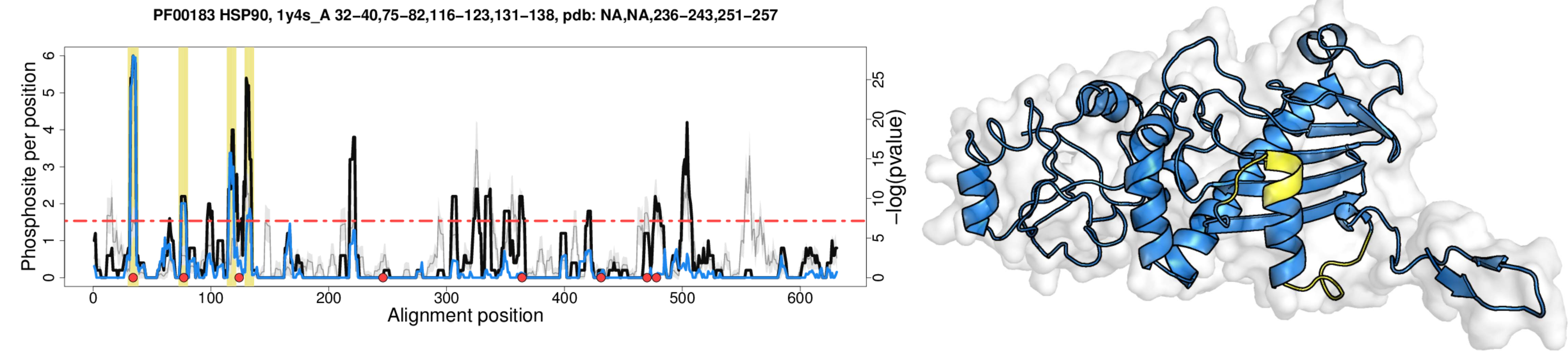
PF00183 HSP90, 1y4s_A 32−40,75−82,116−123,131−138, pdb: NA,NA,236−243,251−257
Phosphosite per position
0
1
2
3
4
5
6
−log(pvalue)
0
5
10
15
20
25
Alignment position
0
100
200
300
400
500
600

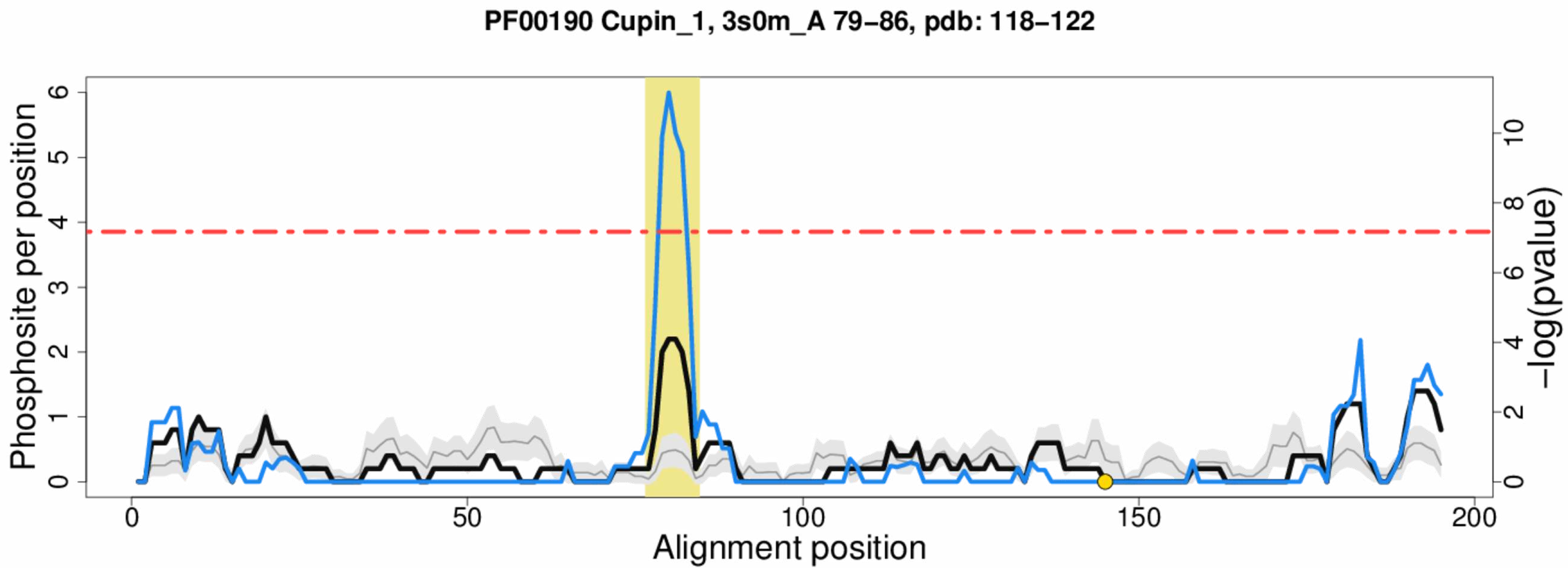

PF00190 Cupin_1, 3s0m_A 79−86, pdb: 118−122
Phosphosite per position
0
1
2
3
4
5
6
Alignment position
0
50
100
150
200
−log(pvalue)
0
2
4
6
8
10

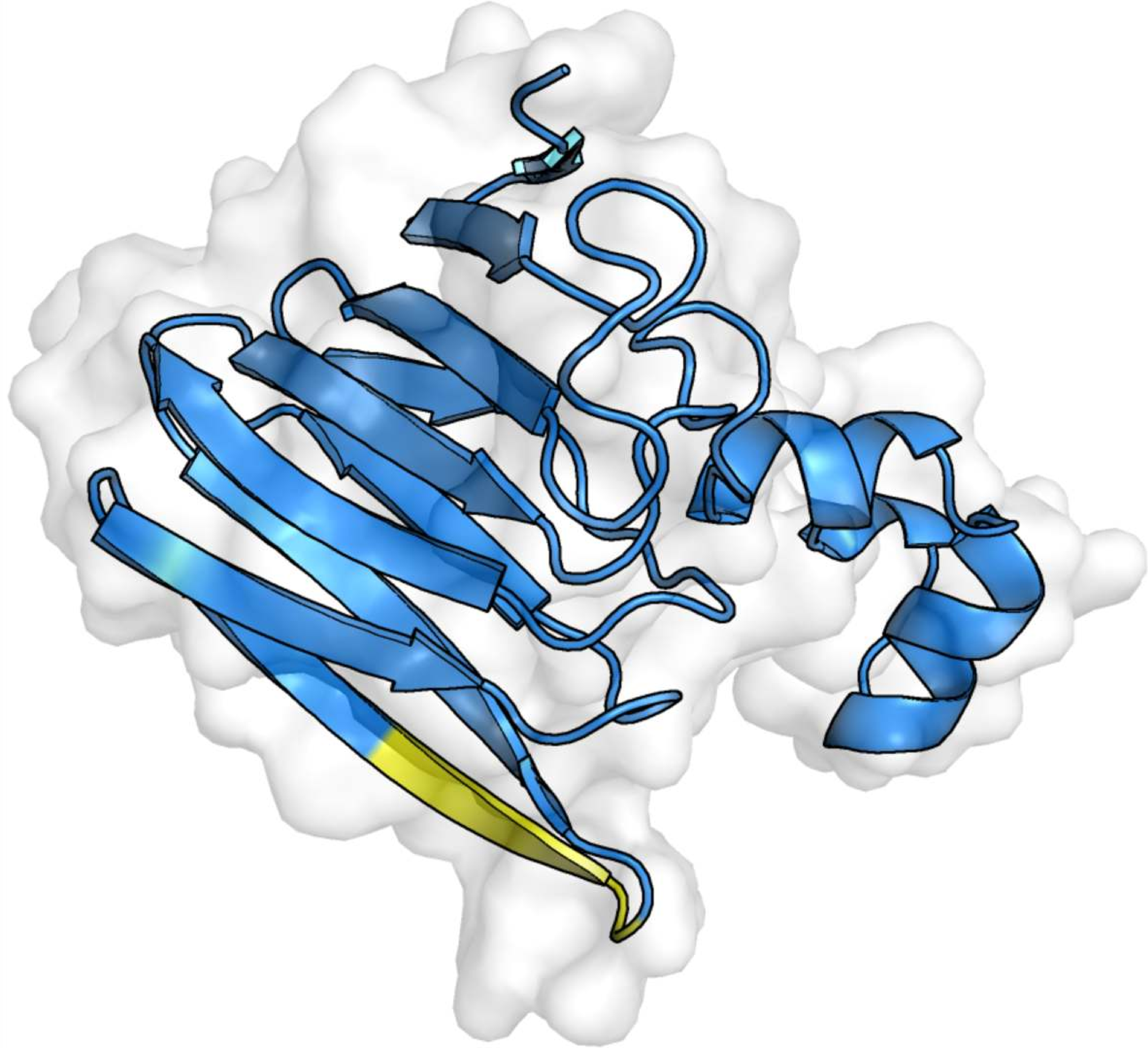

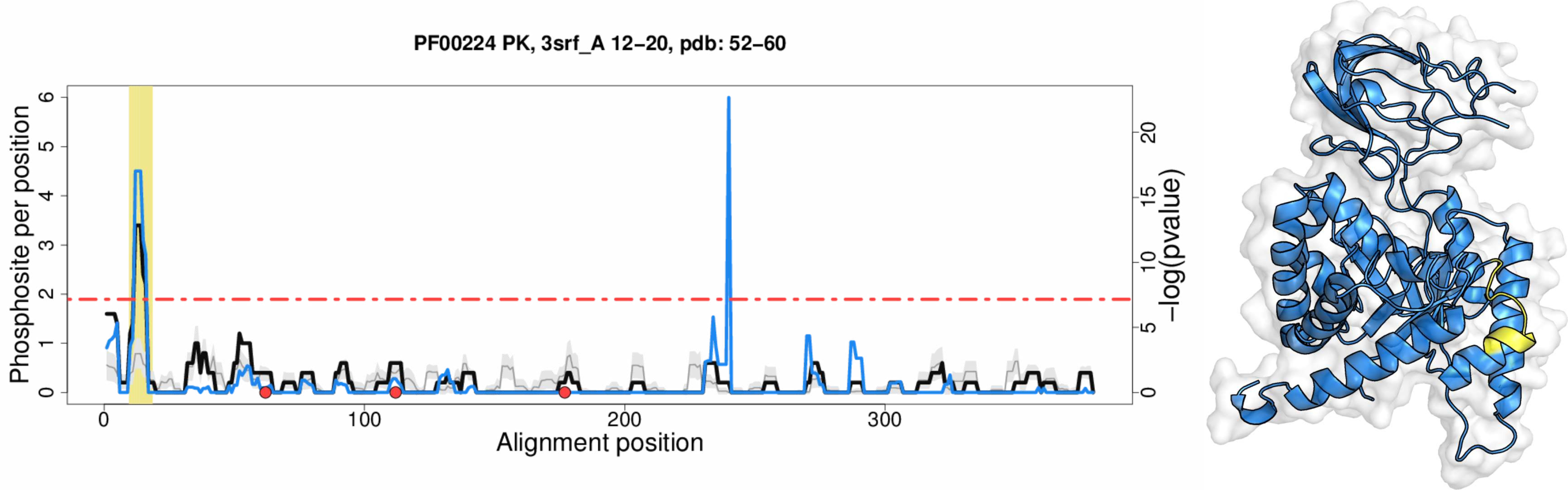
PF00224 PK, 3srf_A 12−20, pdb: 52−60
Phosphosite per position
−log(pvalue)
Alignment position
0
1
2
3
4
5
6
0
5
10
15
20
0
100
200
300

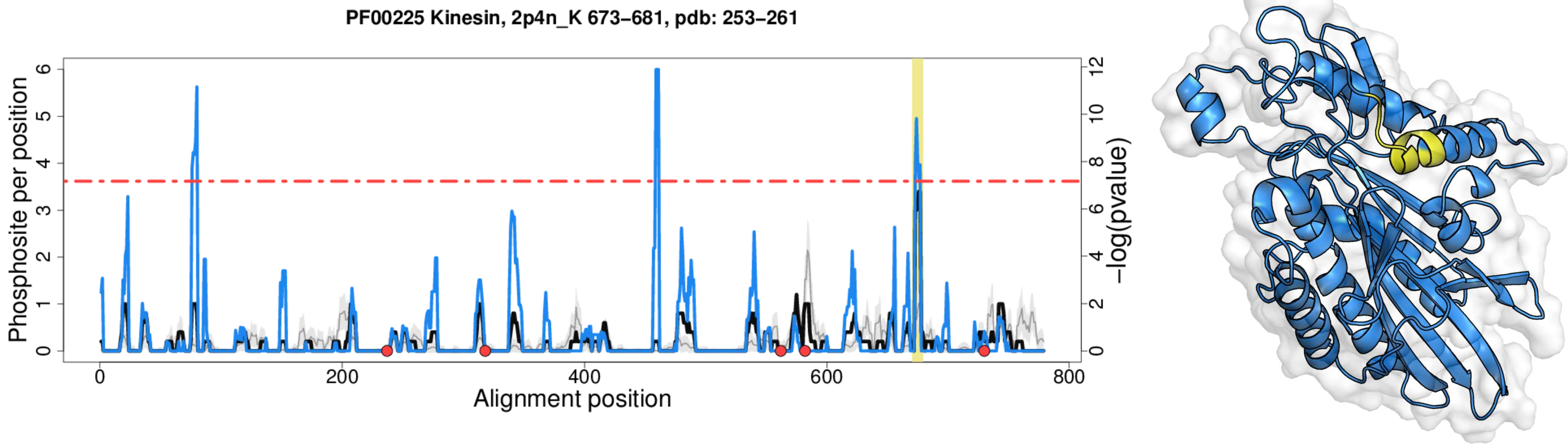

PF00225 Kinesin, 2p4n_K 673–681, pdb: 253–261
Phosphosite per position
0
1
2
3
4
5
6
Alignment position
0
200
400
600
800
−log(pvalue)
0
2
4
6
8
10
12

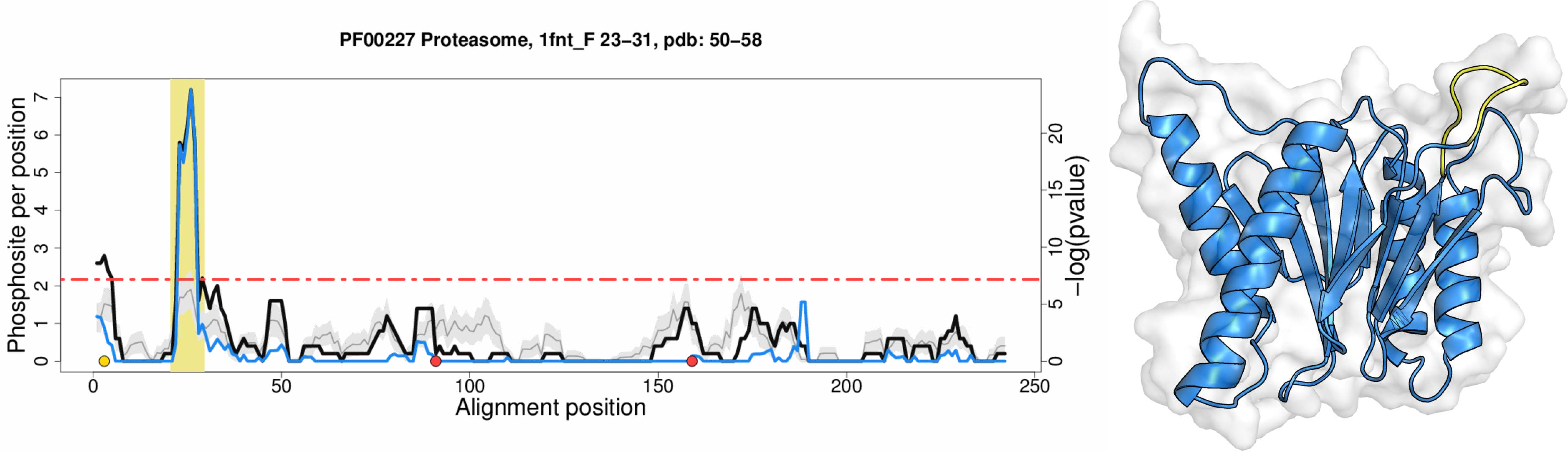

PF00227 Proteasome, 1fnt_F 23−31, pdb: 50−58
Phosphosite per position
−log(pvalue)
Alignment position
0
1
2
3
4
5
6
7
0
5
10
15
20
0
50
100
150
200
250

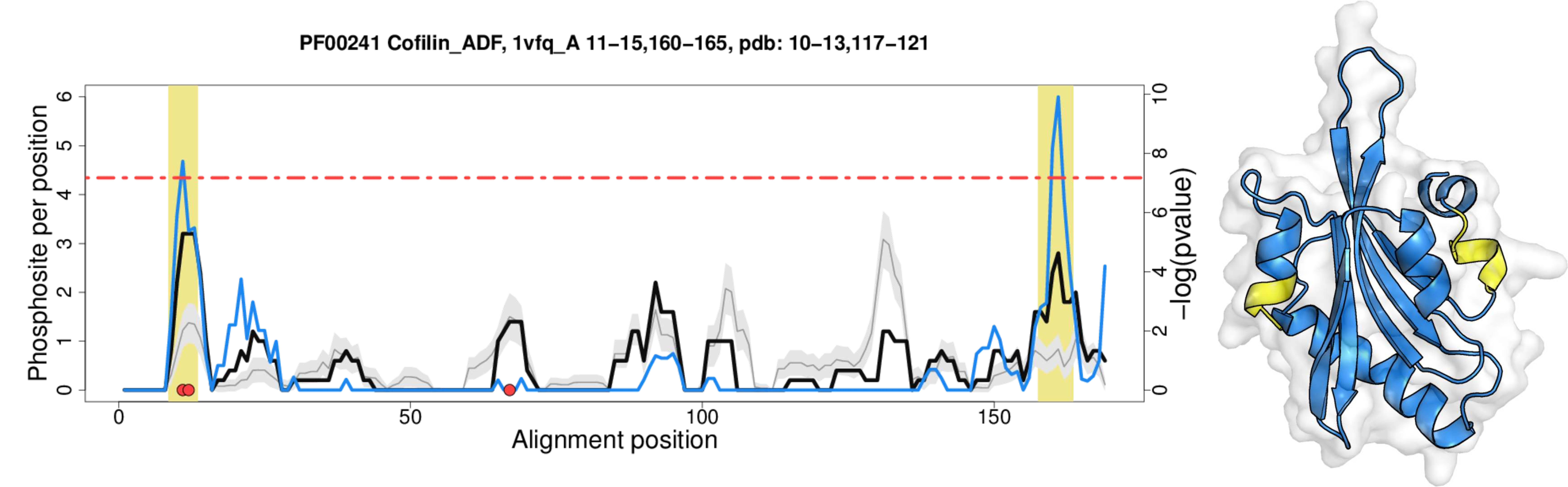

PF00241 Cofilin_ADF, 1vfq_A 11−15,160−165, pdb: 10−13,117−121
Phosphosite per position
−log(pvalue)
Alignment position
0
1
2
3
4
5
6
0
2
4
6
8
10
0
50
100
150

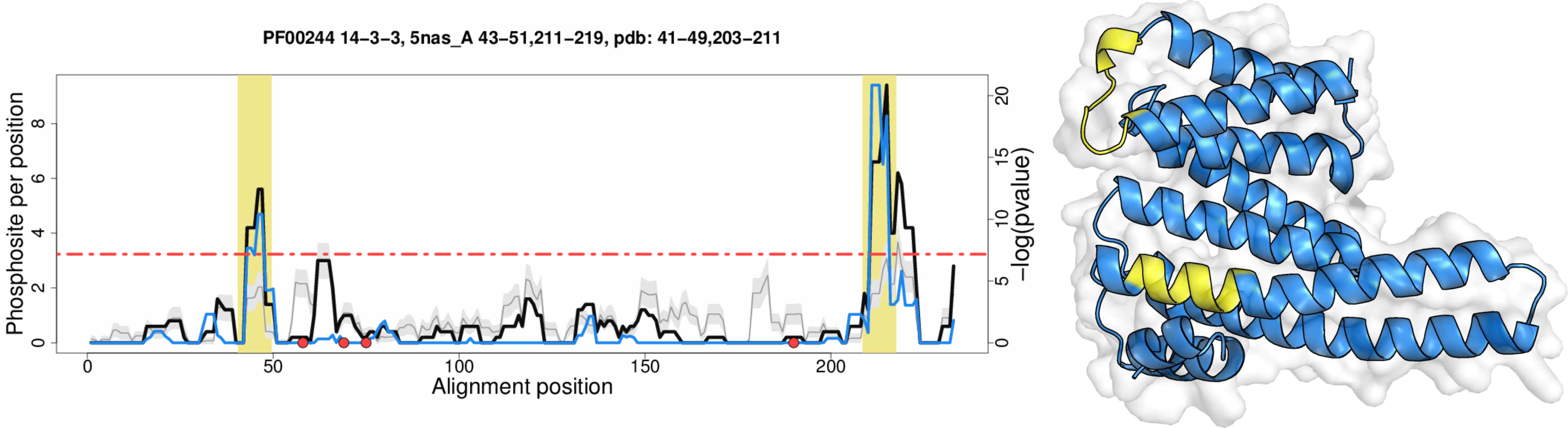

PF00244 14−3−3, 5nas_A 43−51,211−219, pdb: 41−49,203−211
Phosphosite per position
0
2
4
6
8
−log(pvalue)
0
5
10
15
20
0
50
100
150
200
Alignment position

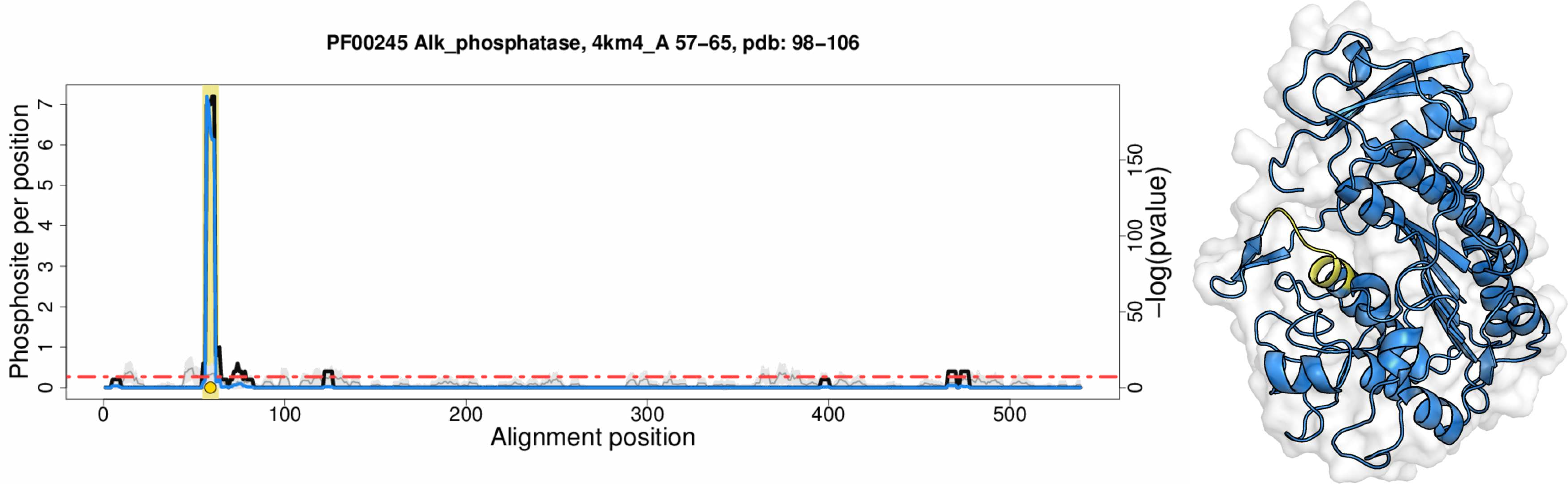
PF00245 Alk_phosphatase, 4km4_A 57−65, pdb: 98−106
Phosphosite per position
−log(pvalue)
Alignment position
0
1
2
3
4
5
6
7
0
50
100
150
0
100
200
300
400
500

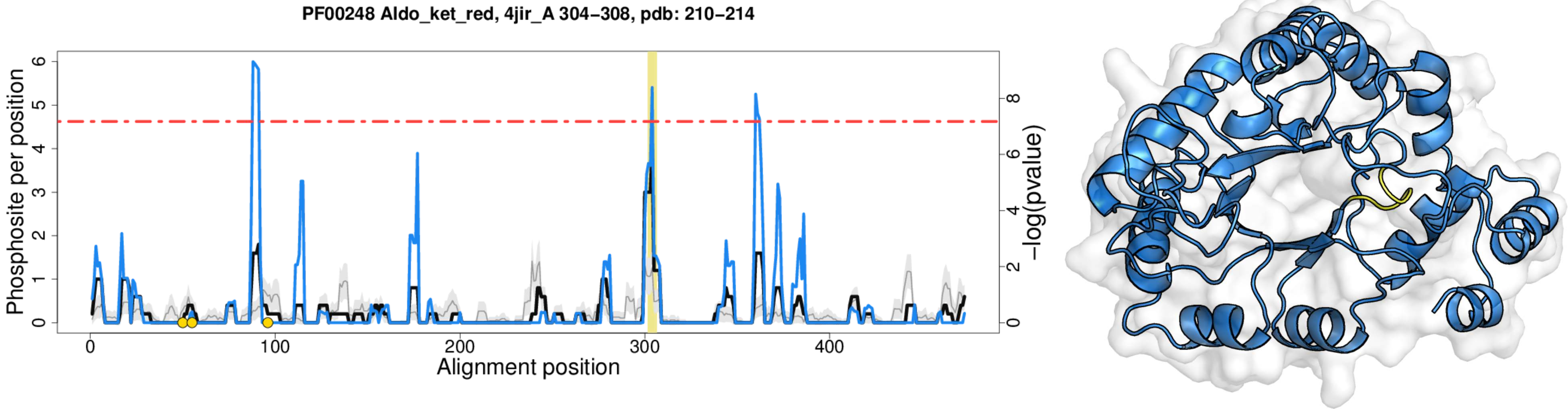

PF00248 Aldo_ket_red, 4jir_A 304−308, pdb: 210−214
Phosphosite per position
Alignment position
−log(pvalue)
0
1
2
3
4
5
6
0
100
200
300
400
0
2
4
6
8

PF00254 FKBP_C, 2l2s_A 46−51, pdb: 55−59

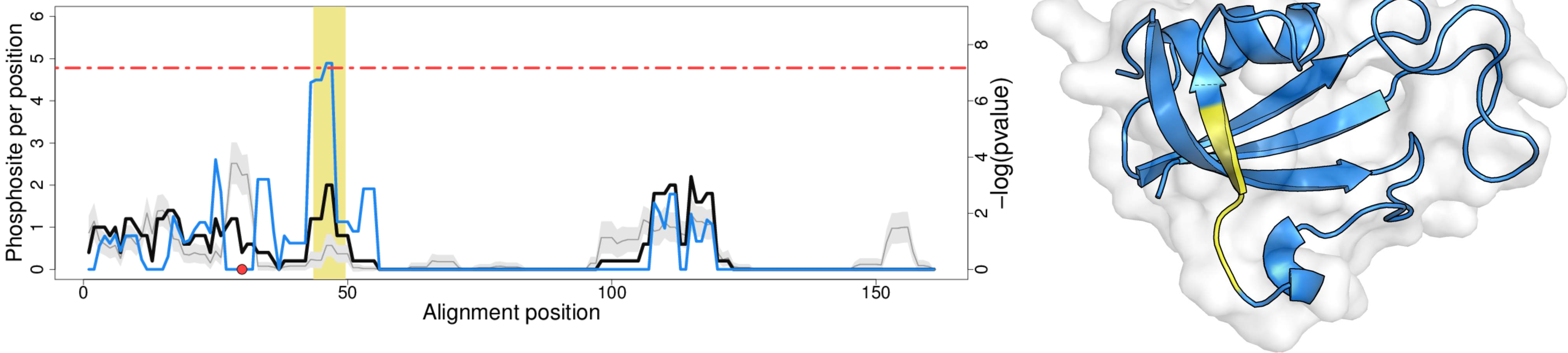

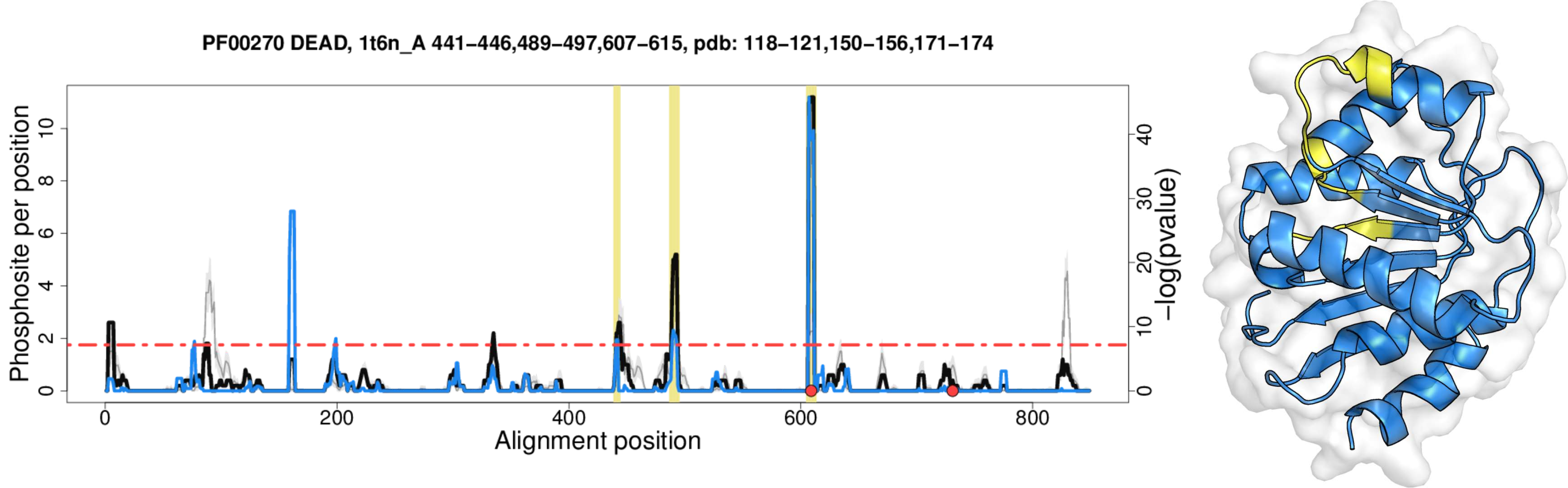

PF00270 DEAD, 1t6n_A 441–446,489–497,607–615, pdb: 118–121,150–156,171–174
Phosphosite per position
0
2
4
6
8
10
Alignment position
0
200
400
600
800
–log(pvalue)
0
10
20
30
40

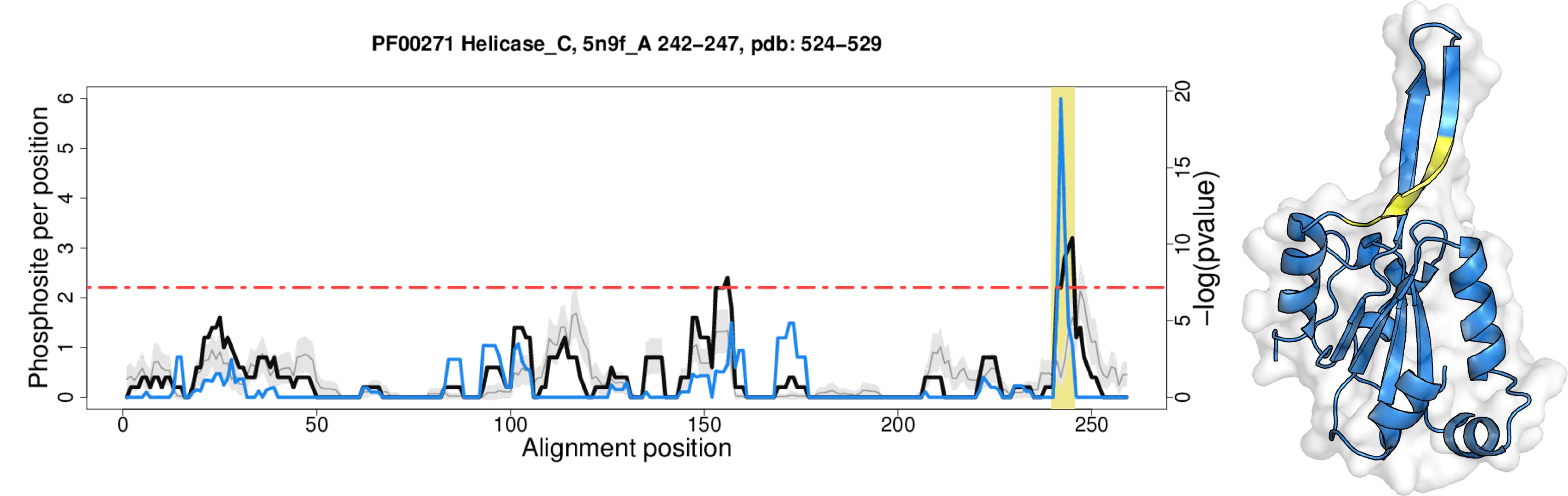
PF00271 Helicase_C, 5n9f_A 242–247, pdb: 524–529
Phosphosite per position
0
1
2
3
4
5
6
−log(pvalue)
0
5
10
15
20
Alignment position
0
50
100
150
200
250

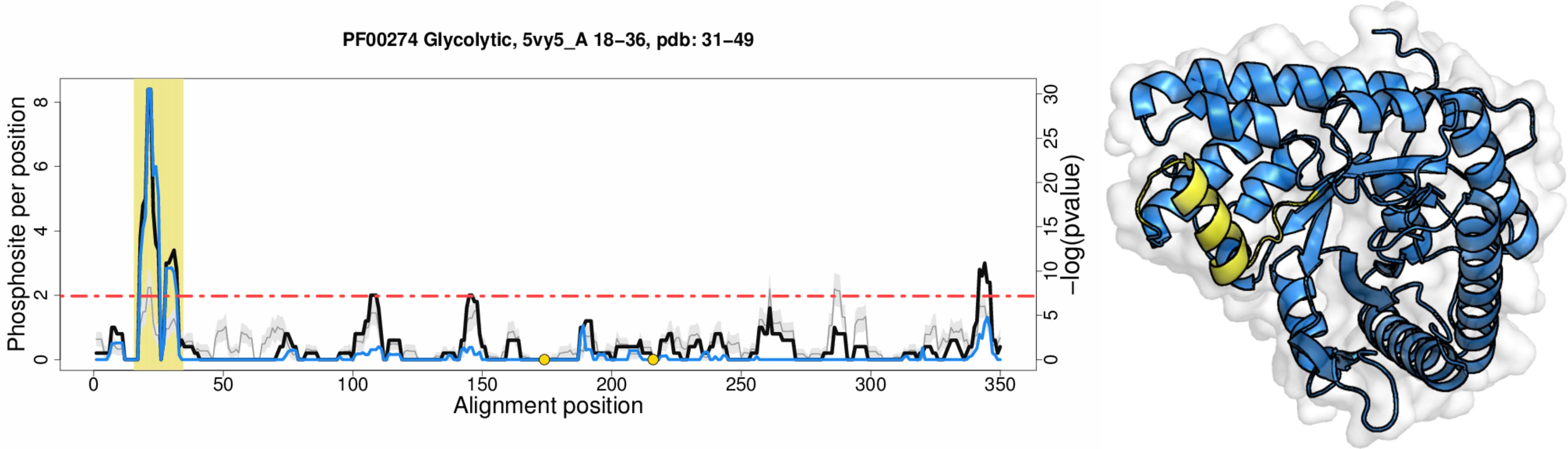
PF00274 Glycolytic, 5vy5_A 18-36, pdb: 31-49
Phosphosite per position
-log(pvalue)
Alignment position
0
2
4
6
8
0
5
10
15
20
25
30
0
50
100
150
200
250
300
350

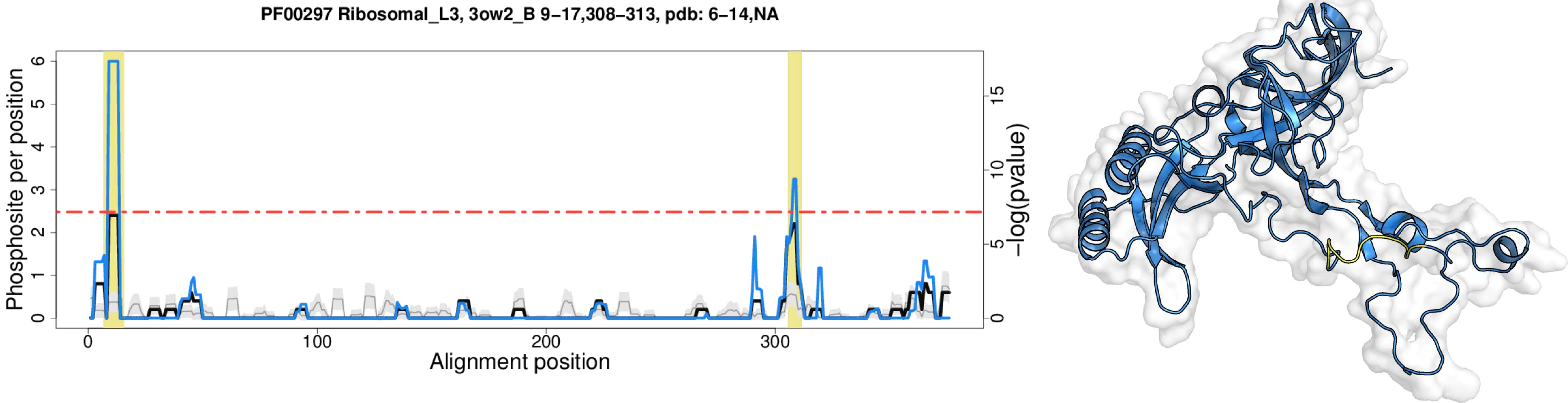

PF00297 Ribosomal_L3, 3ow2_B 9−17,308−313, pdb: 6−14,NA
Phosphosite per position
0
1
2
3
4
5
6
−log(pvalue)
0
5
10
15
0
100
200
300
Alignment position

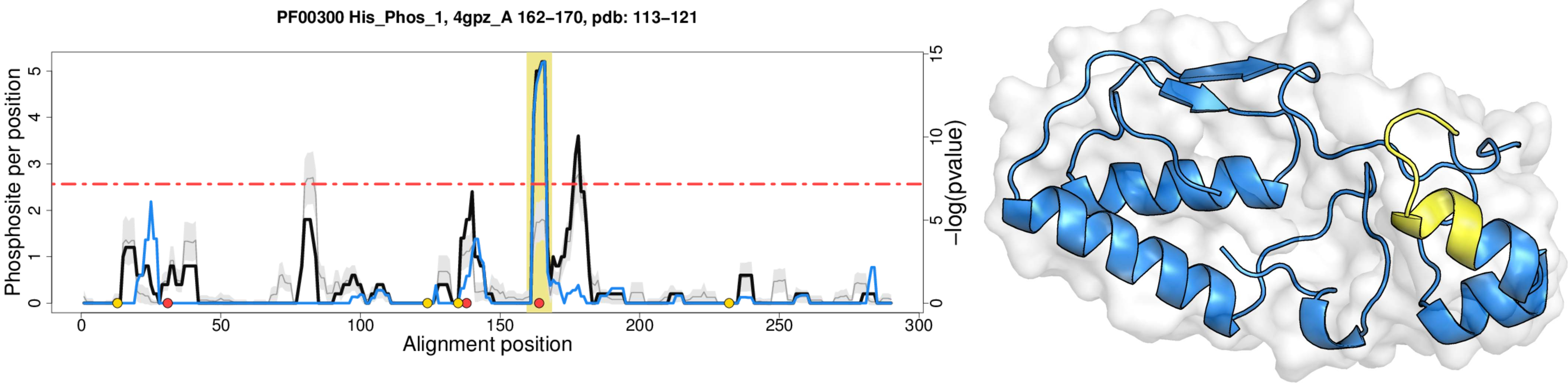
PF00300 His_Phos_1, 4gpz_A 162–170, pdb: 113–121
Phosphosite per position
0
1
2
3
4
5
Alignment position
0
50
100
150
200
250
300
–log(pvalue)
0
5
10
15

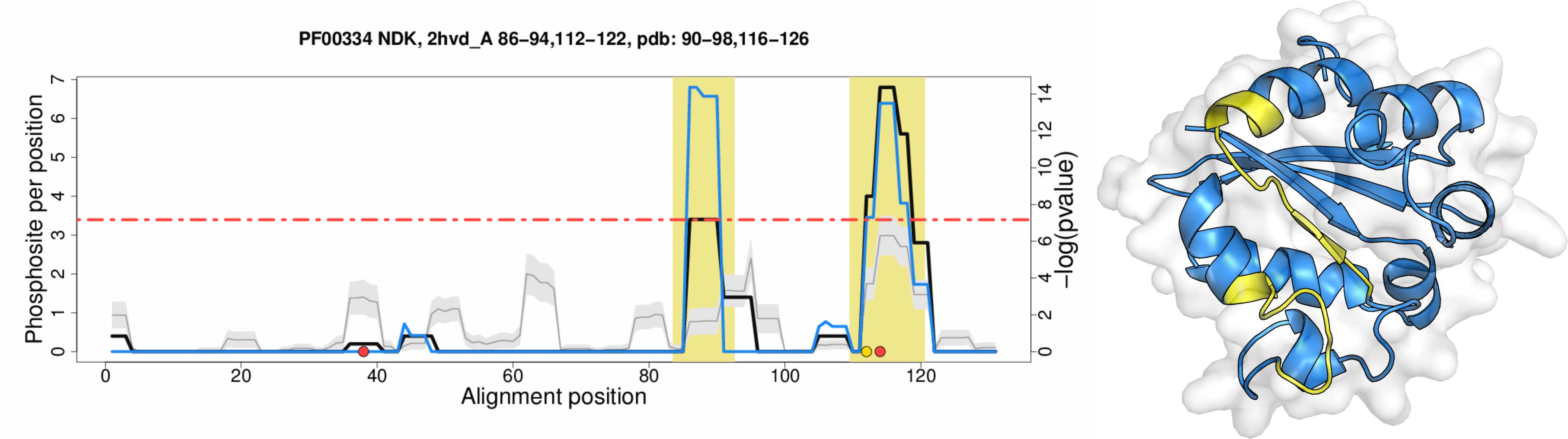
PF00334 NDK, 2hvd_A 86−94,112−122, pdb: 90−98,116−126
Phosphosite per position
−log(pvalue)
Alignment position
0
20
40
60
80
100
120
0
1
2
3
4
5
6
7
0
2
4
6
8
10
12
14

PF00339 Arrestin_N, 4jqi_A 106−111,129−134, pdb: NA,79−84

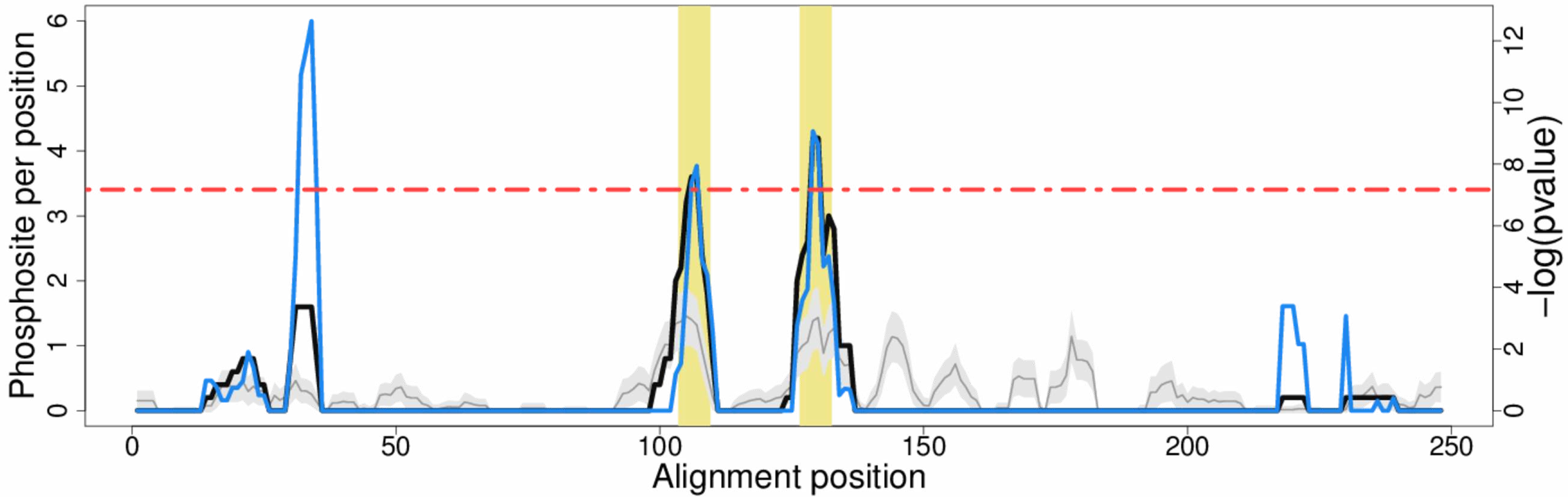

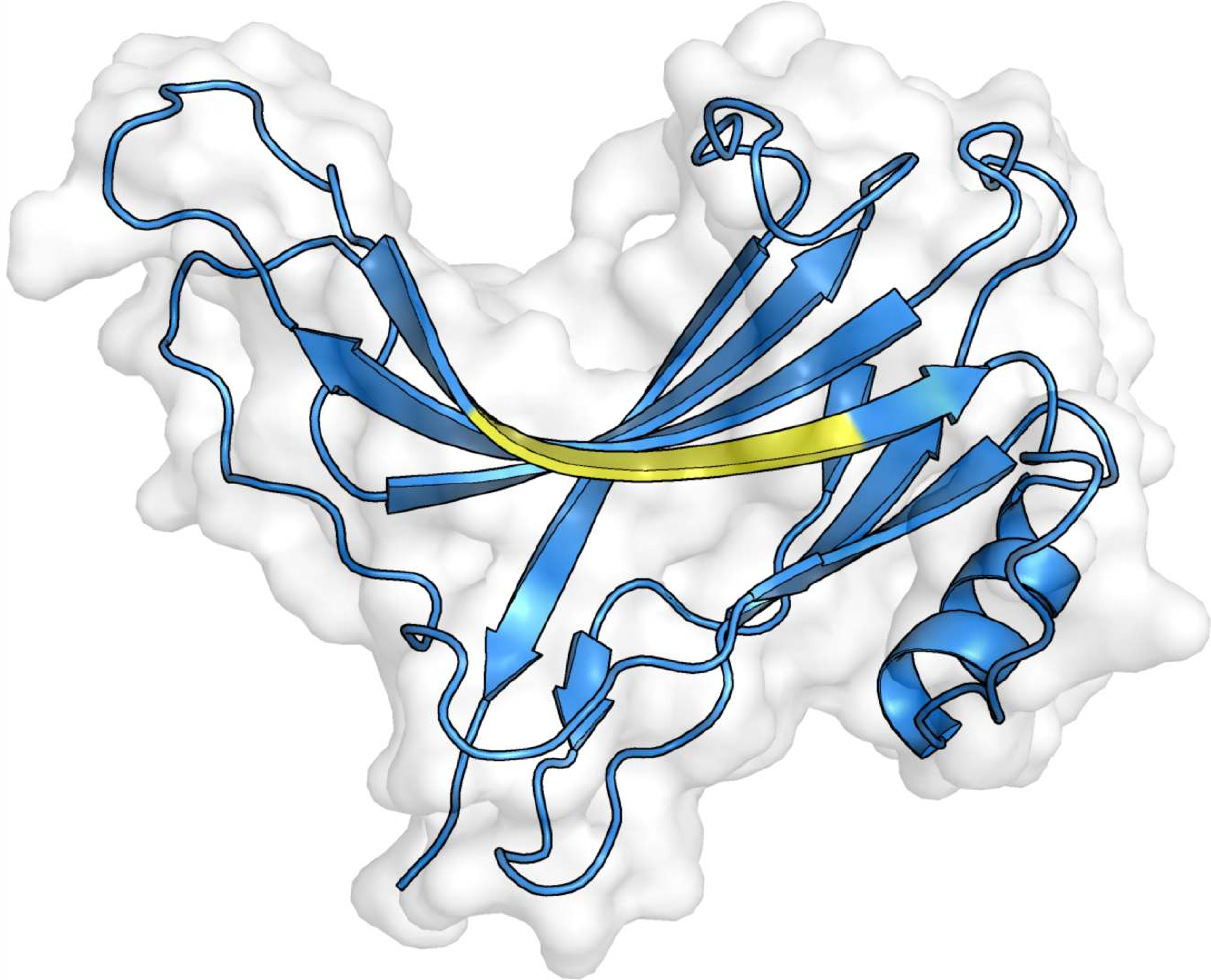

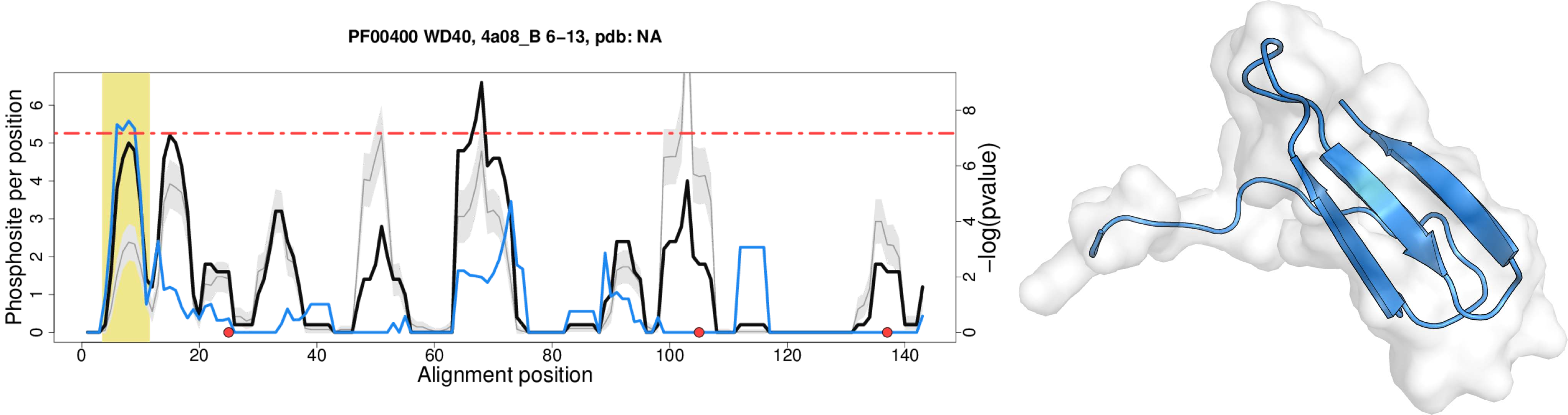
PF00400 WD40, 4a08_B 6−13, pdb: NA
Phosphosite per position
−log(pvalue)
Alignment position
0
1
2
3
4
5
6
0
2
4
6
8
0
20
40
60
80
100
120
140

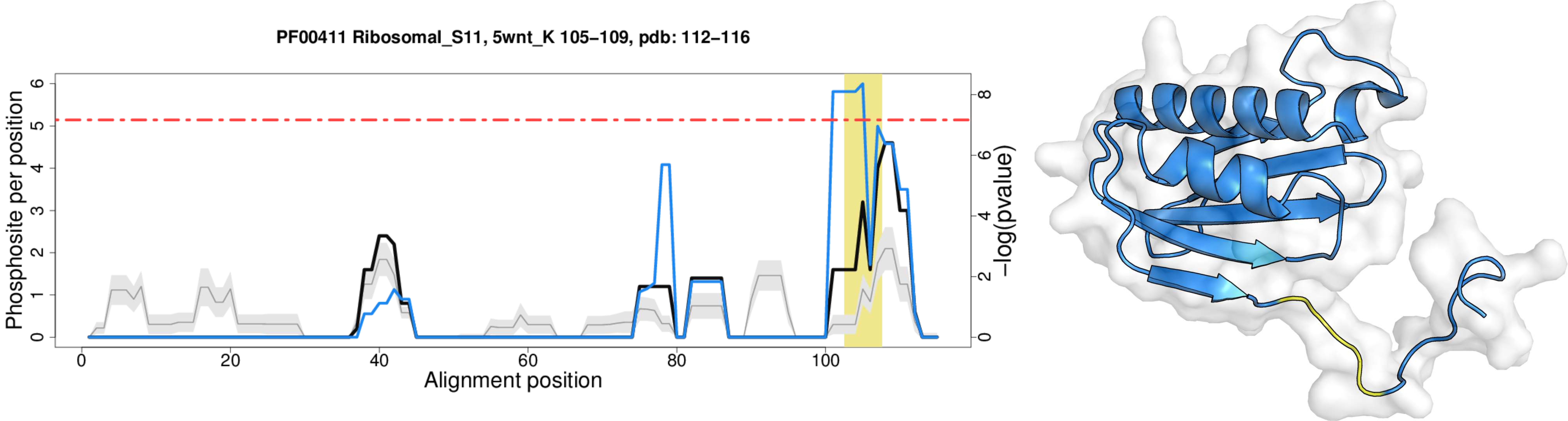
PF00411 Ribosomal_S11, 5wnt_K 105–109, pdb: 112–116
Phosphosite per position
0
1
2
3
4
5
6
−log(pvalue)
0
2
4
6
8
0
20
40
60
80
100
Alignment position

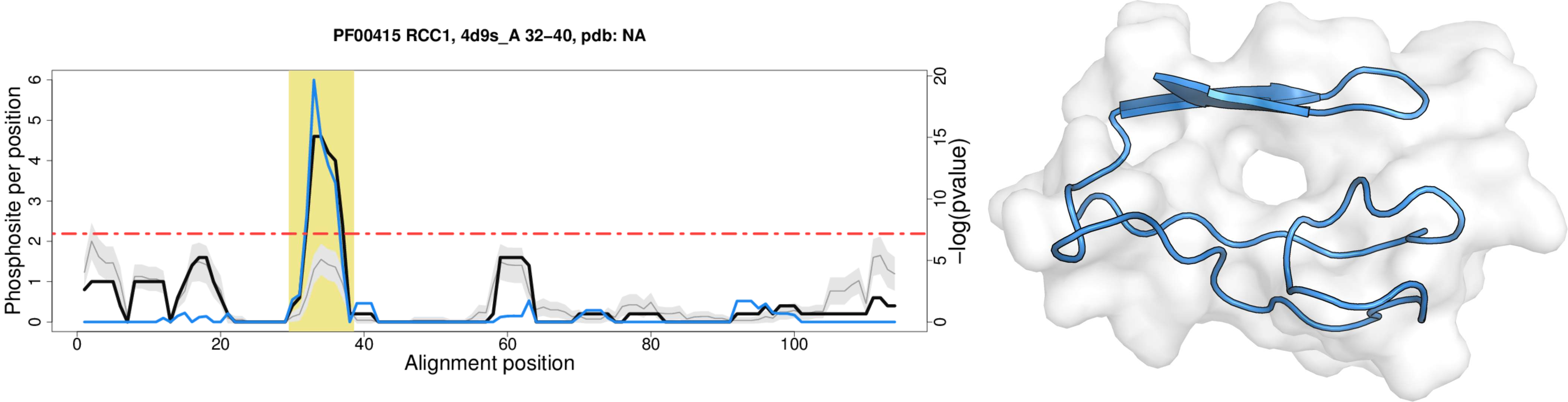
PF00415 RCC1, 4d9s_A 32–40, pdb: NA
Phosphosite per position
−log(pvalue)
Alignment position
0
1
2
3
4
5
6
0
5
10
15
20
0
20
40
60
80
100

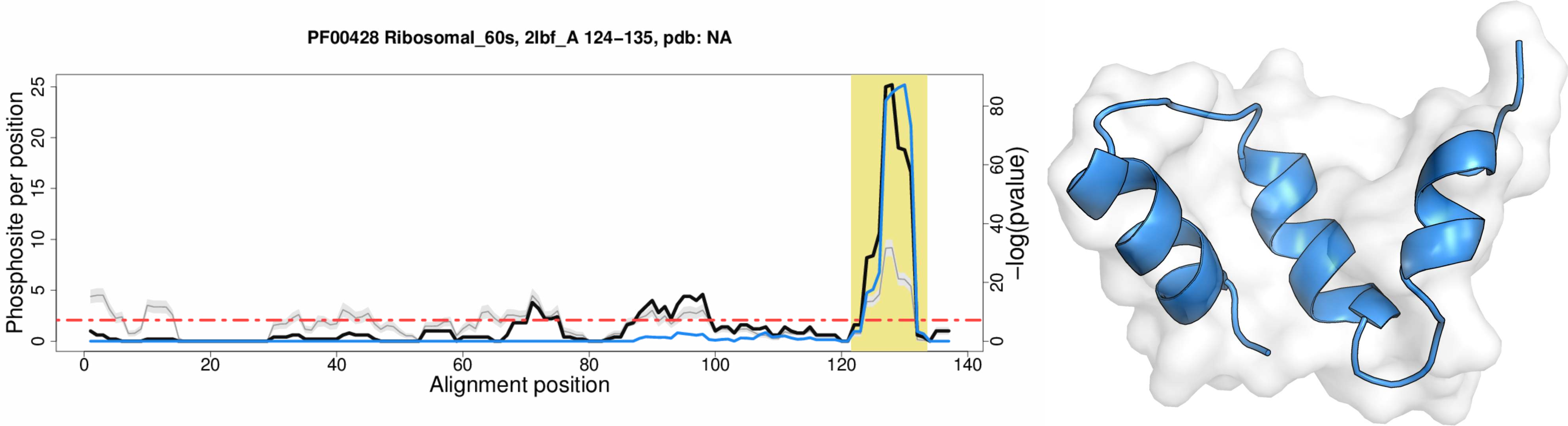
PF00428 Ribosomal_60s, 2lbf_A 124−135, pdb: NA
Phosphosite per position
−log(pvalue)
Alignment position
0
5
10
15
20
25
0
20
40
60
80
0
20
40
60
80
100
120
140

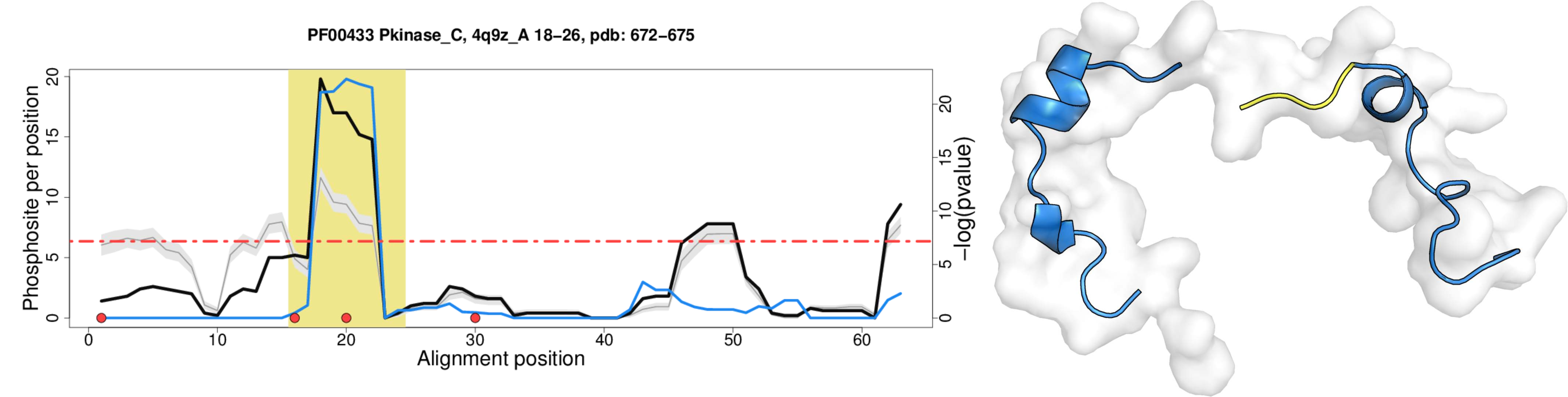

PF00433 Pkinase_C, 4q9z_A 18–26, pdb: 672–675
Phosphosite per position
0
5
10
15
20
Alignment position
0
10
20
30
40
50
60
–log(pvalue)
0
5
10
15
20

PF00443 UCH, 5j7t_A 1064–1072,1176–1183,2978–2987,3021–3025,3094–3098,3239–3243, pdb: NA,NA,NA,NA,NA,NA

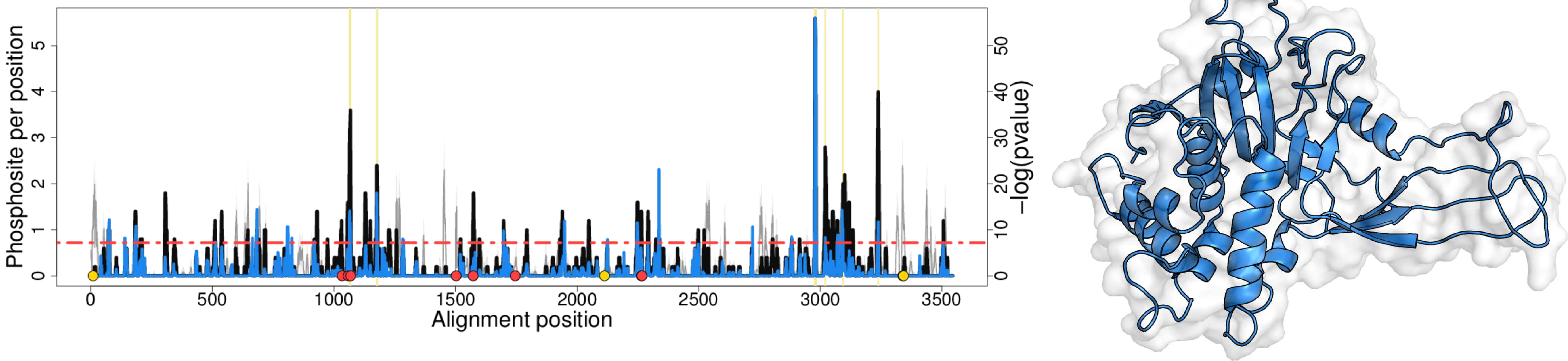

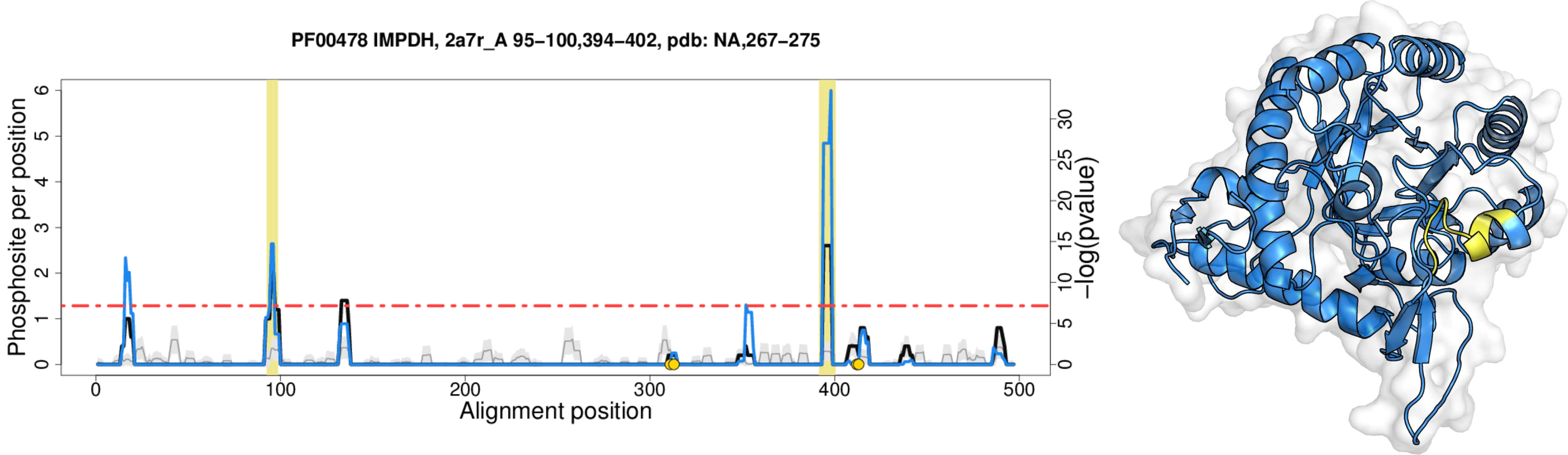
PF00478 IMPDH, 2a7r_A 95–100,394–402, pdb: NA,267–275
Phosphosite per position
0
1
2
3
4
5
6
Alignment position
0
100
200
300
400
500
−log(pvalue)
0
5
10
15
20
25
30

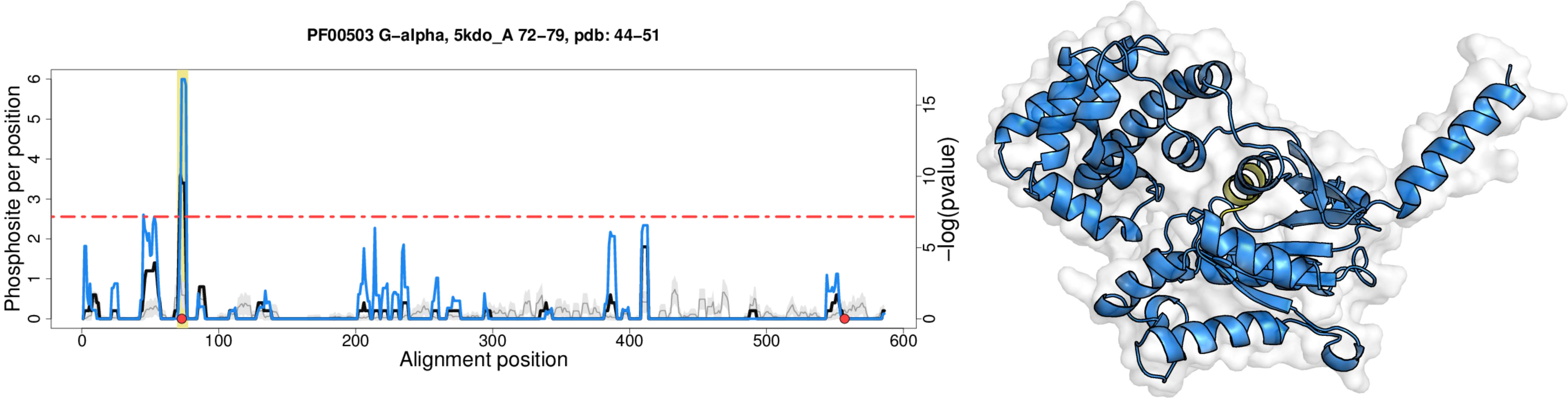
PF00503 G−alpha, 5kdo_A 72−79, pdb: 44−51
Phosphosite per position
−log(pvalue)
Alignment position
0
1
2
3
4
5
6
0
5
10
15
0
100
200
300
400
500
600

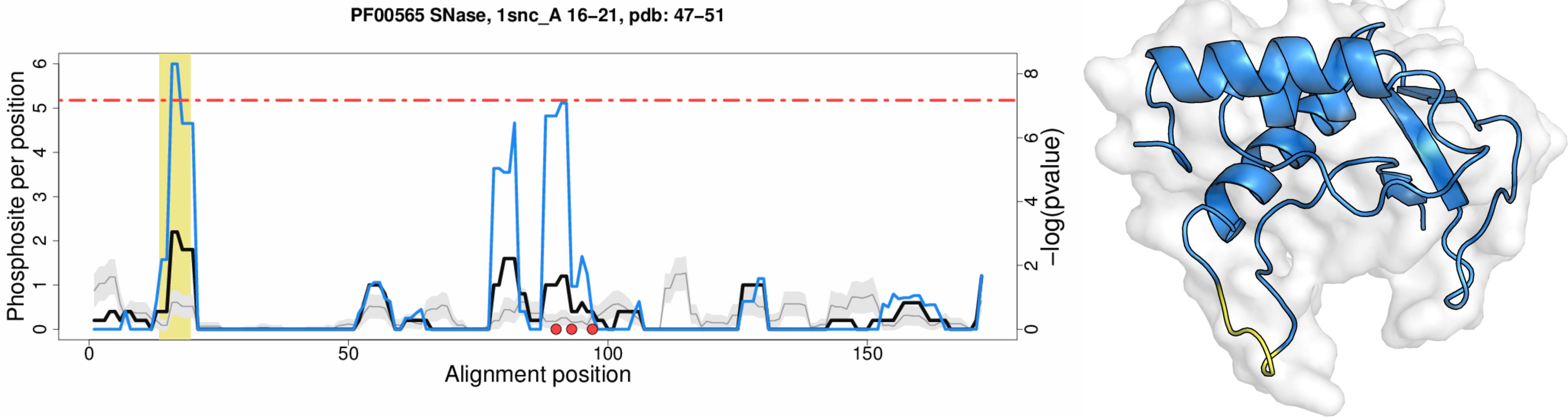

PF00565 SNase, 1snc_A 16−21, pdb: 47−51
Phosphosite per position
−log(pvalue)
Alignment position
0
50
100
150
0
1
2
3
4
5
6
0
2
4
6
8

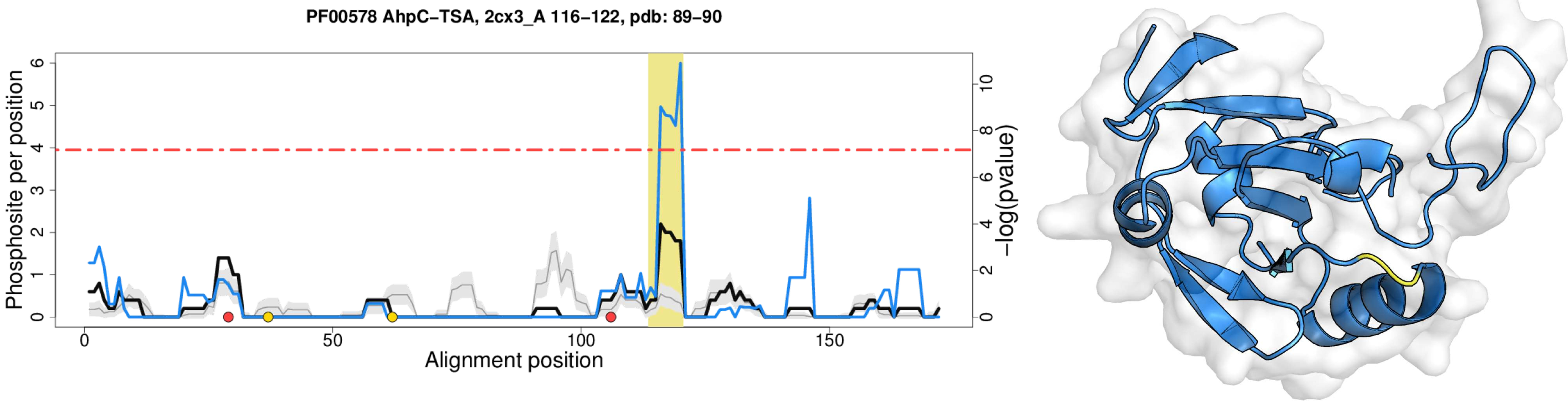

PF00578 AhpC−TSA, 2cx3_A 116−122, pdb: 89−90
Phosphosite per position
0
1
2
3
4
5
6
−log(pvalue)
0
2
4
6
8
10
0
50
100
150
Alignment position

PF00579 tRNA–synt_1b, 3n2y_A 256–264, pdb: 214–222

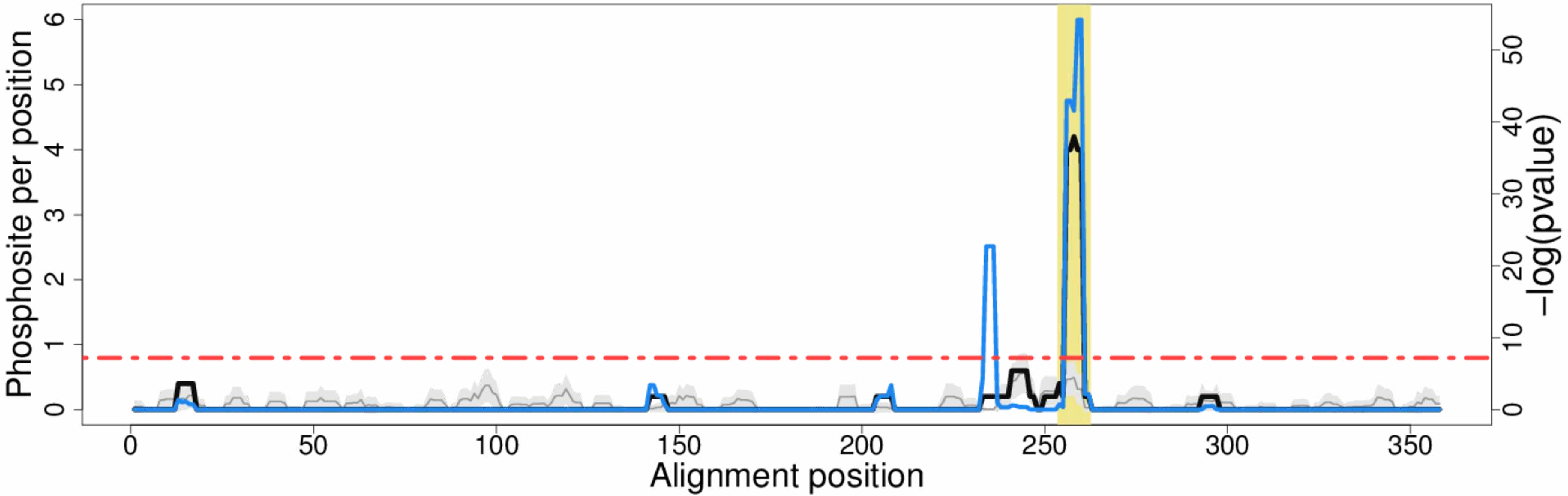

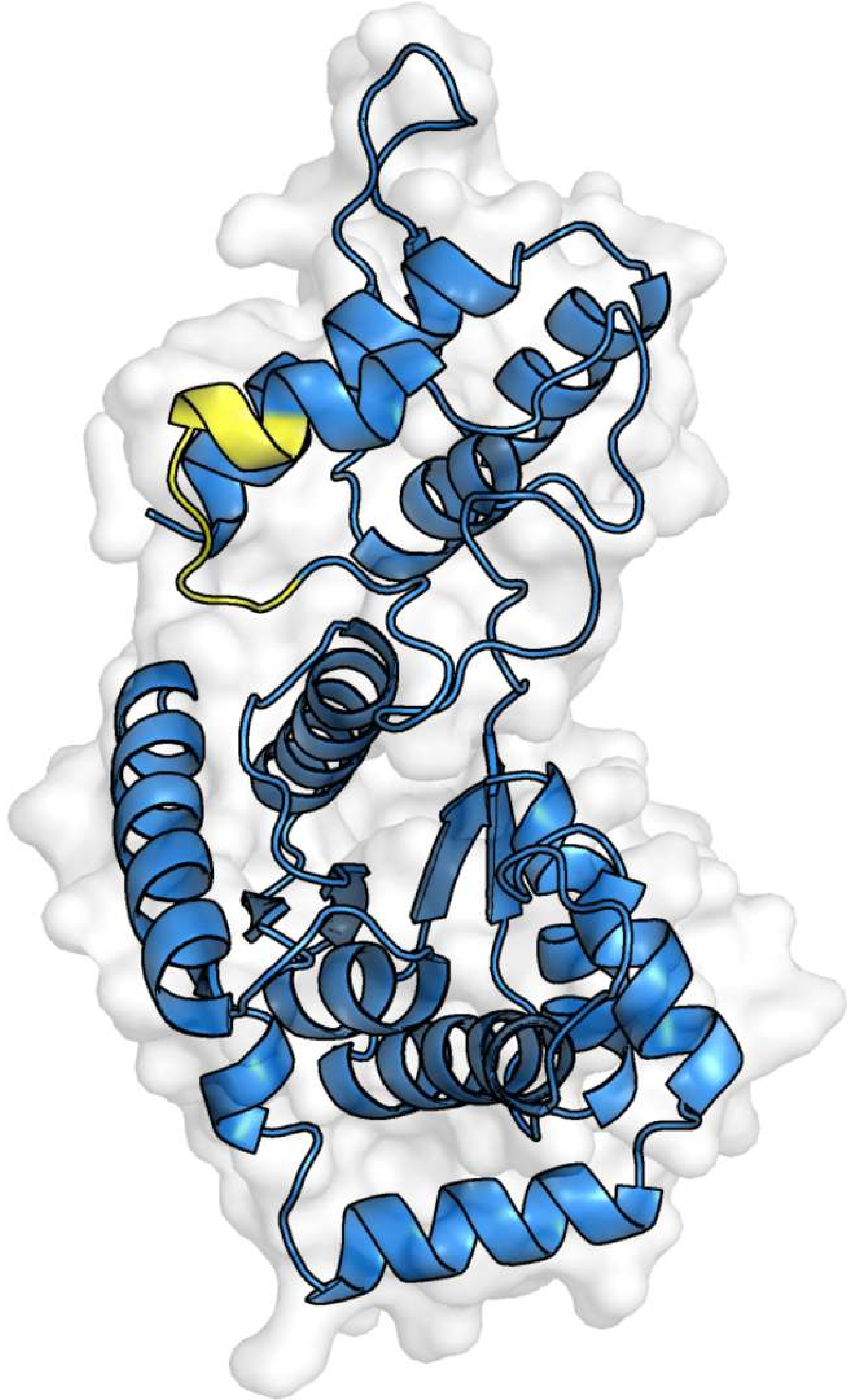

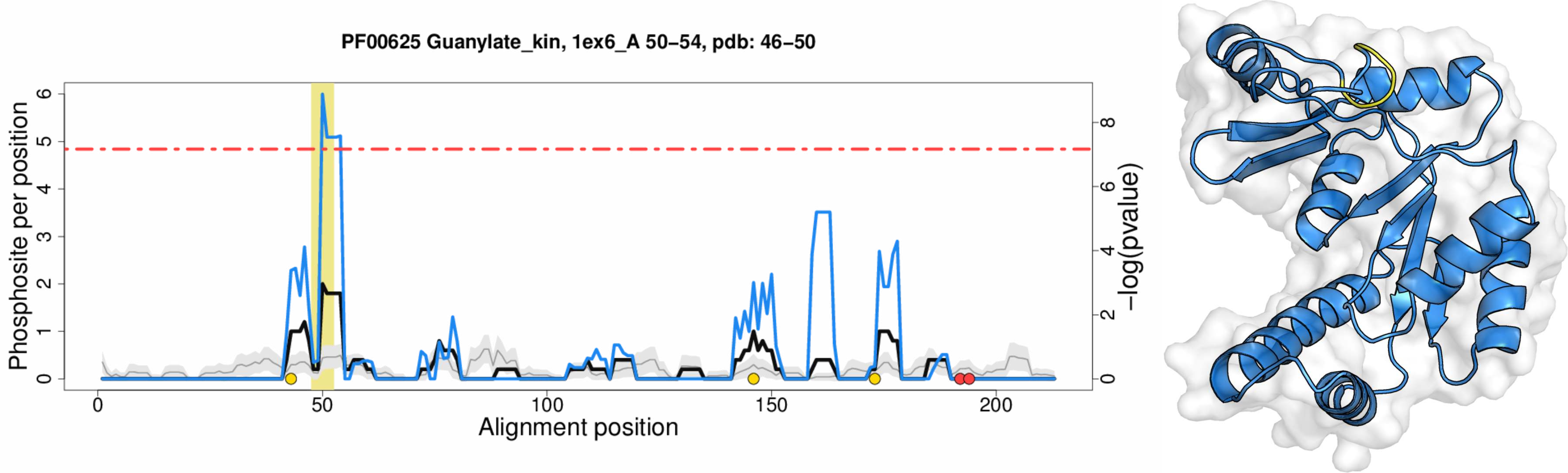
PF00625 Guanylate_kin, 1ex6_A 50−54, pdb: 46−50
Phosphosite per position
0
1
2
3
4
5
6
−log(pvalue)
0
2
4
6
8
Alignment position
0
50
100
150
200

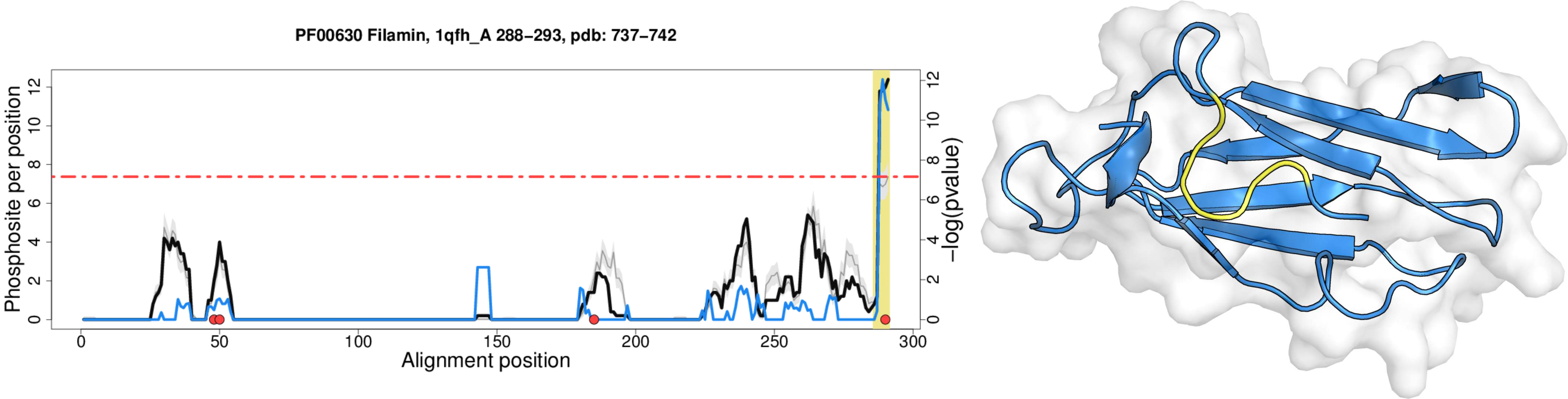
PF00630 Filamin, 1qfh_A 288–293, pdb: 737–742
Phosphosite per position
–log(pvalue)
Alignment position
0
50
100
150
200
250
300
0
2
4
6
8
10
12

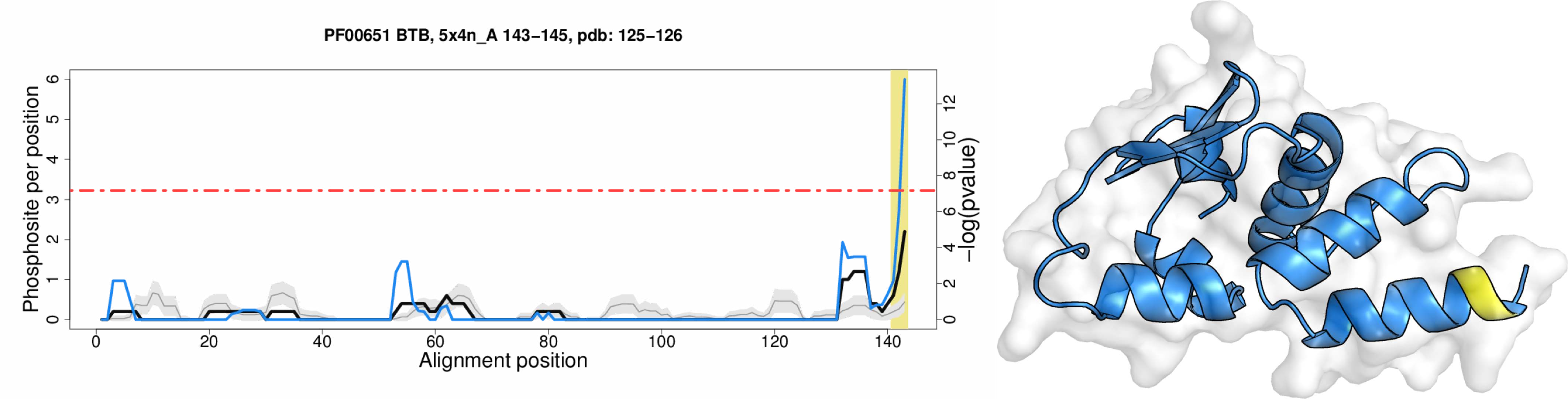

PF00651 BTB, 5x4n_A 143−145, pdb: 125−126
Phosphosite per position
Alignment position
−log(pvalue)

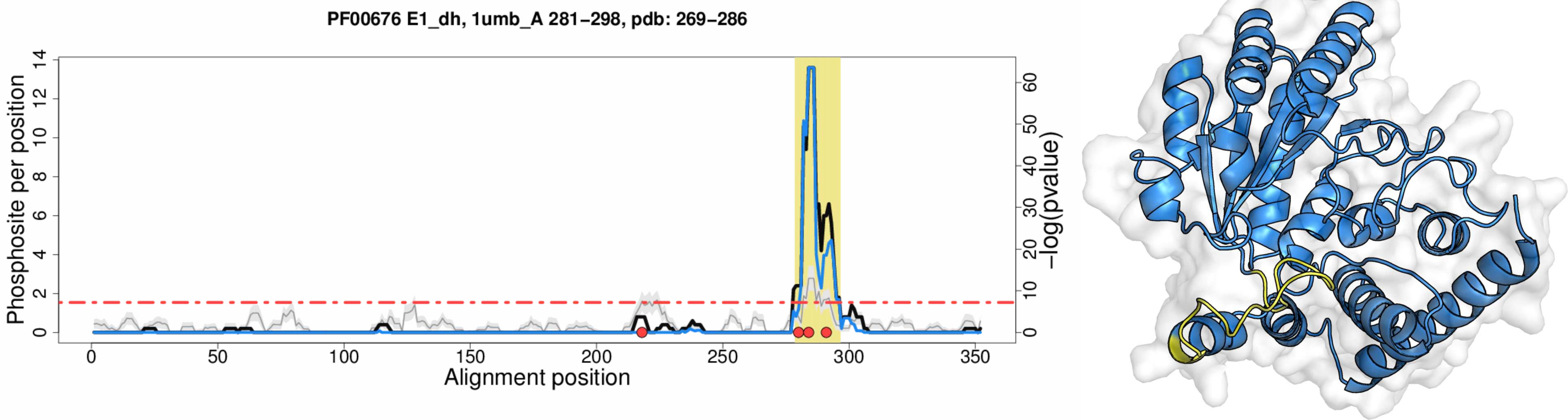

PF00676 E1_dh, 1umb_A 281−298, pdb: 269−286
Phosphosite per position
0
2
4
6
8
10
12
14
−log(pvalue)
0
10
20
30
40
50
60
Alignment position
0
50
100
150
200
250
300
350

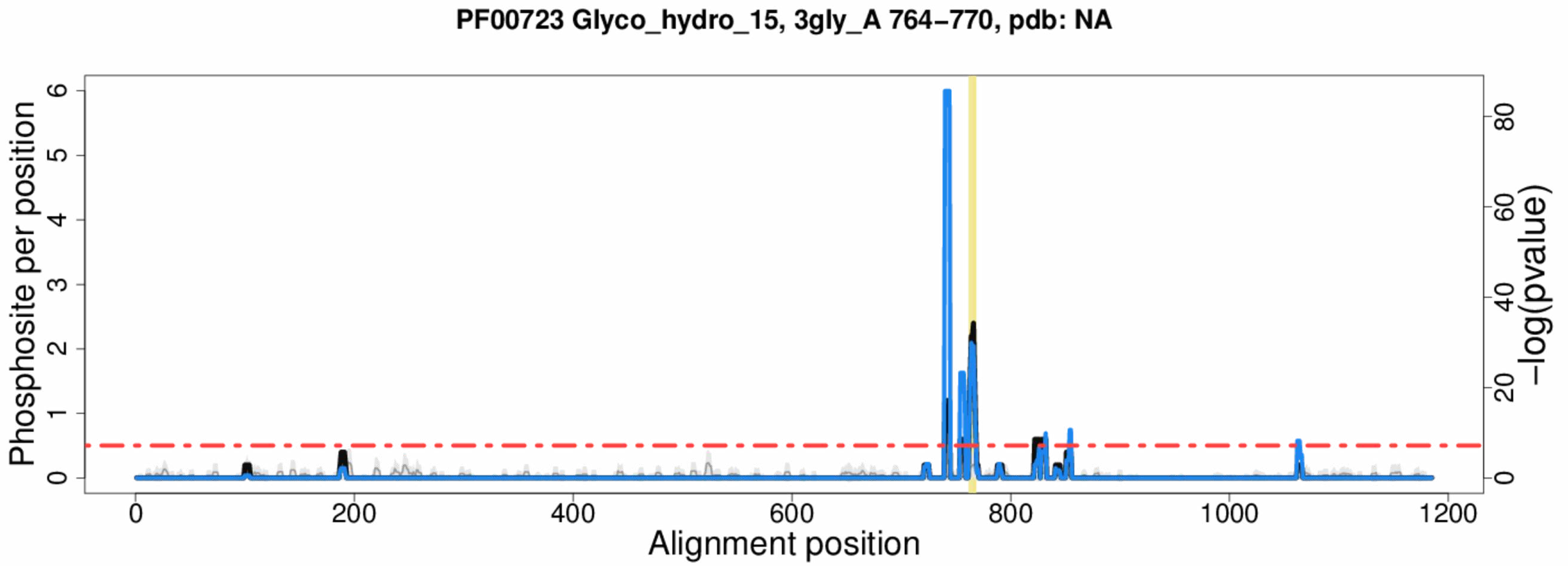
PF00723 Glyco_hydro_15, 3gly_A 764−770, pdb: NA
Phosphosite per position
0
1
2
3
4
5
6
−log(pvalue)
0
20
40
60
80
Alignment position
0
200
400
600
800
1000
1200

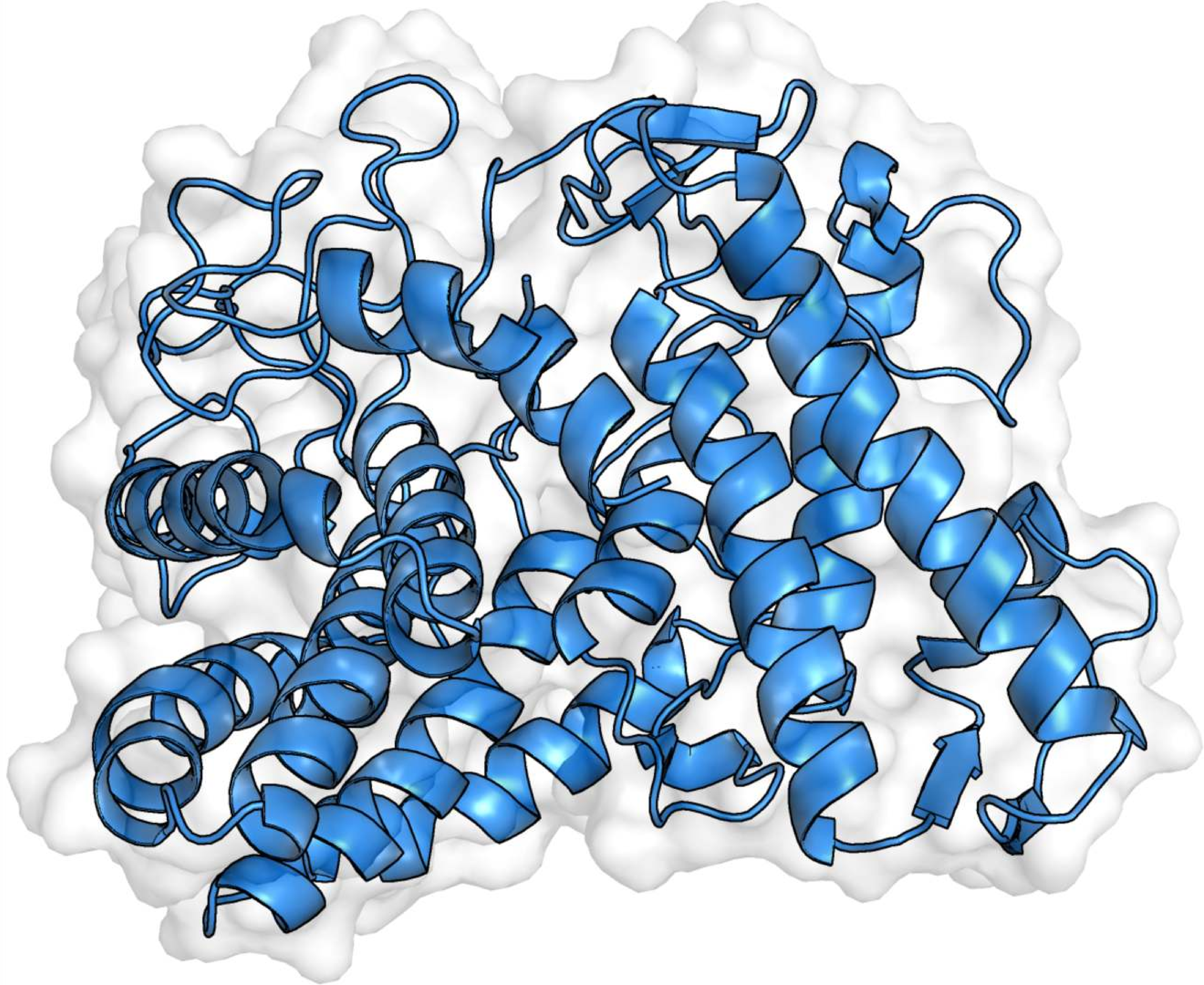

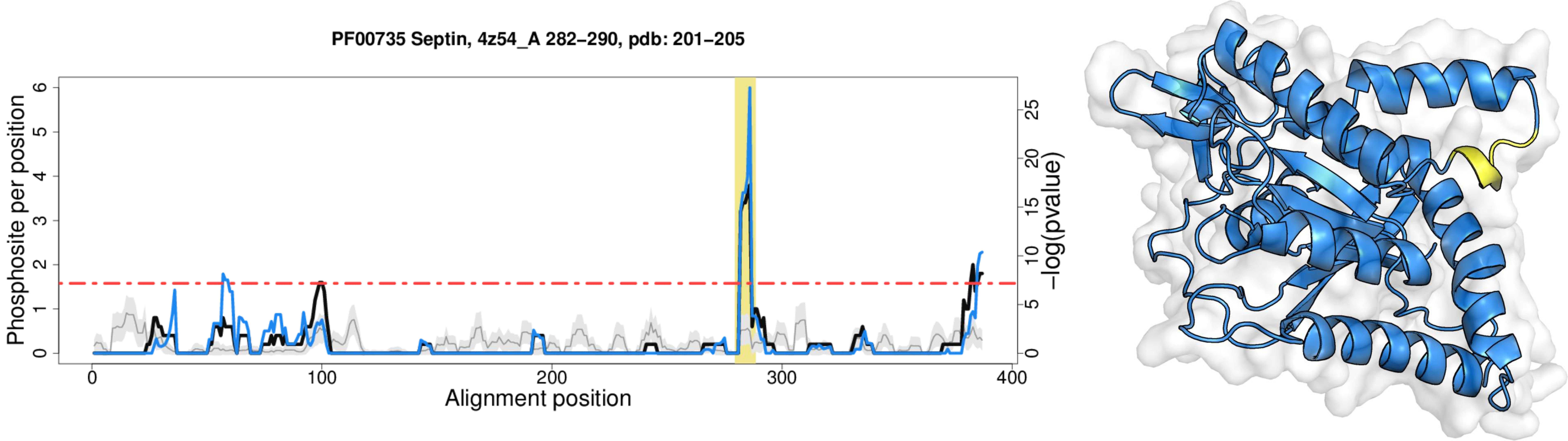
PF00735 Septin, 4z54_A 282–290, pdb: 201–205
Phosphosite per position
0
1
2
3
4
5
6
−log(pvalue)
0
5
10
15
20
25
Alignment position
0
100
200
300
400

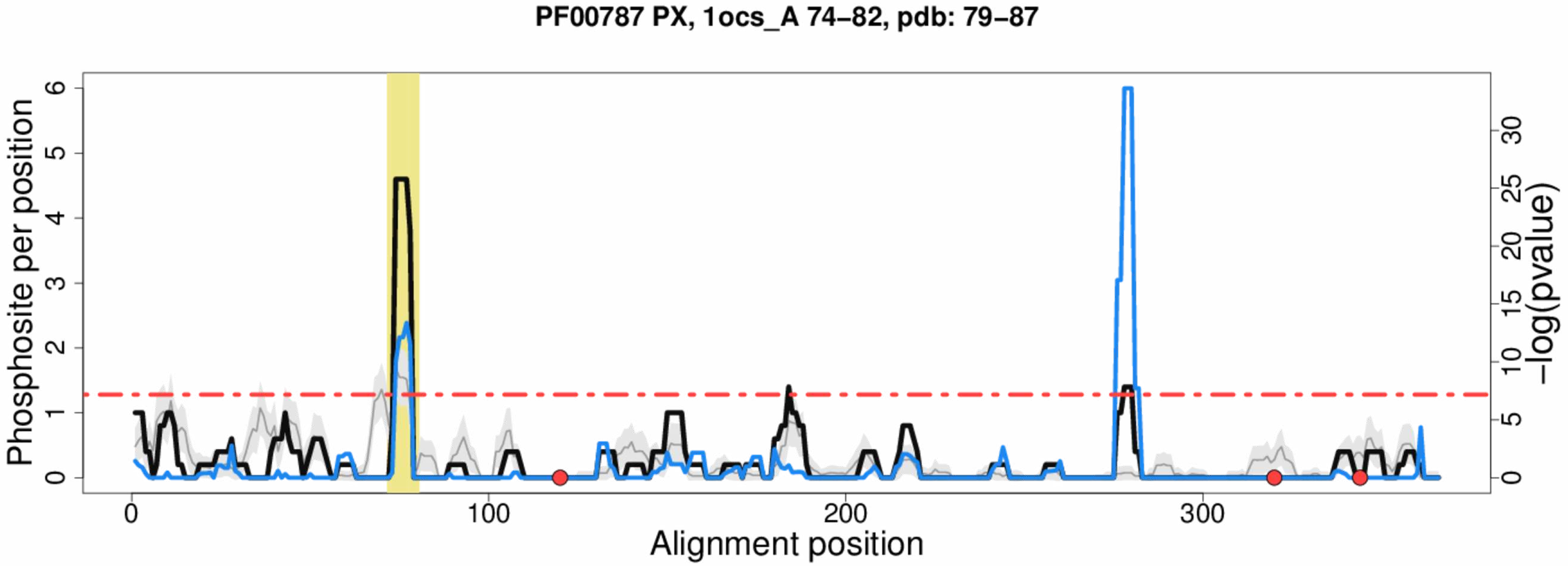

PF00787 PX, 1ocs_A 74−82, pdb: 79−87
Phosphosite per position
Alignment position
−log(pvalue)

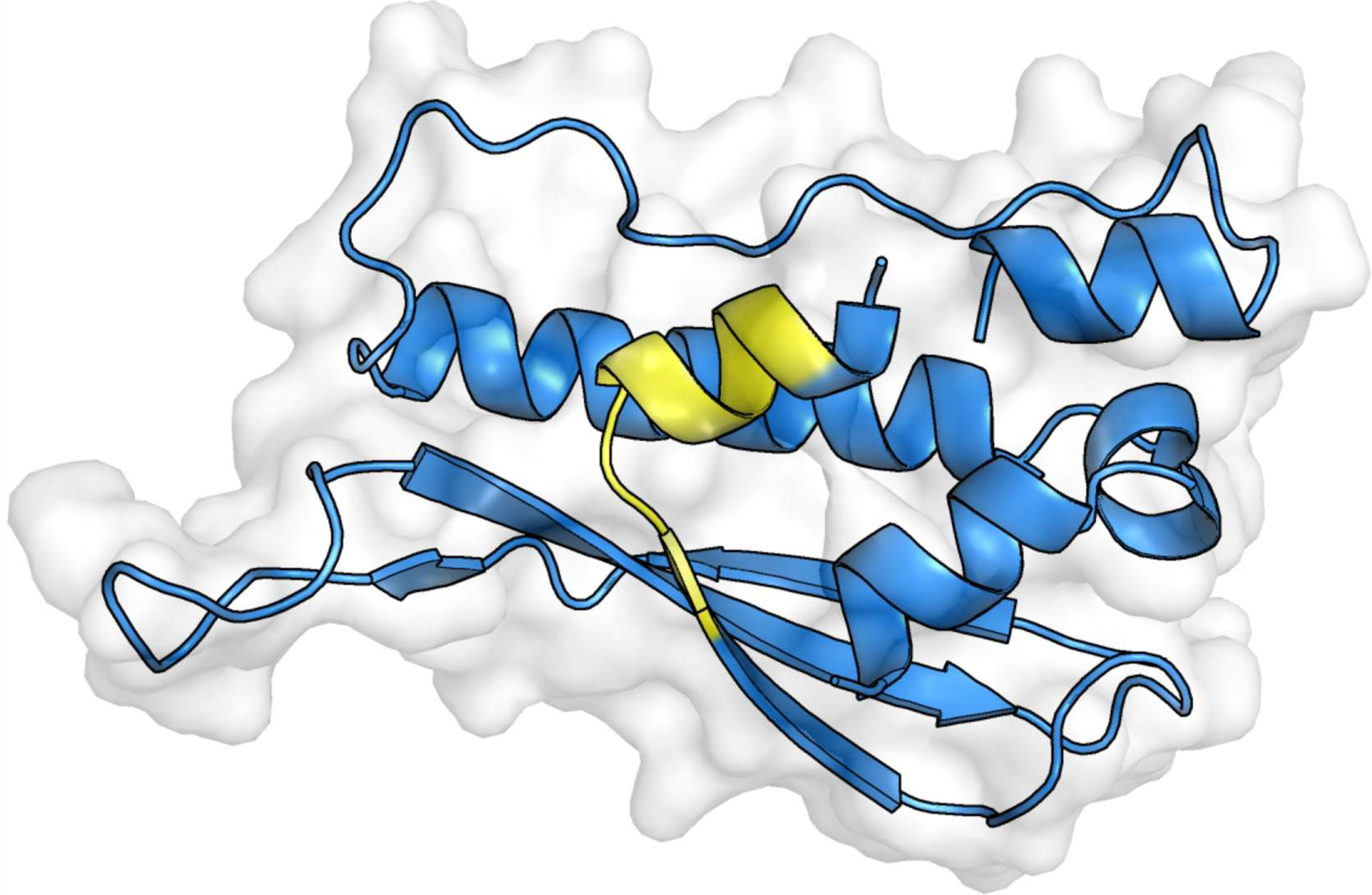

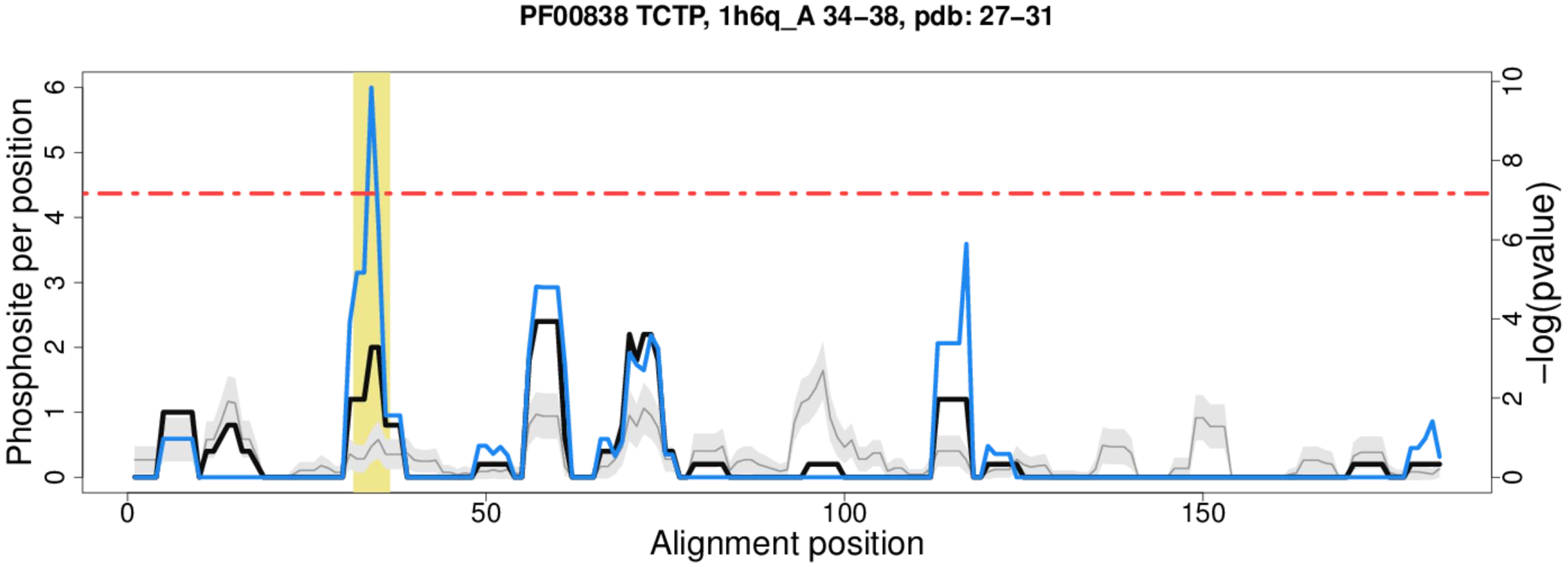

PF00838 TCTP, 1h6q_A 34−38, pdb: 27−31
Phosphosite per position
−log(pvalue)
Alignment position

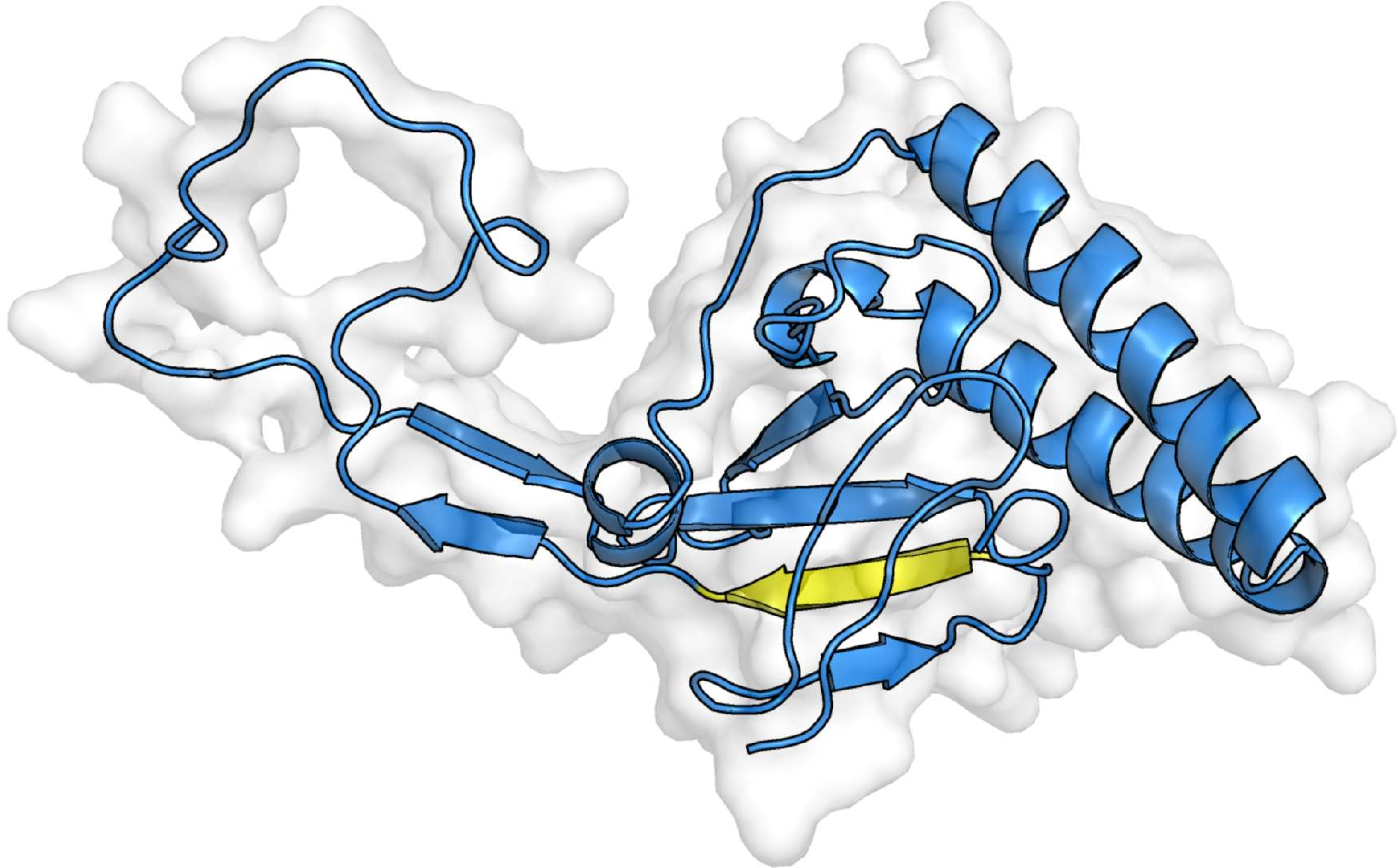

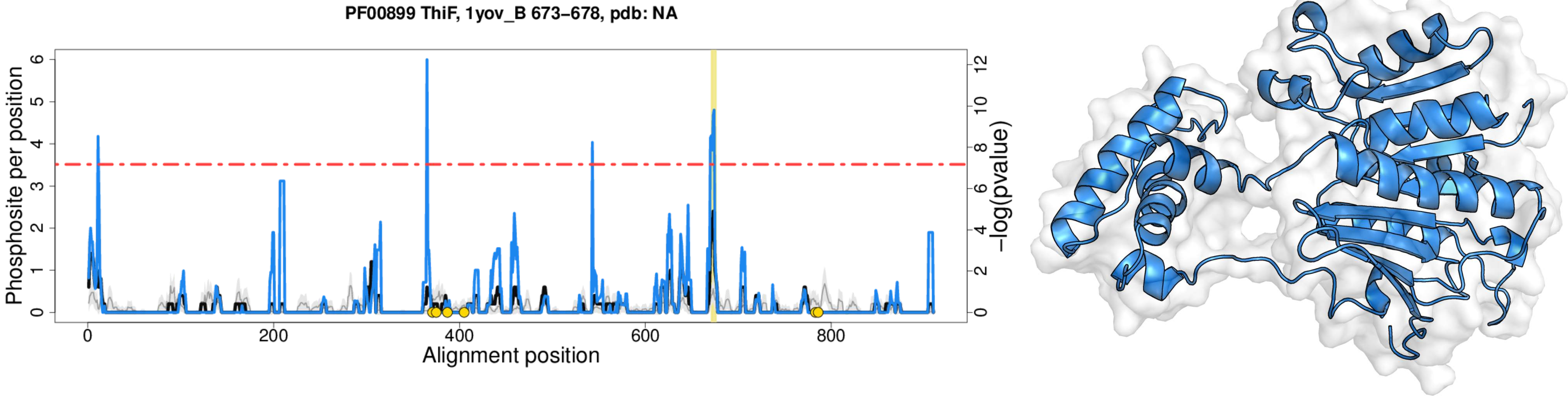

PF00899 ThiF, 1yov_B 673–678, pdb: NA
Phosphosite per position
0
1
2
3
4
5
6
Alignment position
0
200
400
600
800
−log(pvalue)
0
2
4
6
8
10
12

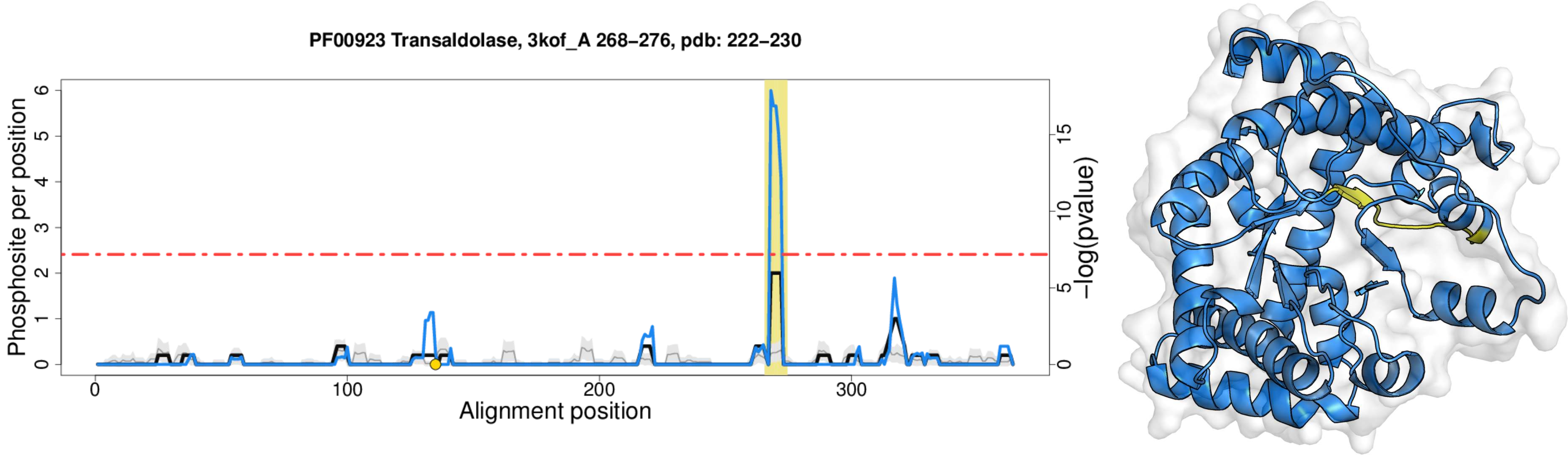
PF00923 Transaldolase, 3kof_A 268–276, pdb: 222–230
Phosphosite per position
Alignment position
−log(pvalue)
0
1
2
3
4
5
6
0
100
200
300
0
5
10
15

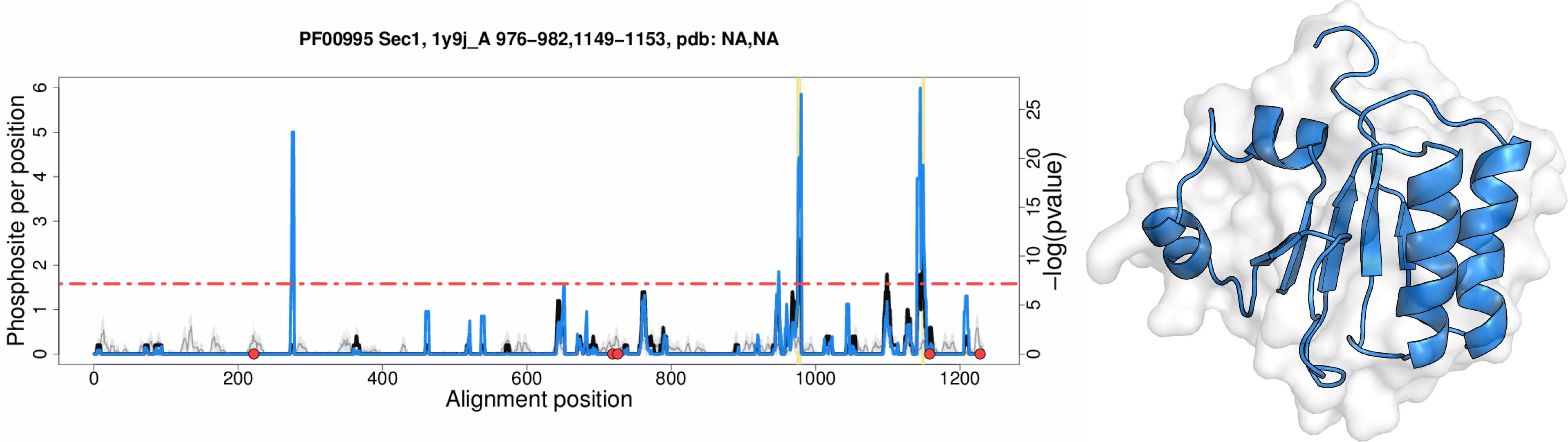
PF00995 Sec1, 1y9j_A 976-982,1149-1153, pdb: NA,NA
Phosphosite per position
-log(pvalue)
Alignment position
0
1
2
3
4
5
6
0
5
10
15
20
25
0
200
400
600
800
1000
1200

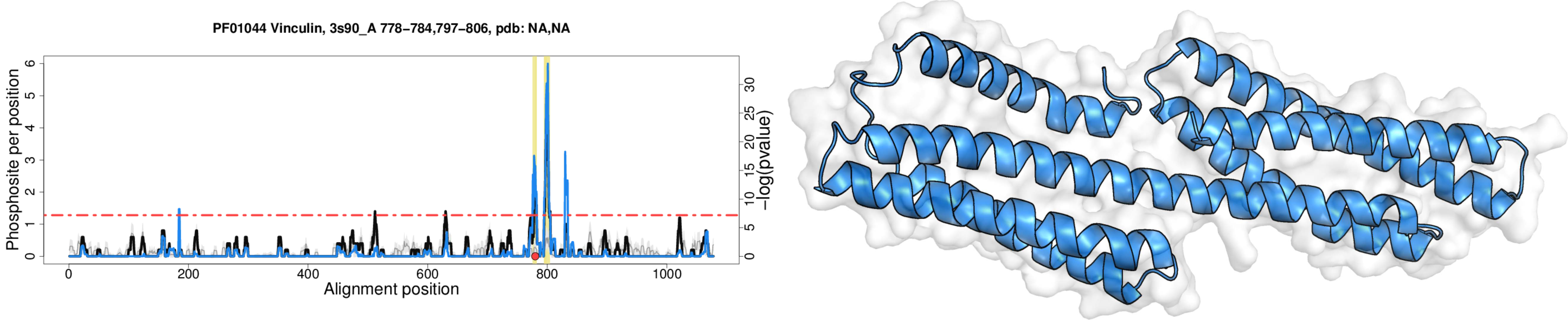
PF01044 Vinculin, 3s90_A 778−784,797−806, pdb: NA,NA
Phosphosite per position
−log(pvalue)
Alignment position
0
1
2
3
4
5
6
0
5
10
15
20
25
30
0
200
400
600
800
1000

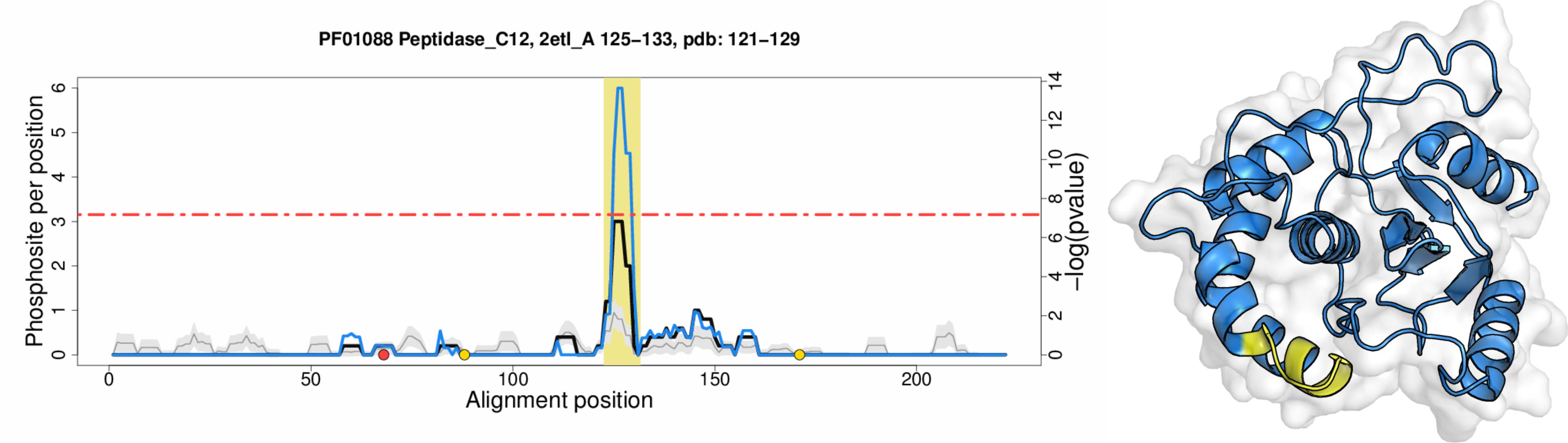
PF01088 Peptidase_C12, 2etl_A 125–133, pdb: 121–129
Phosphosite per position
Alignment position
−log(pvalue)
0
1
2
3
4
5
6
0
50
100
150
200
0
2
4
6
8
10
12
14

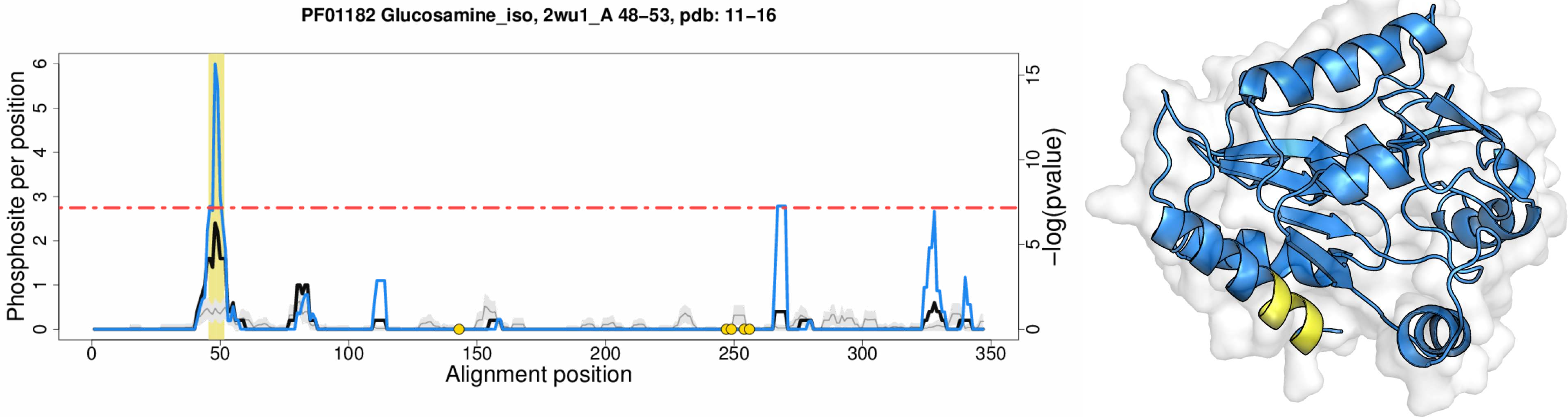
PF01182 Glucosamine_iso, 2wu1_A 48–53, pdb: 11–16
Phosphosite per position
0
1
2
3
4
5
6
Alignment position
0
50
100
150
200
250
300
350
−log(pvalue)
0
5
10
15

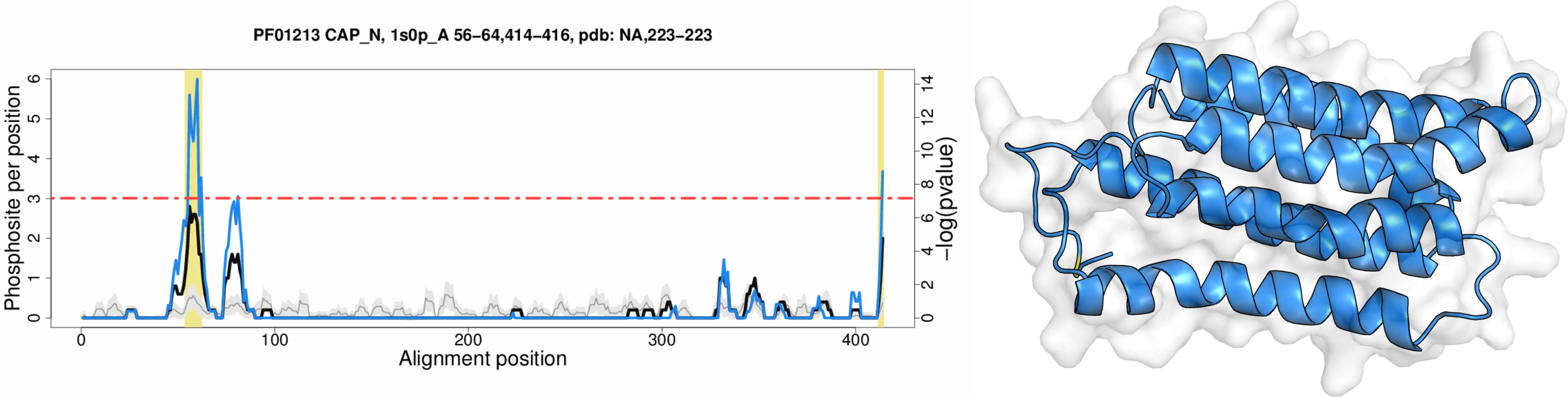
PF01213 CAP_N, 1s0p_A 56–64,414–416, pdb: NA,223–223
Phosphosite per position
Alignment position
−log(pvalue)
0
1
2
3
4
5
6
0
100
200
300
400
0
2
4
6
8
10
12
14

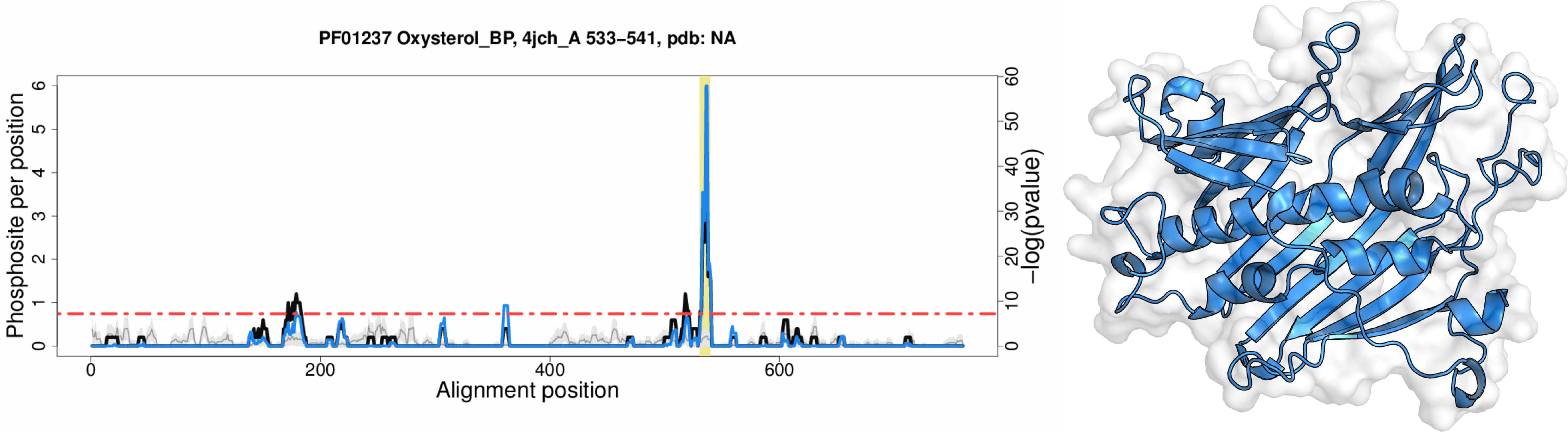
PF01237 Oxysterol_BP, 4jch_A 533−541, pdb: NA
Phosphosite per position
0
1
2
3
4
5
6
−log(pvalue)
0
10
20
30
40
50
60
0
200
400
600
Alignment position

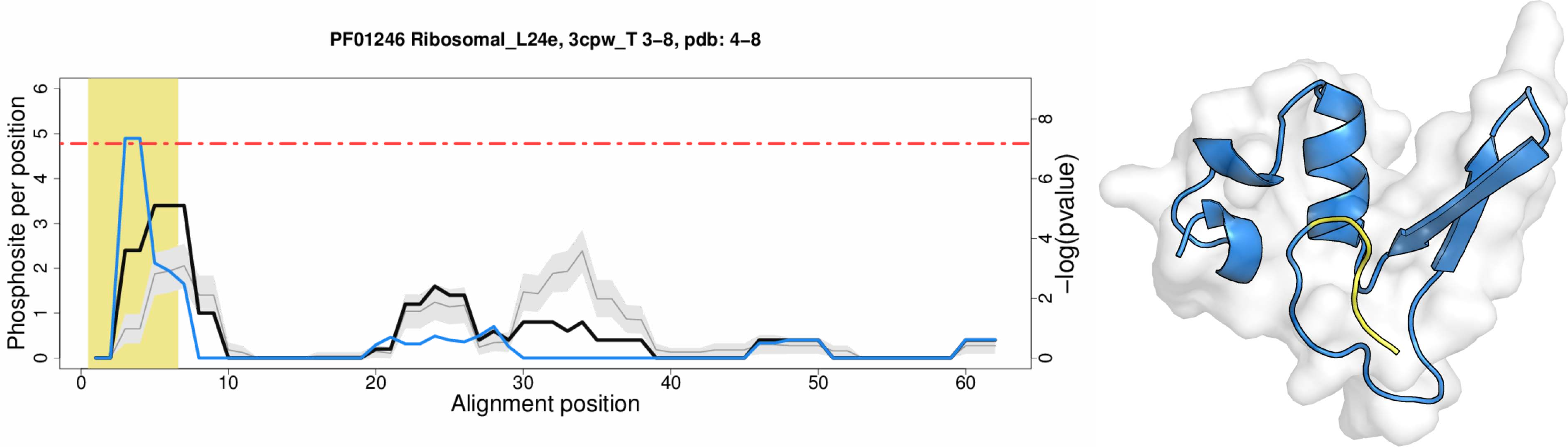
PF01246 Ribosomal_L24e, 3cpw_T 3–8, pdb: 4–8
Phosphosite per position
0
1
2
3
4
5
6
−log(pvalue)
0
2
4
6
8
Alignment position
0
10
20
30
40
50
60

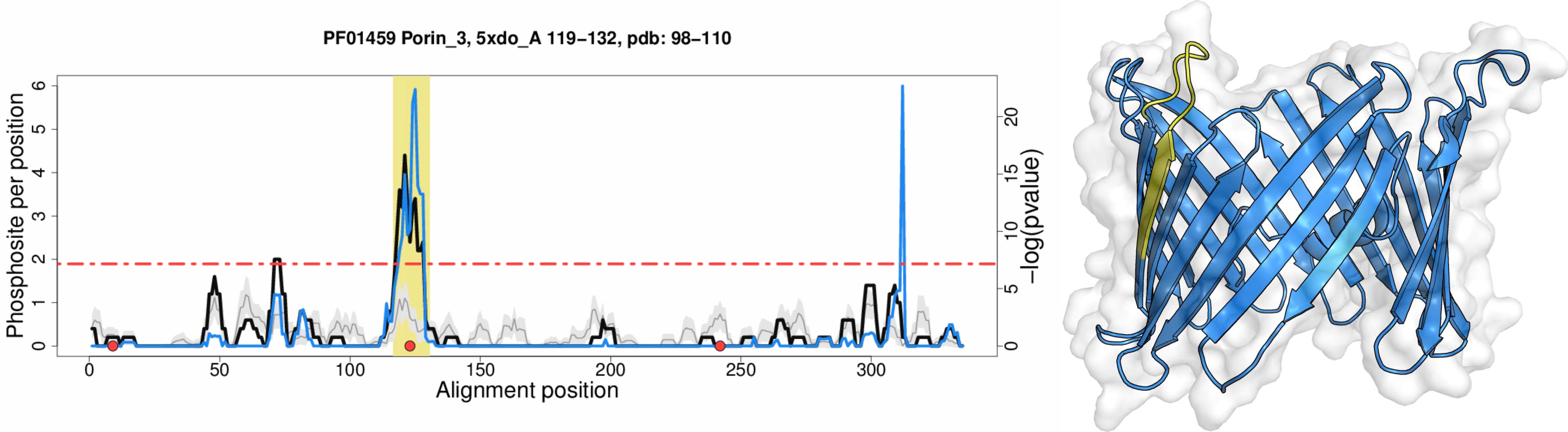
PF01459 Porin_3, 5xdo_A 119−132, pdb: 98−110
Phosphosite per position
−log(pvalue)
Alignment position
0
1
2
3
4
5
6
0
5
10
15
20
0
50
100
150
200
250
300

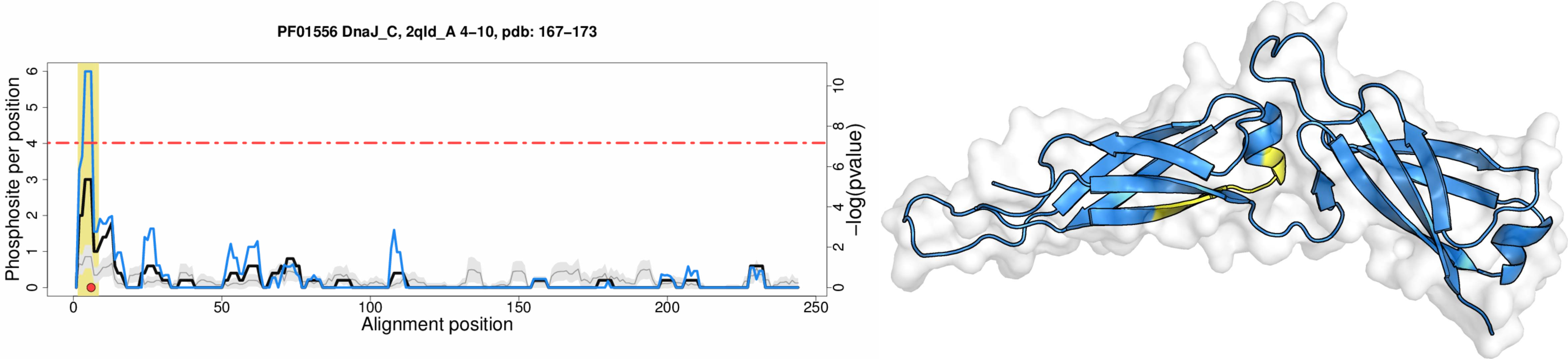

PF01556 DnaJ_C, 2qld_A 4–10, pdb: 167–173
Phosphosite per position
−log(pvalue)
Alignment position
0
1
2
3
4
5
6
0
2
4
6
8
10
0
50
100
150
200
250

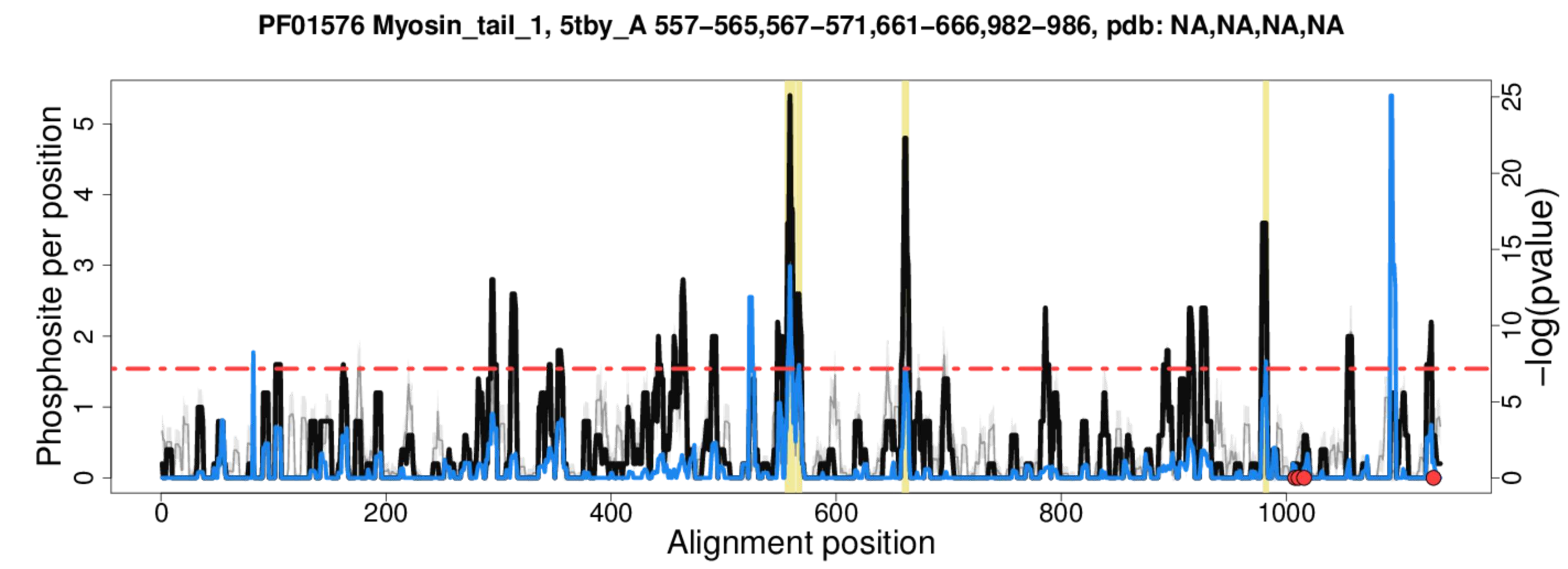
PF01576 Myosin_tail_1, 5tby_A 557−565,567−571,661−666,982−986, pdb: NA,NA,NA,NA
Phosphosite per position
−log(pvalue)
Alignment position
0
200
400
600
800
1000
1
2
3
4
5
5
10
15
20
25

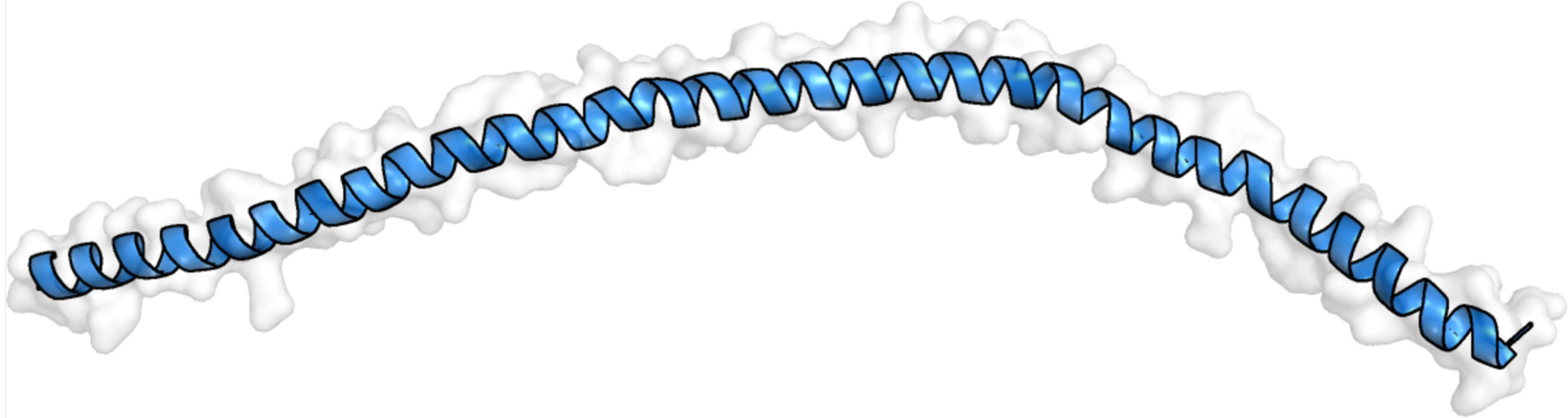

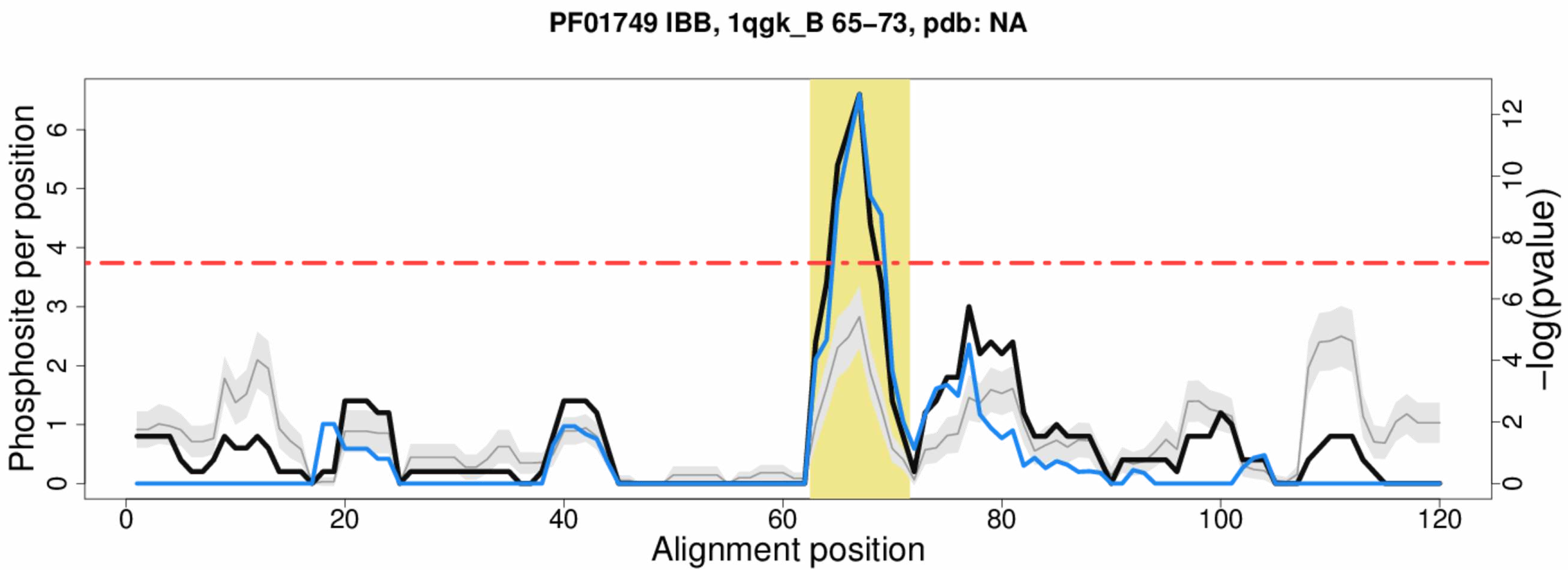
PF01749 IBB, 1qgk_B 65–73, pdb: NA
Phosphosite per position
0
1
2
3
4
5
6
–log(pvalue)
0
2
4
6
8
10
12
0
20
40
60
80
100
120
Alignment position

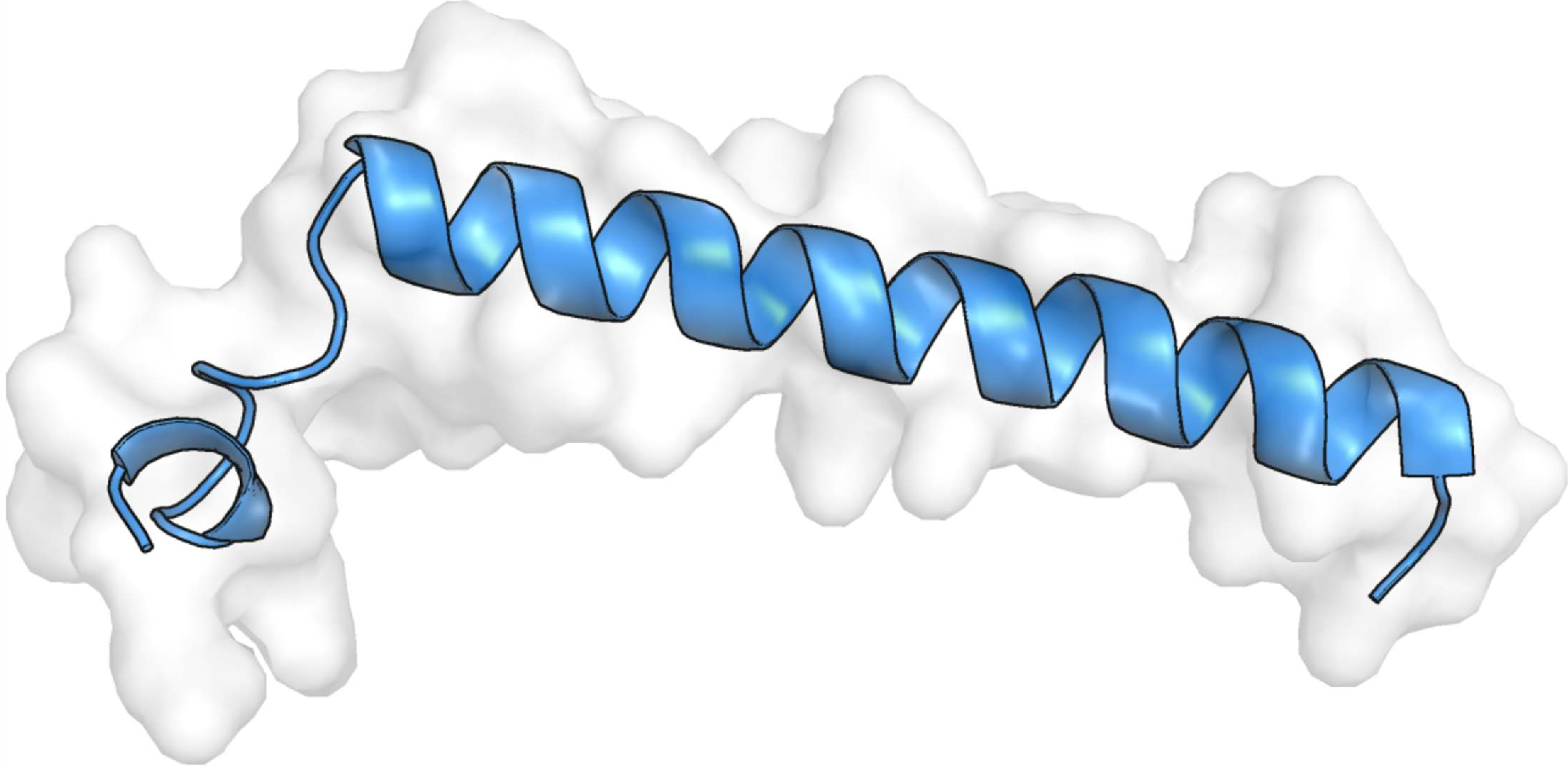

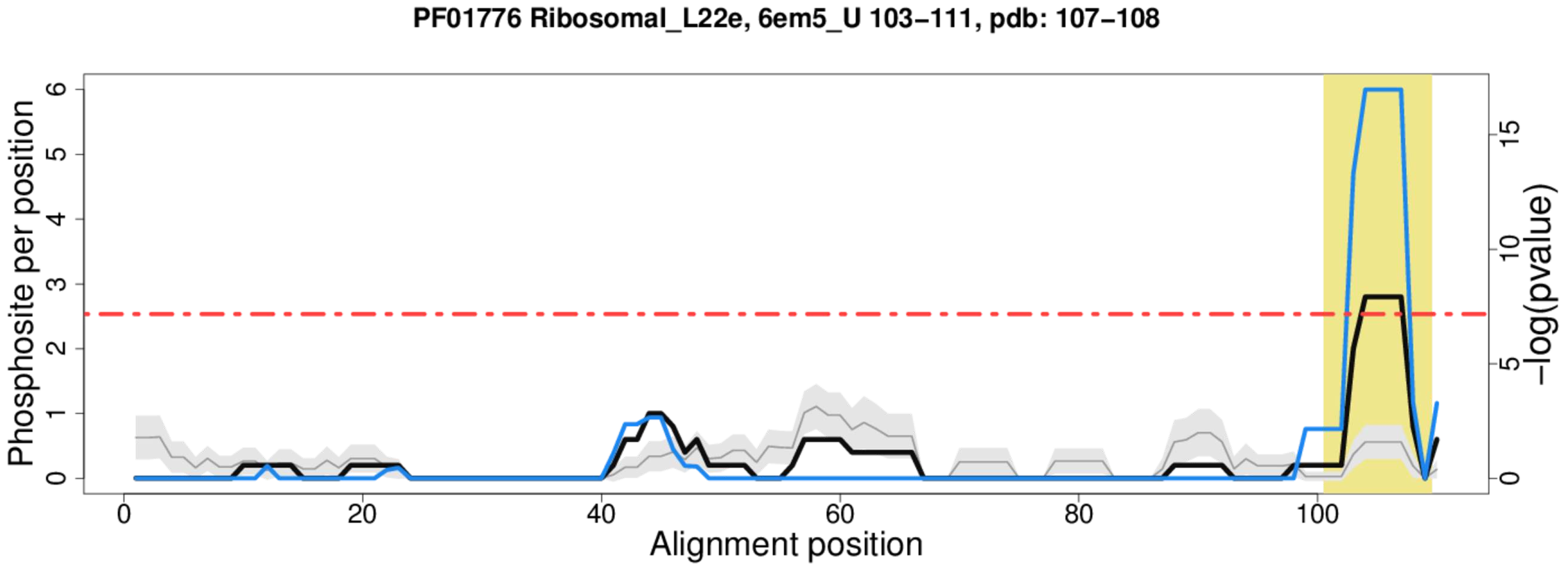

PF01776 Ribosomal_L22e, 6em5_U 103−111, pdb: 107−108
Phosphosite per position
0
1
2
3
4
5
6
0
20
40
60
80
100
Alignment position
−log(pvalue)
0
5
10
15

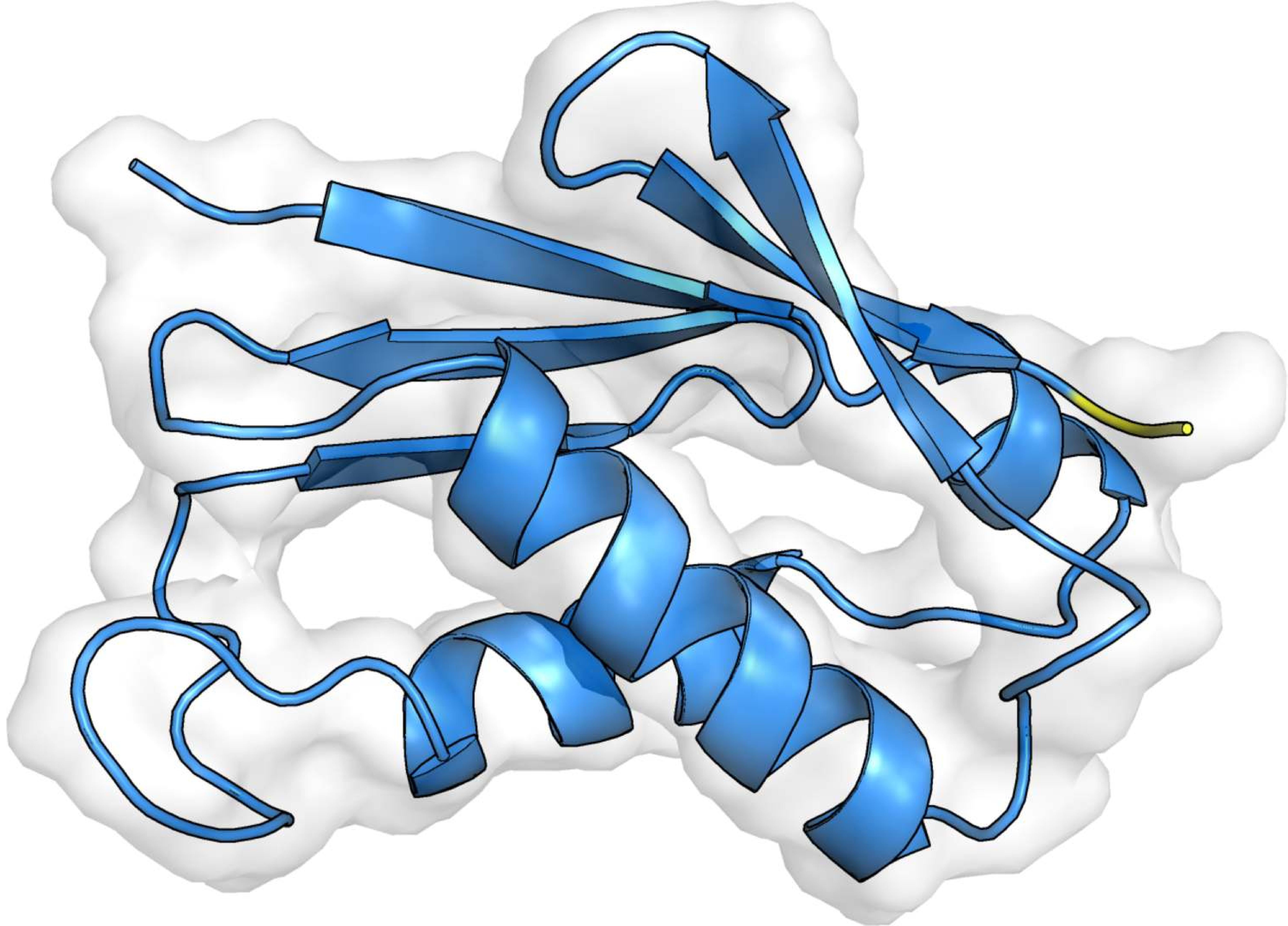

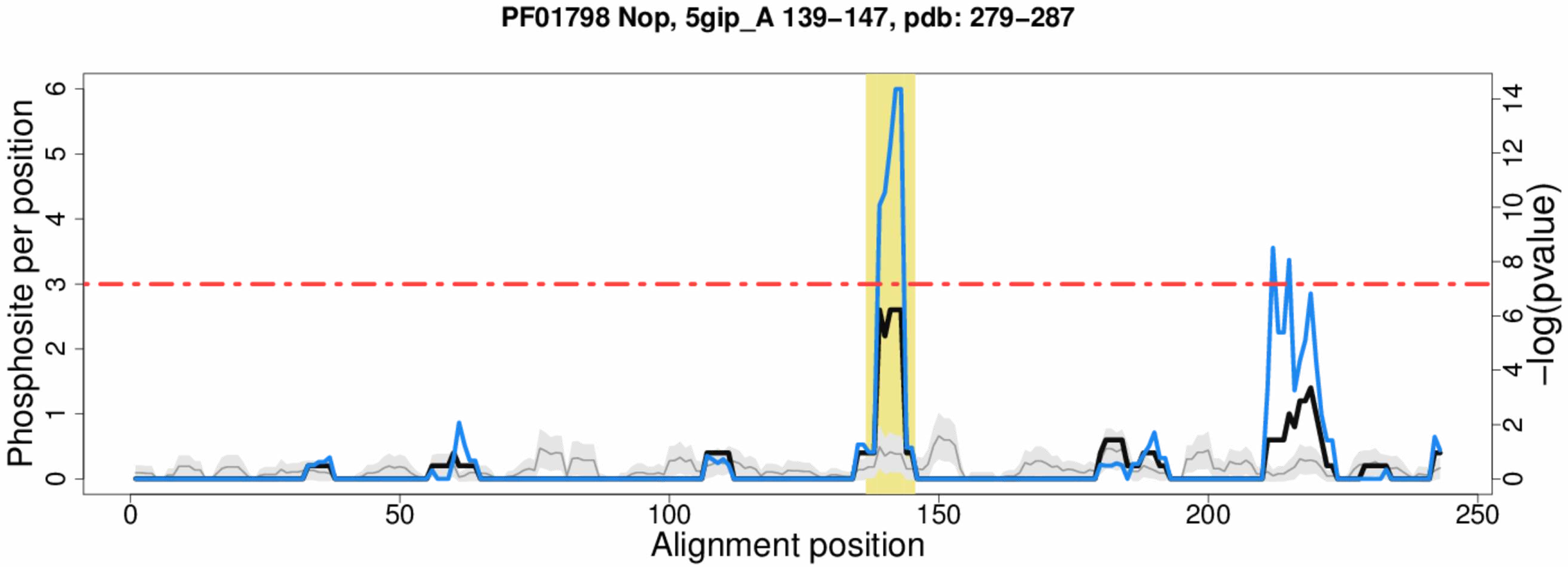

PF01798 Nop, 5gip_A 139−147, pdb: 279−287
Phosphosite per position
Alignment position
−log(pvalue)
0
1
2
3
4
5
6
0
50
100
150
200
250
0
2
4
6
8
10
12
14

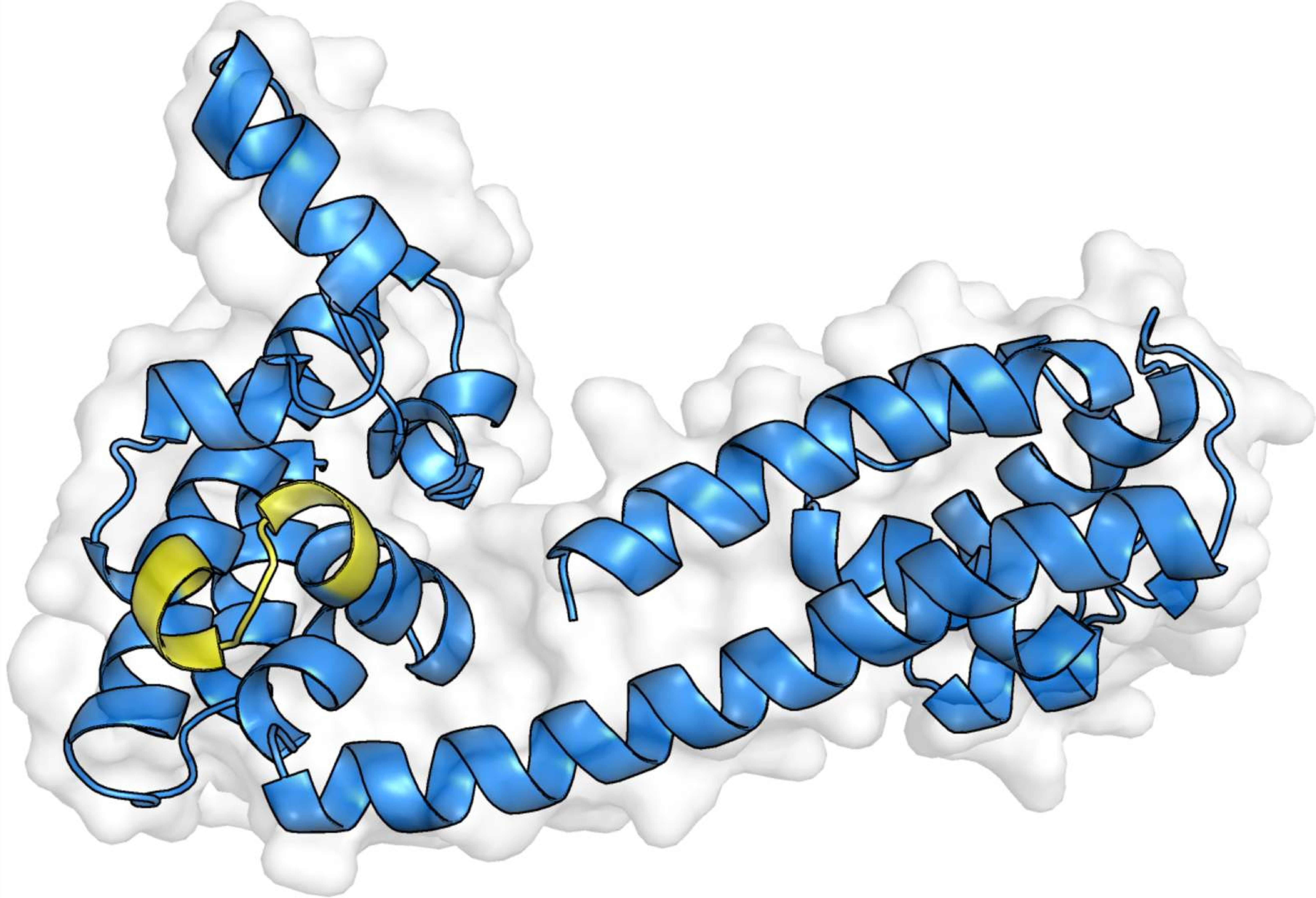

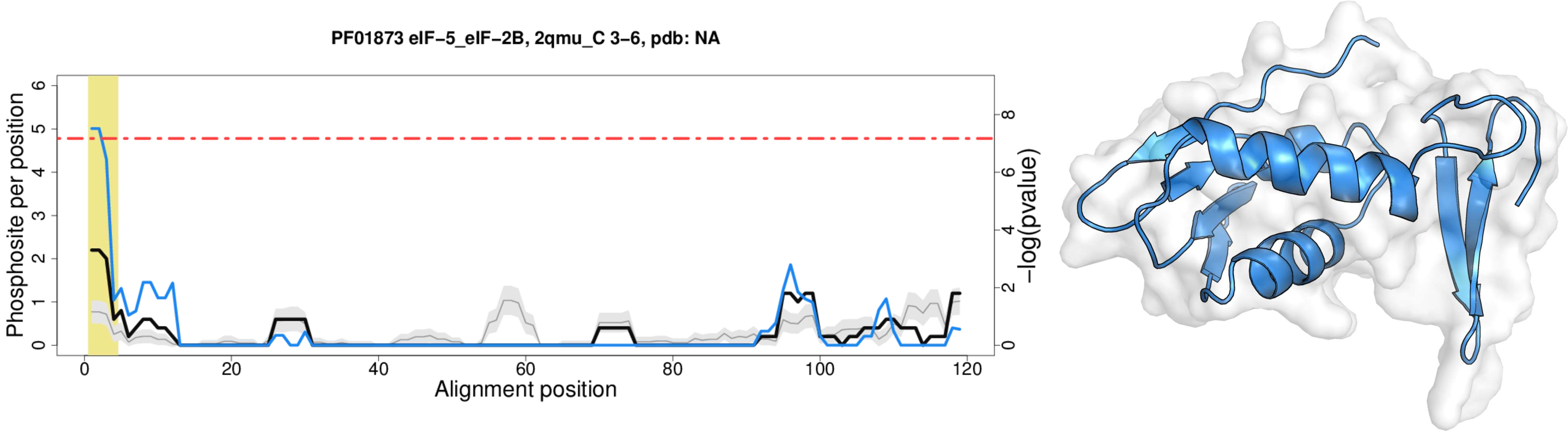

PF01873 eIF−5_eIF−2B, 2qmu_C 3−6, pdb: NA
Phosphosite per position
0
1
2
3
4
5
6
−log(pvalue)
0
2
4
6
8
Alignment position
0
20
40
60
80
100
120

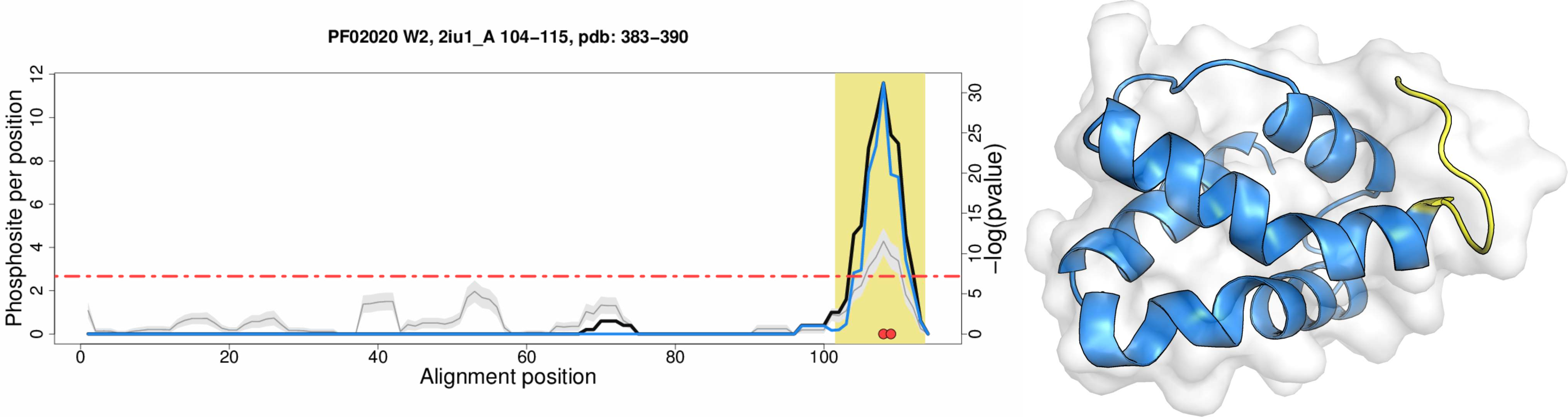
PF02020 W2, 2iu1_A 104–115, pdb: 383–390
Phosphosite per position
0
2
4
6
8
10
12
Alignment position
0
20
40
60
80
100
−log(pvalue)
0
5
10
15
20
25
30

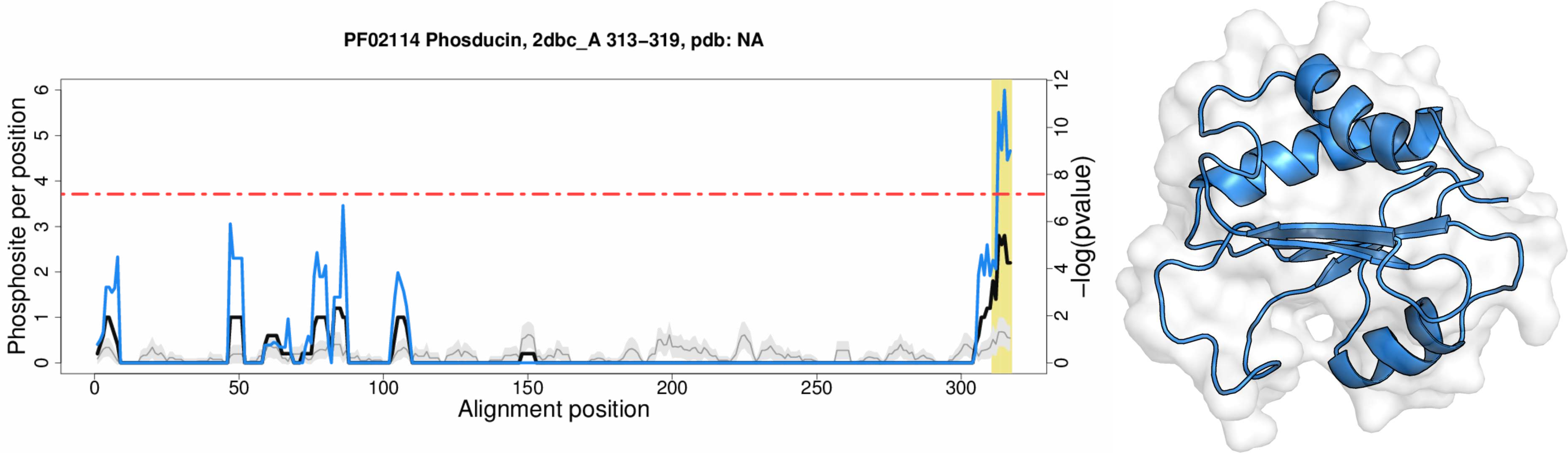
PF02114 Phosducin, 2dbc_A 313–319, pdb: NA
Phosphosite per position
0
1
2
3
4
5
6
0
50
100
150
200
250
300
Alignment position
−log(pvalue)
0
2
4
6
8
10
12

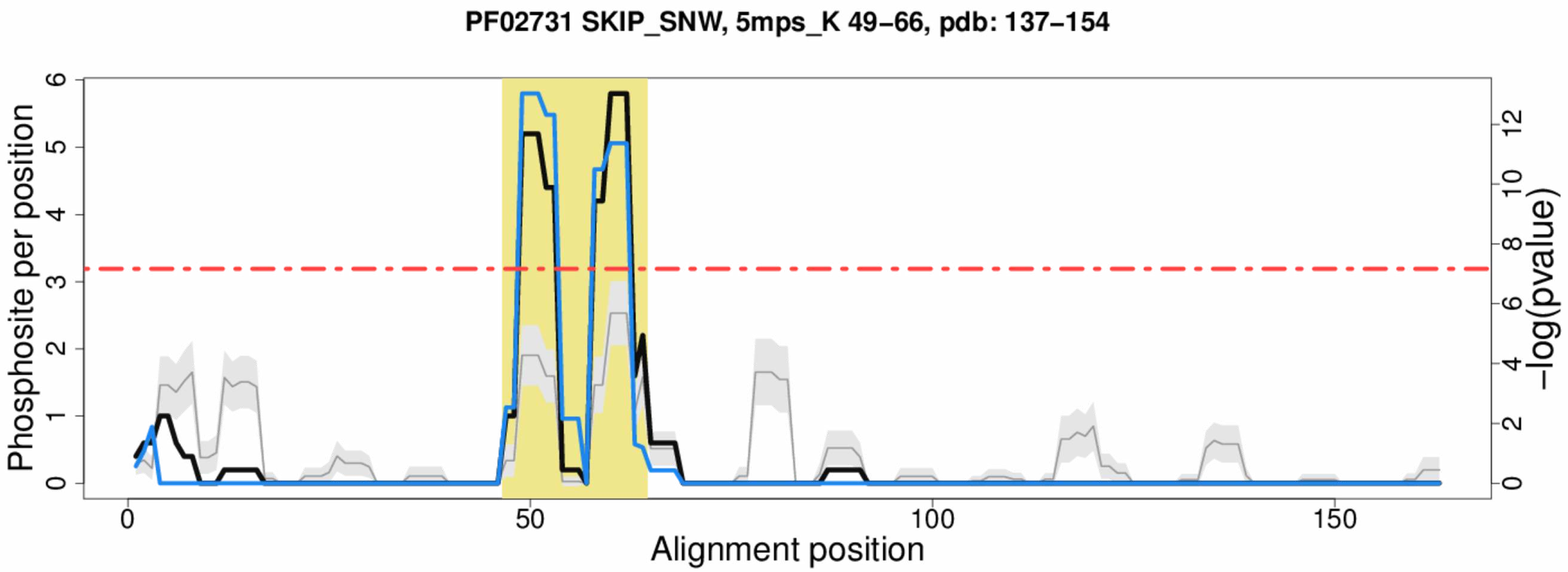

PF02731 SKIP_SNW, 5mps_K 49−66, pdb: 137−154
Phosphosite per position
−log(pvalue)
Alignment position
0
50
100
150

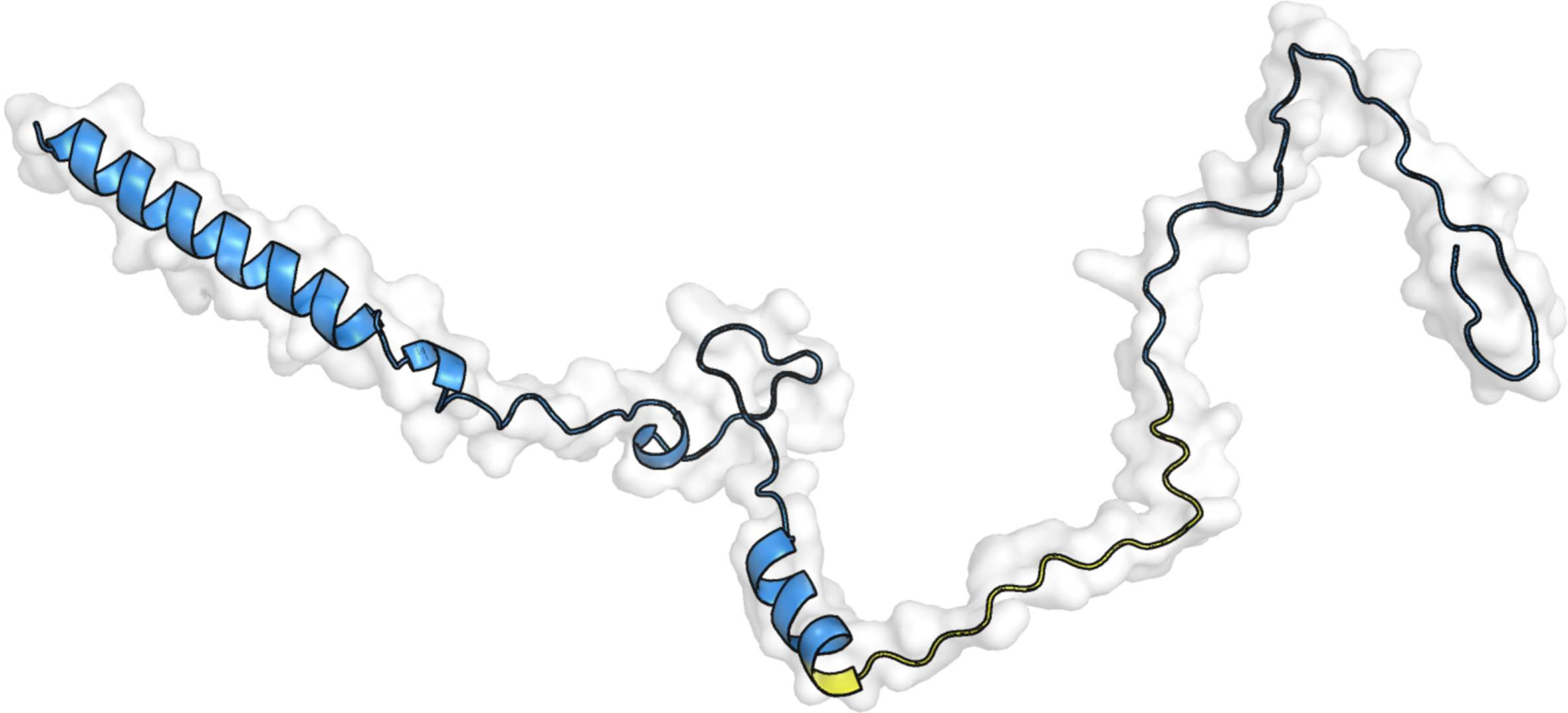

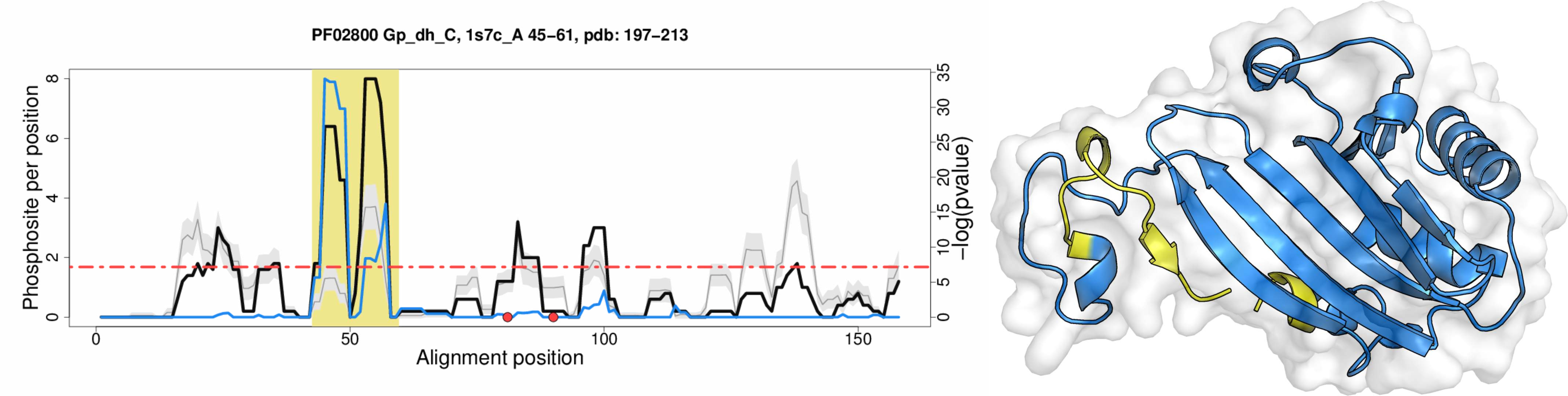

PF02800 Gp_dh_C, 1s7c_A 45−61, pdb: 197−213
Phosphosite per position
−log(pvalue)
Alignment position
0
2
4
6
8
0
50
100
150
0
5
10
15
20
25
30
35

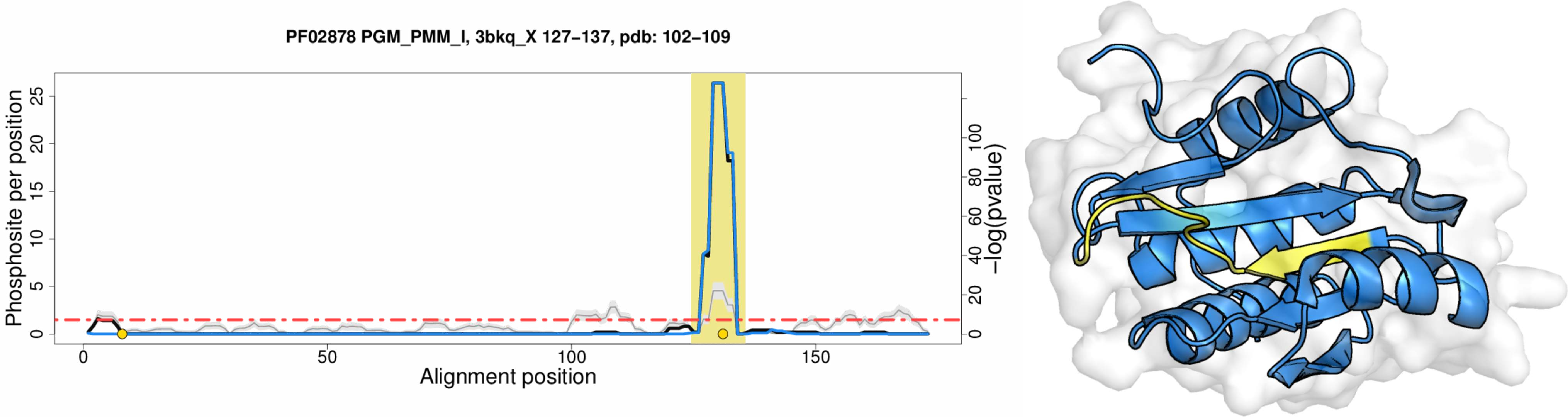
PF02878 PGM_PMM_I, 3bkq_X 127–137, pdb: 102–109
Phosphosite per position
0
5
10
15
20
25
Alignment position
0
50
100
150
−log(pvalue)
0
20
40
60
80
100

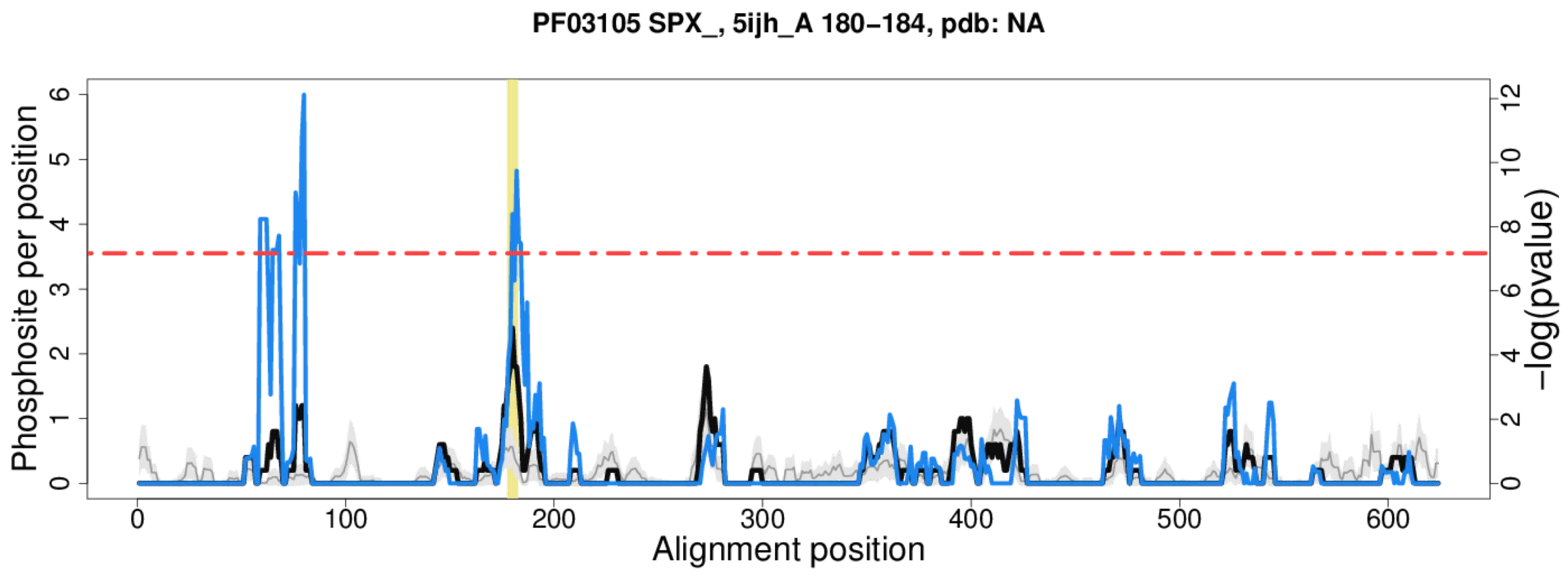
PF03105 SPX_, 5ijh_A 180−184, pdb: NA
Phosphosite per position
−log(pvalue)
Alignment position
0
1
2
3
4
5
6
0
2
4
6
8
10
12
0
100
200
300
400
500
600

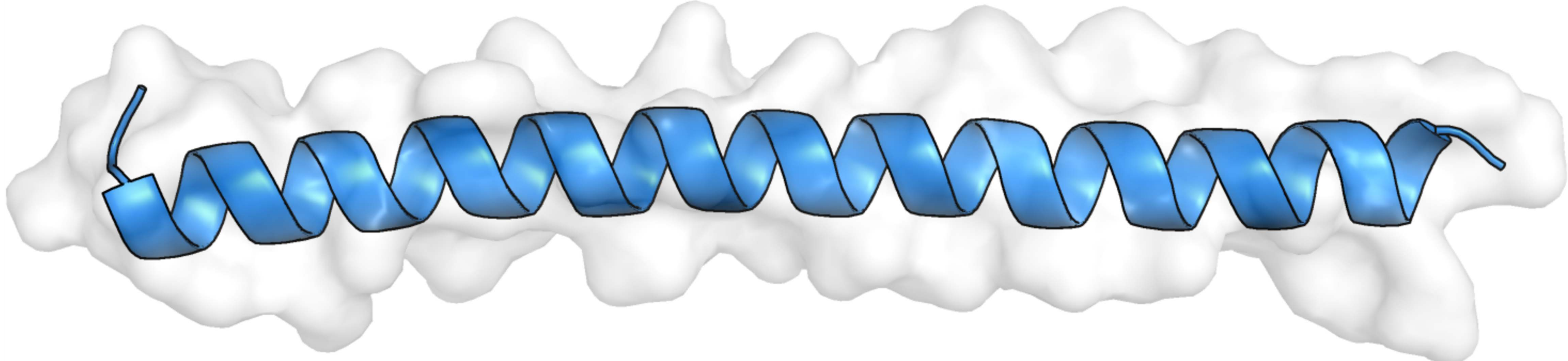

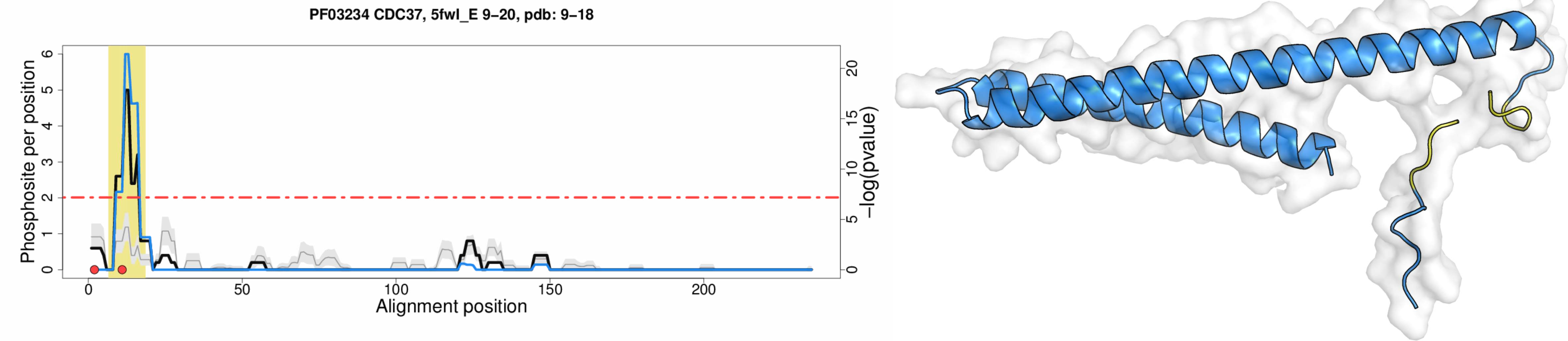

PF03234 CDC37, 5fwl_E 9–20, pdb: 9–18
Phosphosite per position
0
1
2
3
4
5
6
−log(pvalue)
0
5
10
15
20
Alignment position
0
50
100
150
200

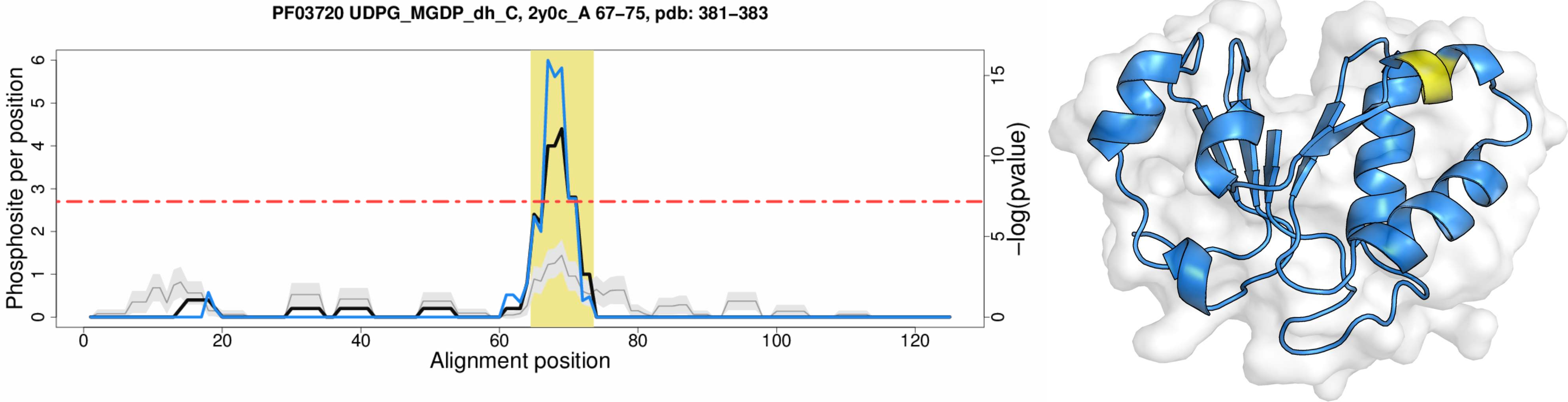

PF03720 UDPG_MGDP_dh_C, 2y0c_A 67–75, pdb: 381–383
Phosphosite per position
0
1
2
3
4
5
6
Alignment position
0
20
40
60
80
100
120
−log(pvalue)
0
5
10
15

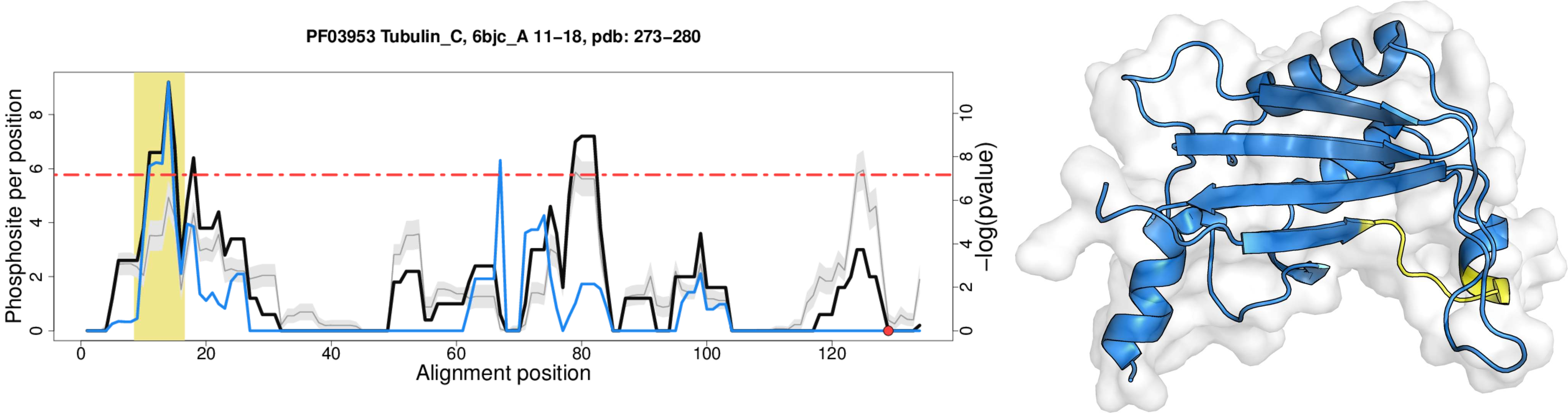
PF03953 Tubulin_C, 6bjc_A 11−18, pdb: 273−280
Phosphosite per position
Alignment position
−log(pvalue)
0
2
4
6
8
0
20
40
60
80
100
120
0
2
4
6
8
10

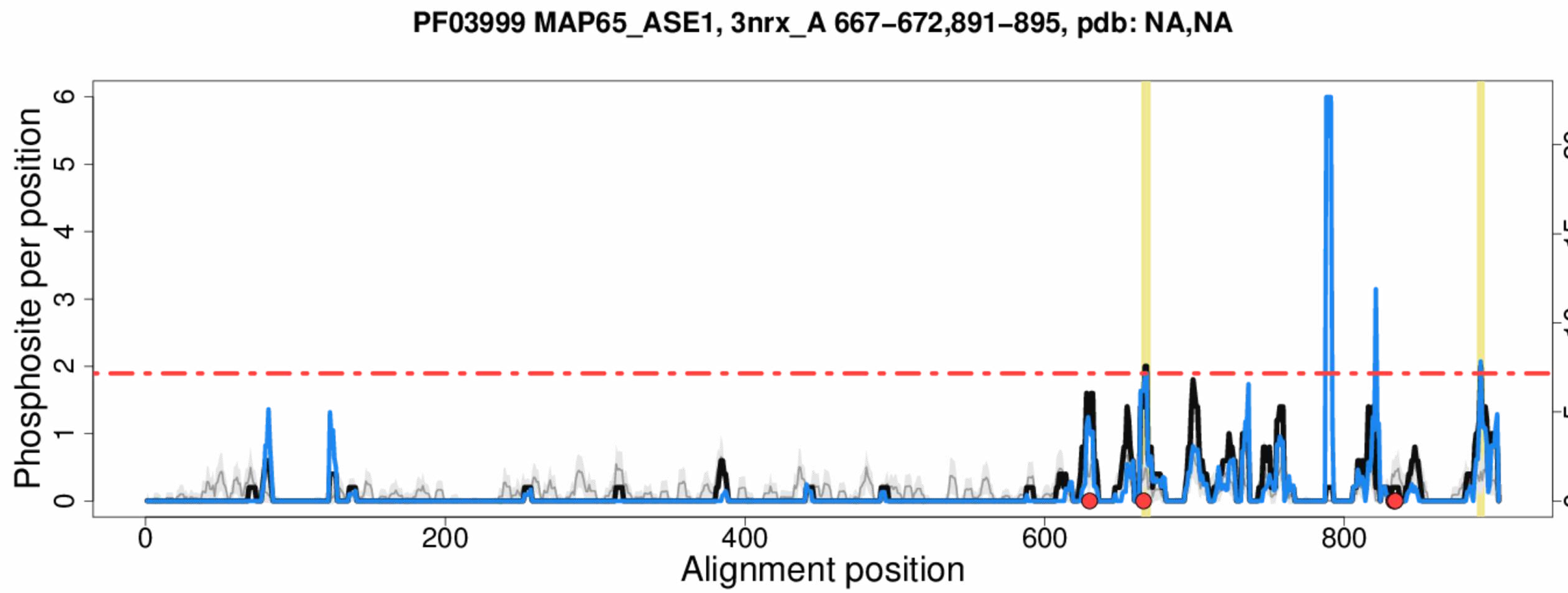

PF03999 MAP65_ASE1, 3nrx_A 667−672,891−895, pdb: NA,NA
Phosphosite per position
−log(pvalue)
Alignment position
0
200
400
600
800
1
2
3
4
5
6
5
10
15
20

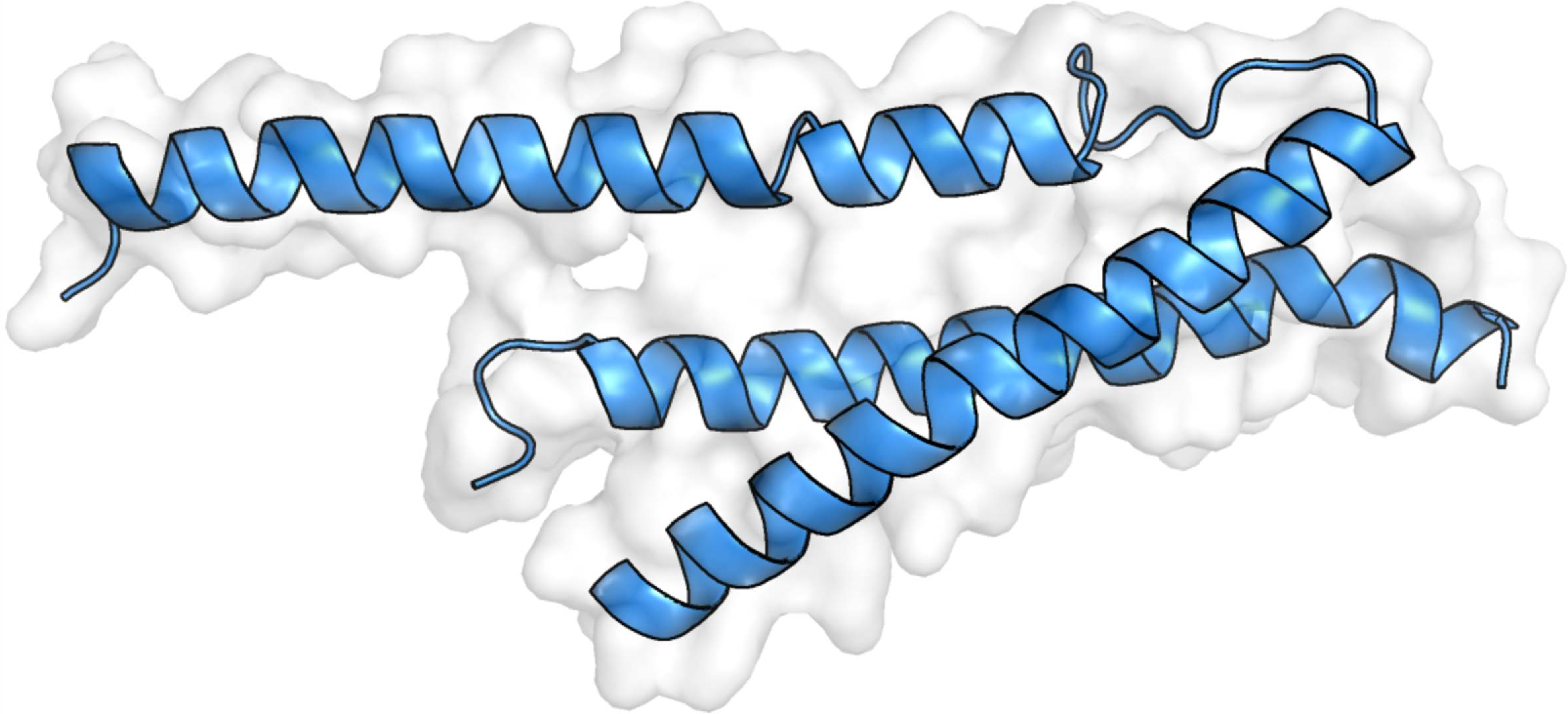

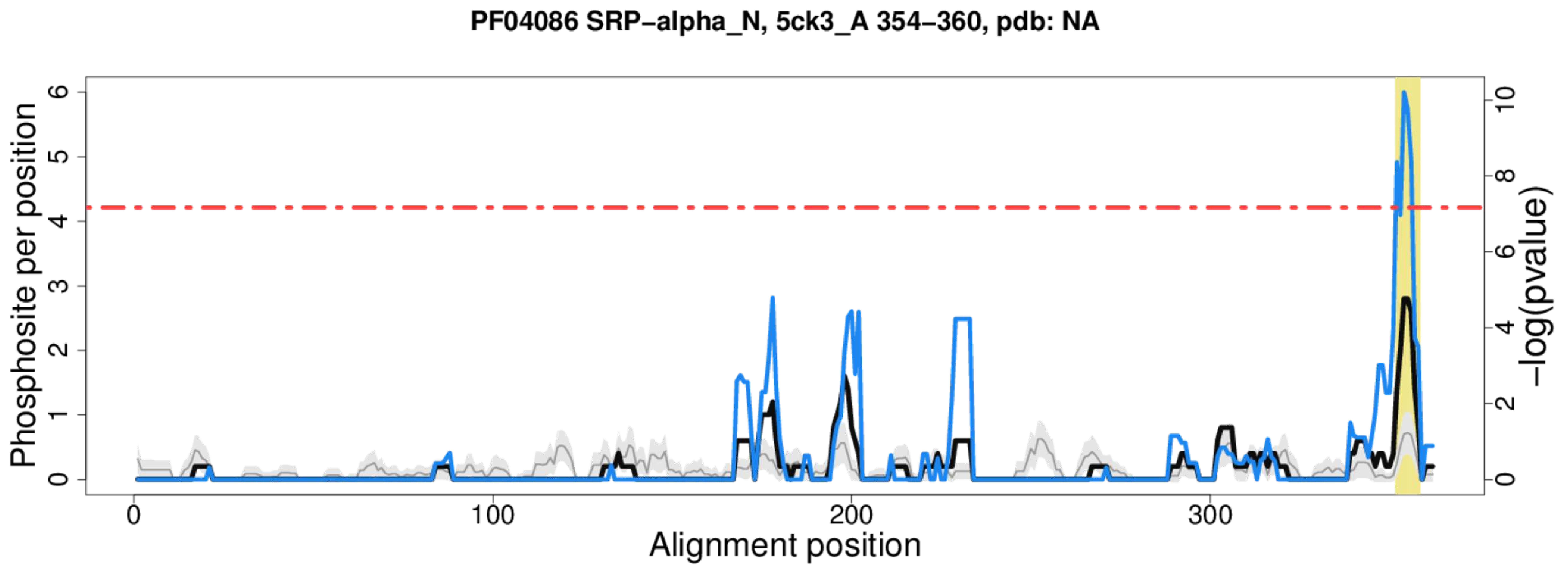
PF04086 SRP−alpha_N, 5ck3_A 354−360, pdb: NA
Phosphosite per position
0
1
2
3
4
5
6
−log(pvalue)
0
2
4
6
8
10
0
100
200
300
Alignment position

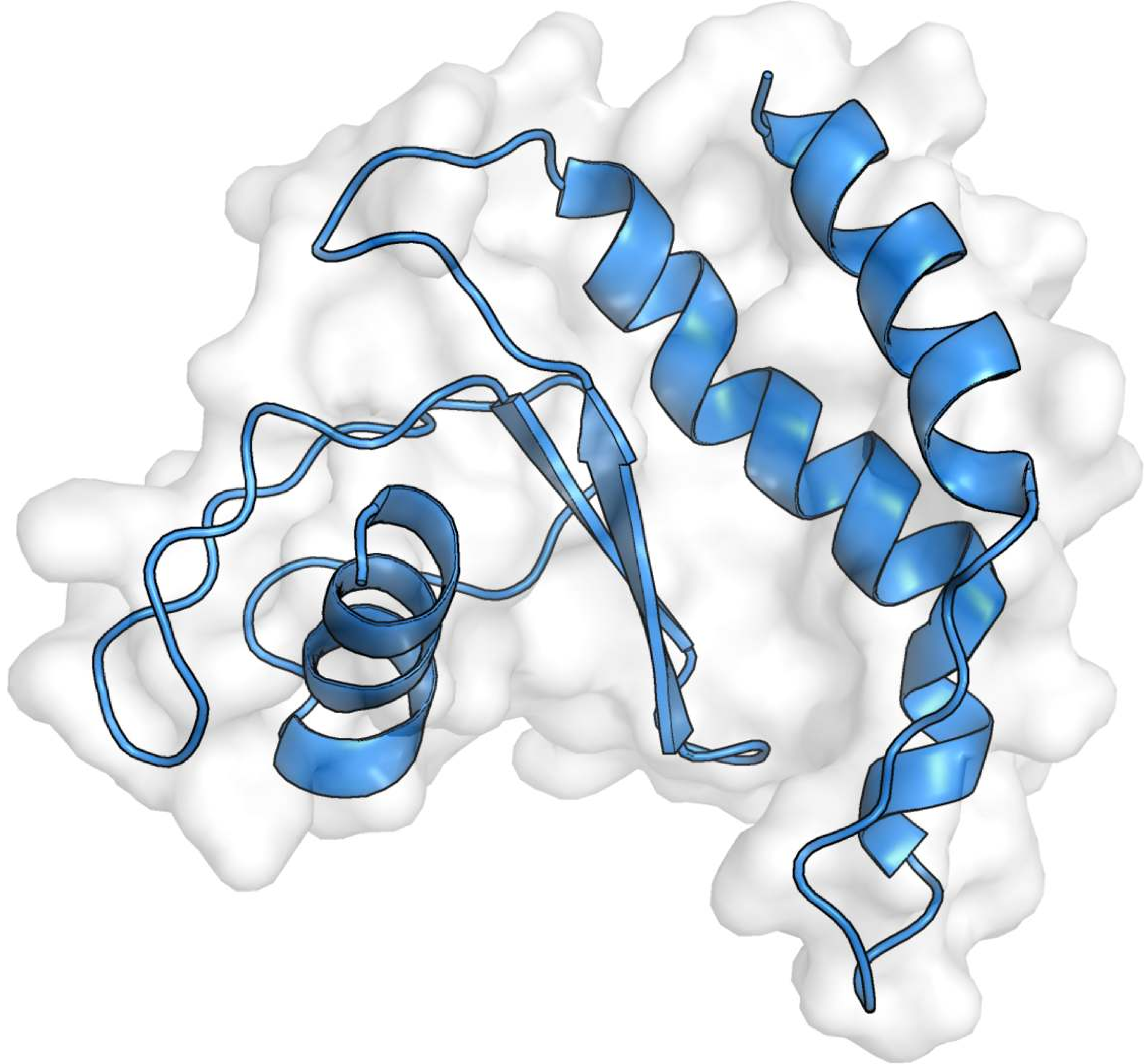

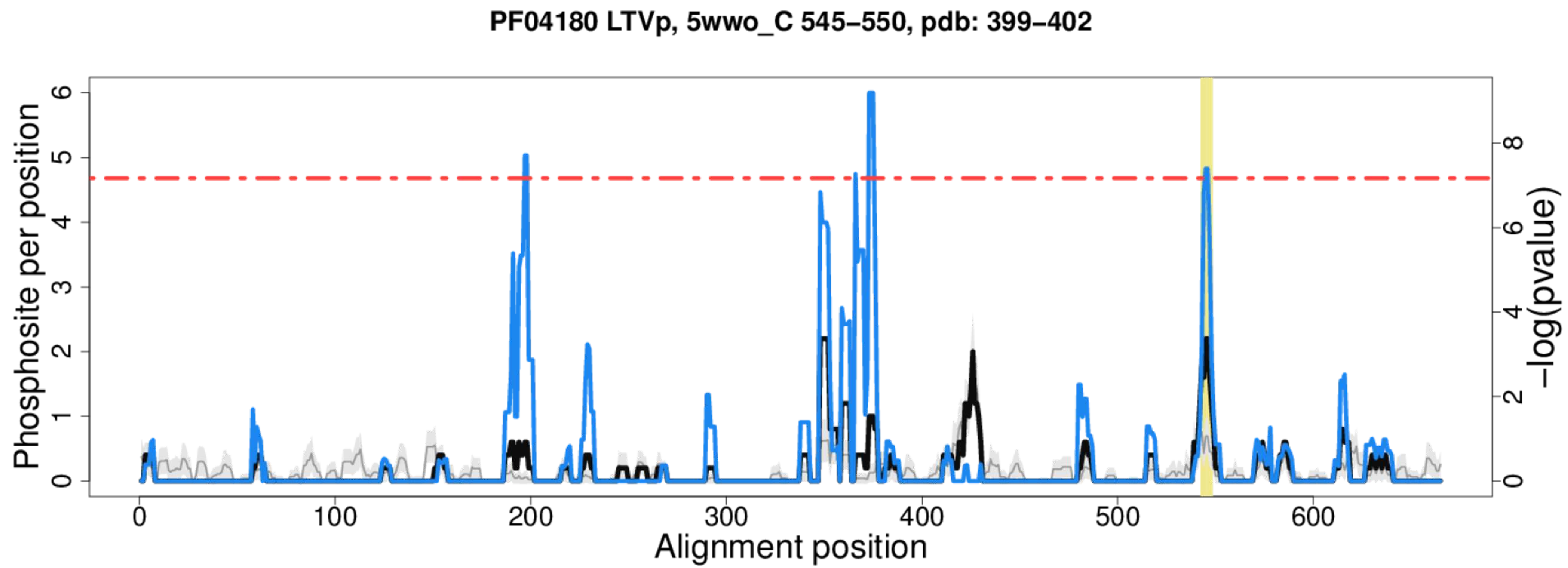
PF04180 LTVp, 5wwo_C 545–550, pdb: 399–402
Phosphosite per position
–log(pvalue)
Alignment position
0
1
2
3
4
5
6
0
2
4
6
8
0
100
200
300
400
500
600
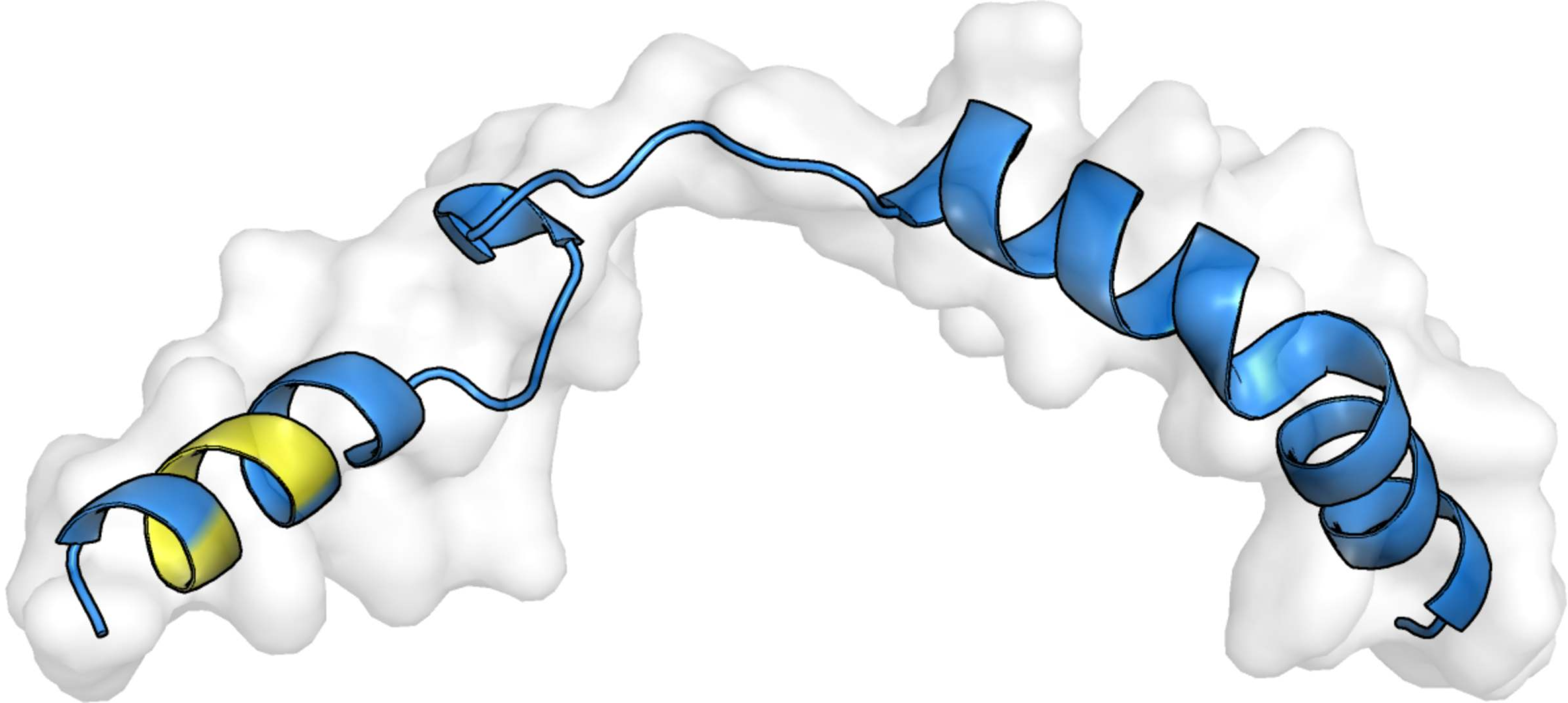

PF04410 Gar1, 2ey4_C 142–150, pdb: NA

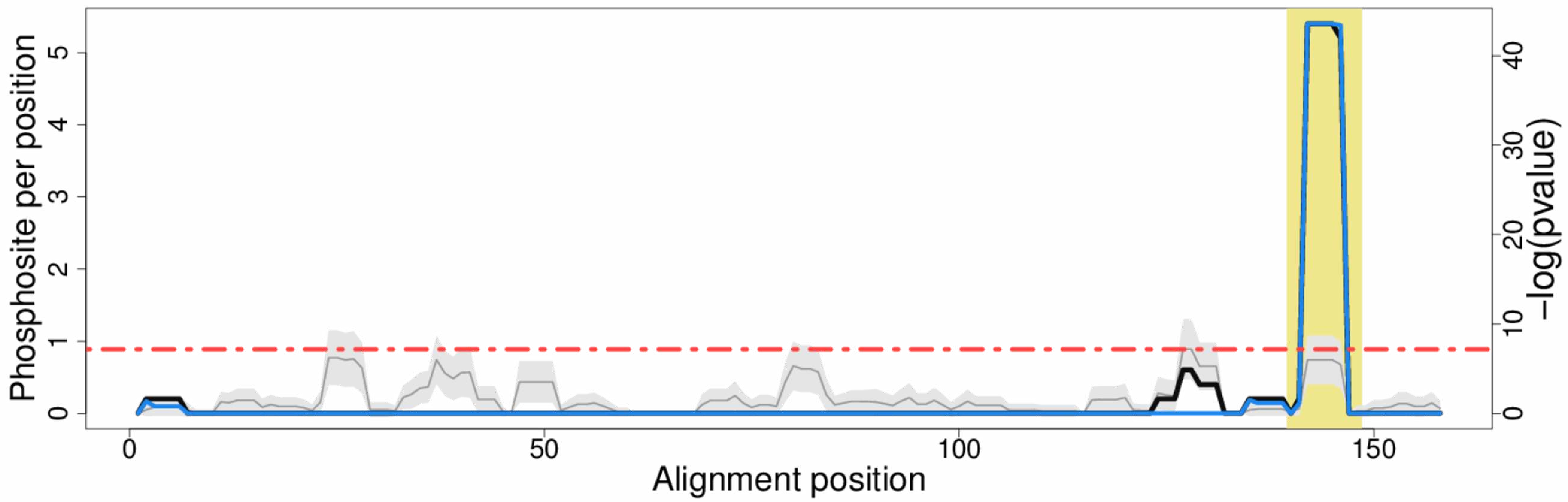

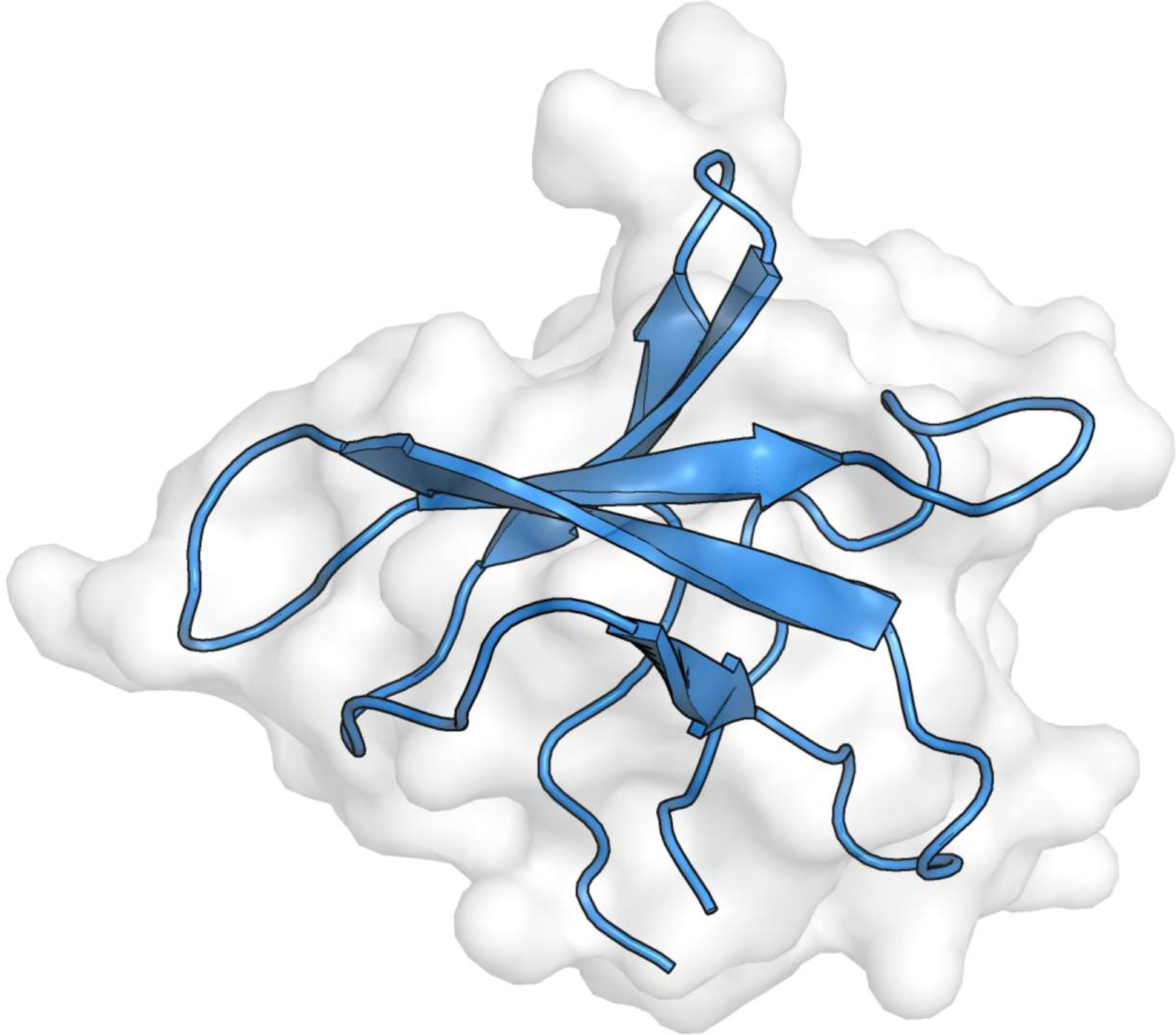

PF04427 Brix, 6c0f_I 297–306,309–317, pdb: NA,NA

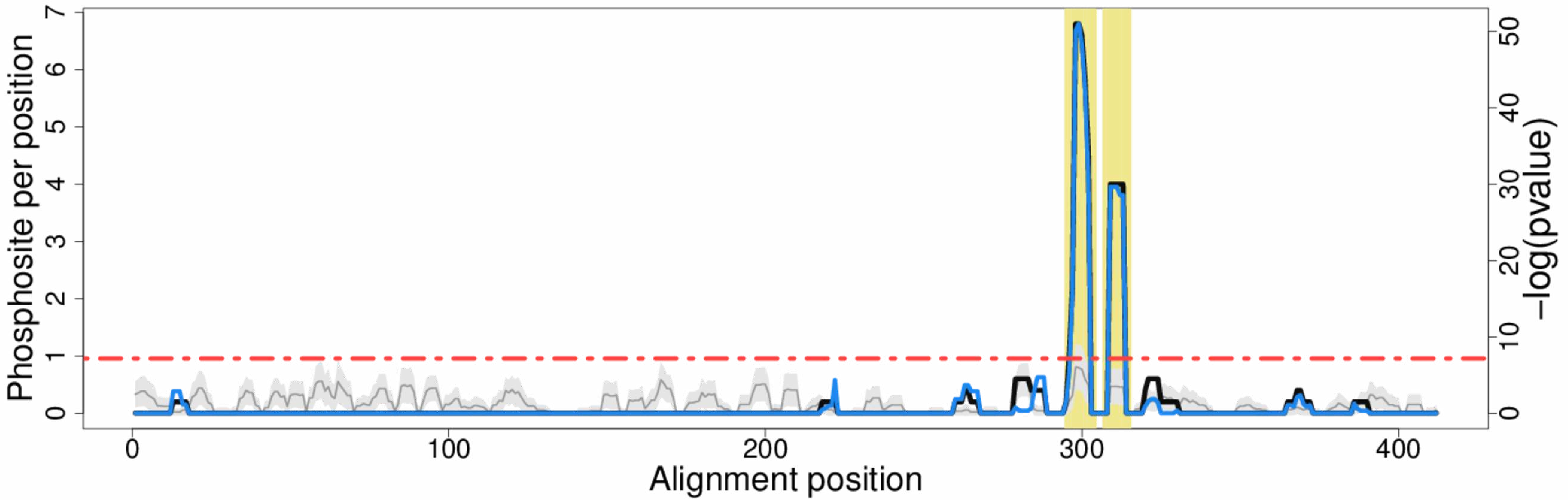

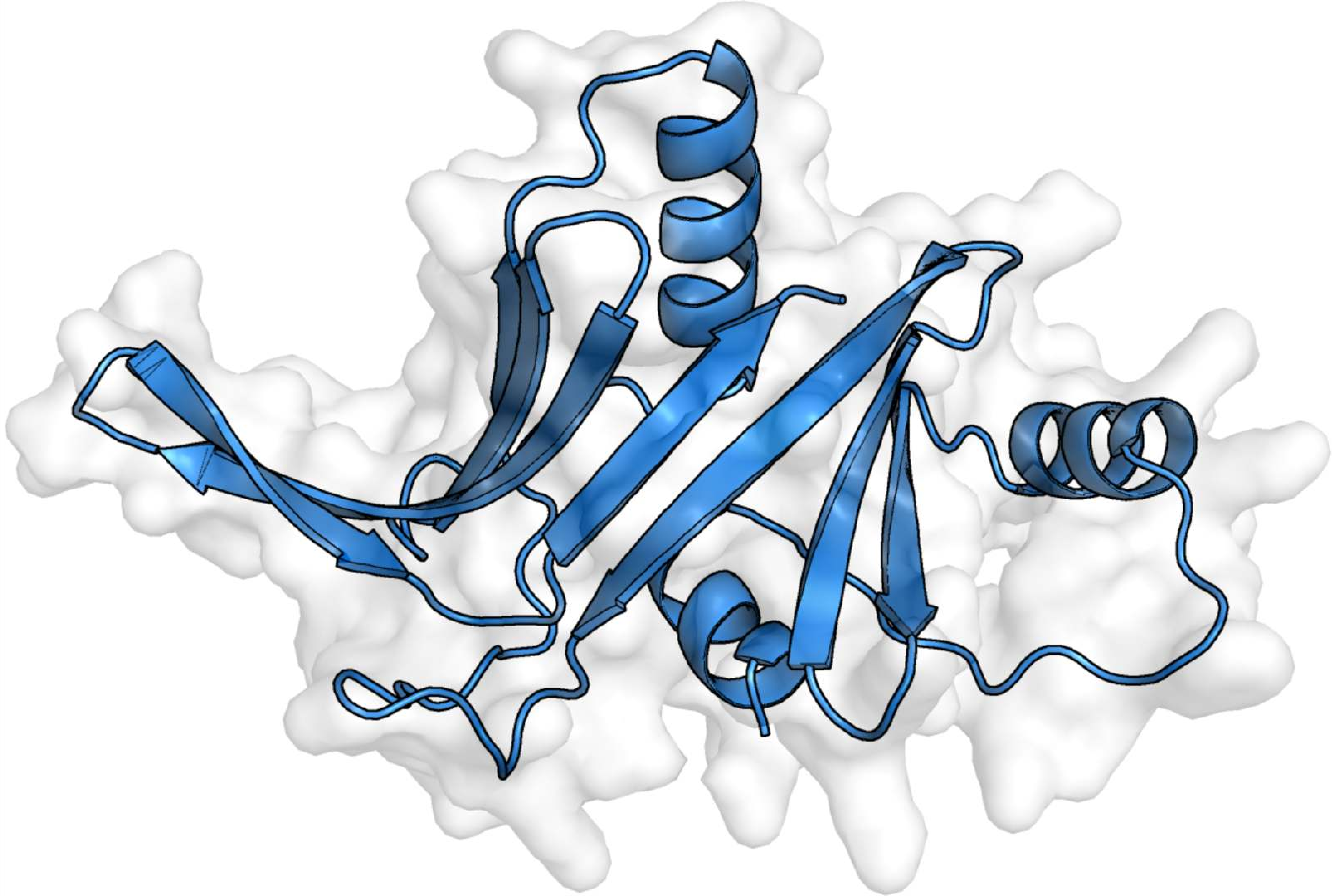

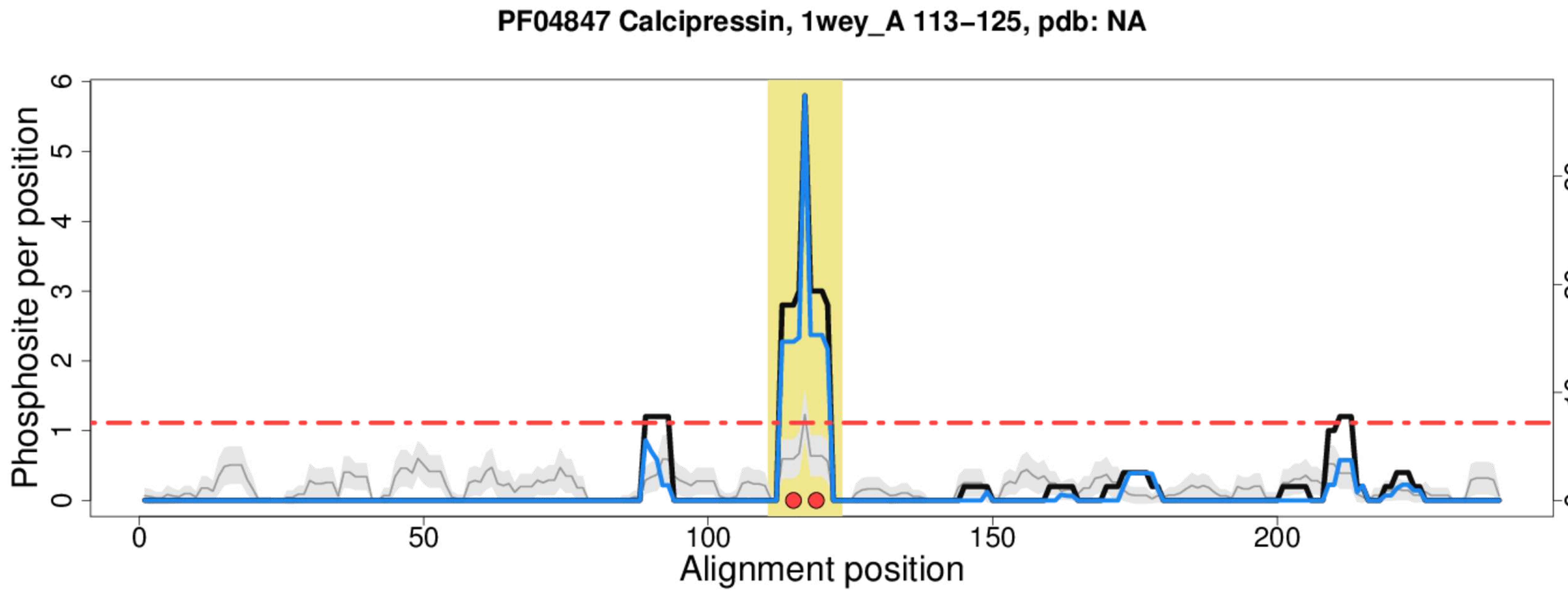

PF04847 Calcipressin, 1wey_A 113–125, pdb: NA
Phosphosite per position
–log(pvalue)
Alignment position
0
1
2
3
4
5
6
0
10
20
30
0
50
100
150
200

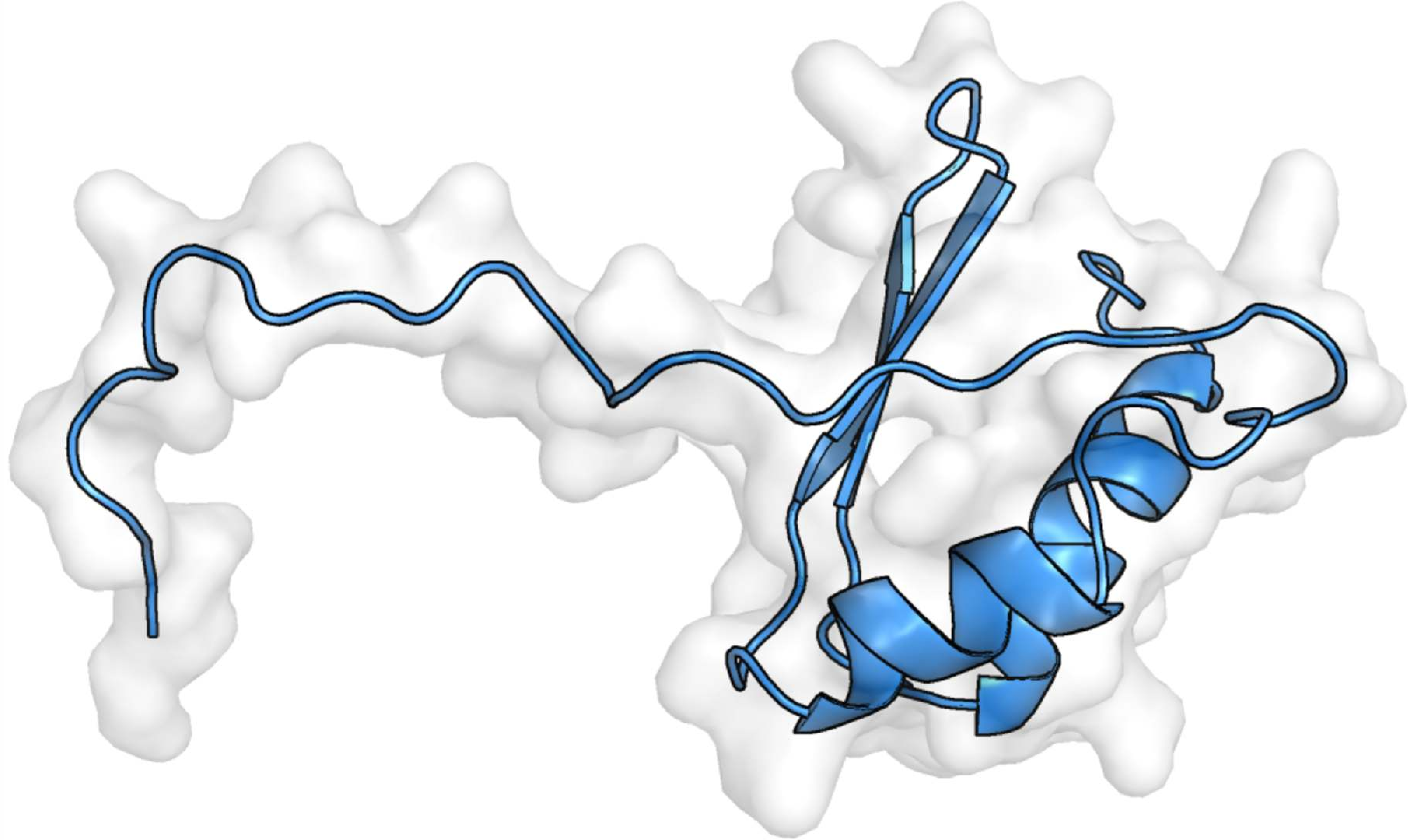

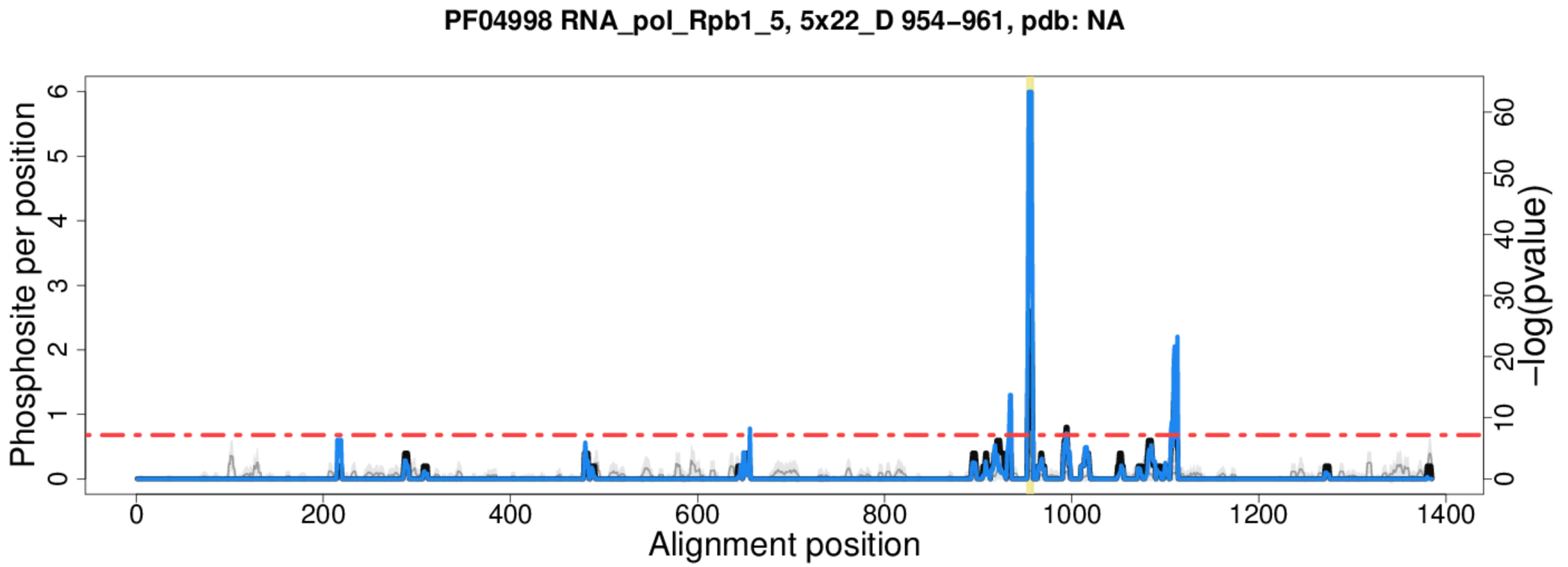
PF04998 RNA_pol_Rpb1_5, 5x22_D 954−961, pdb: NA
Phosphosite per position
0
1
2
3
4
5
6
−log(pvalue)
0
10
20
30
40
50
60
0
200
400
600
800
1000
1200
1400
Alignment position

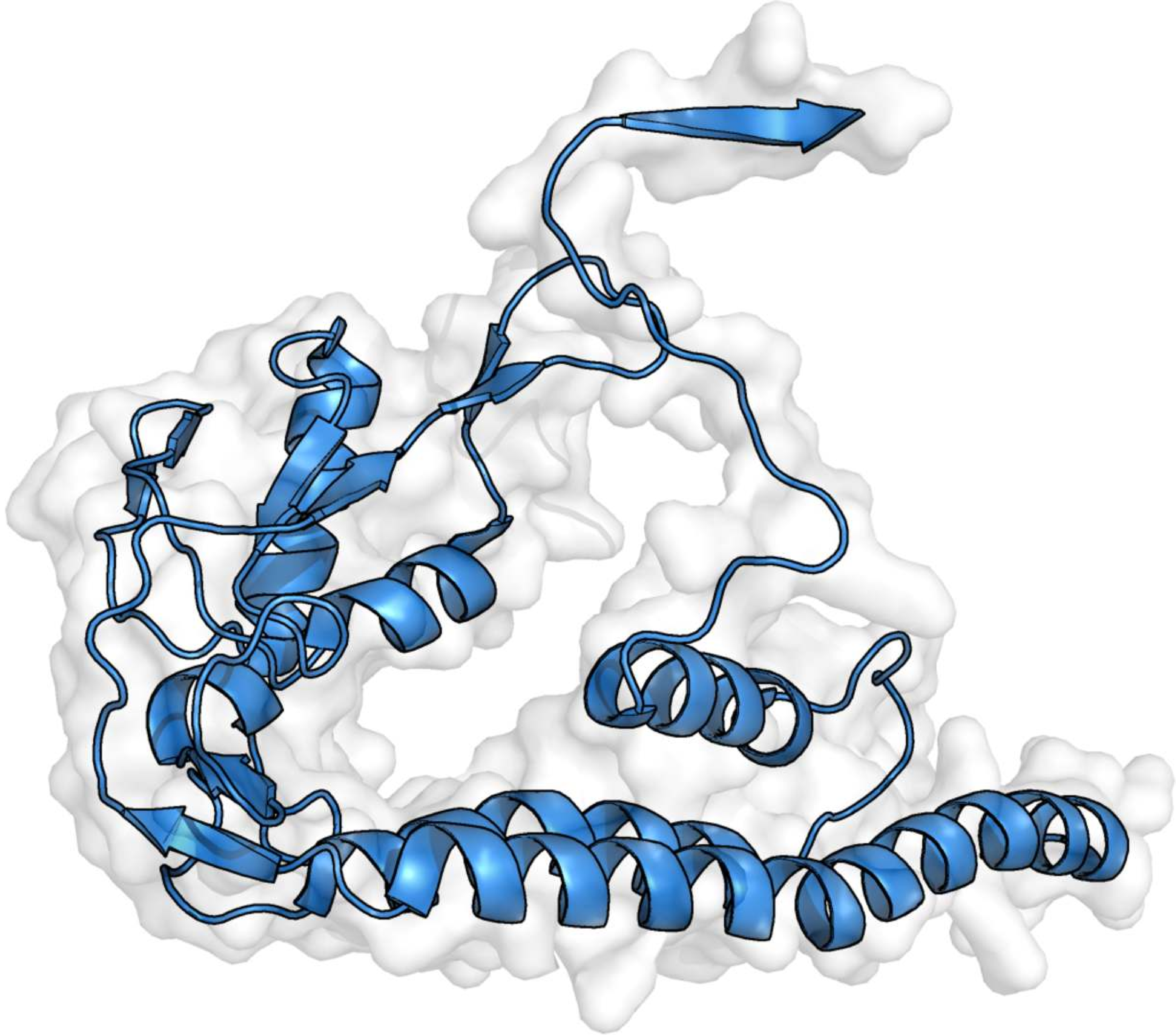

PF05470 eIF-3c_N, 3j8b_C 7-20,160-164, pdb: NA,NA

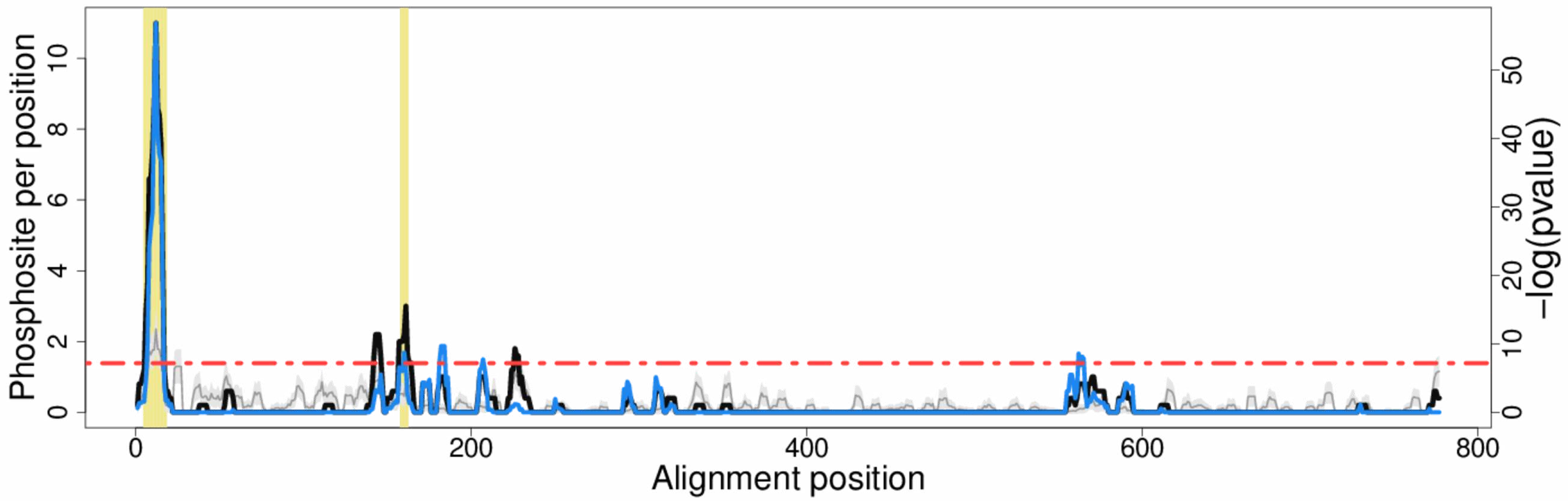

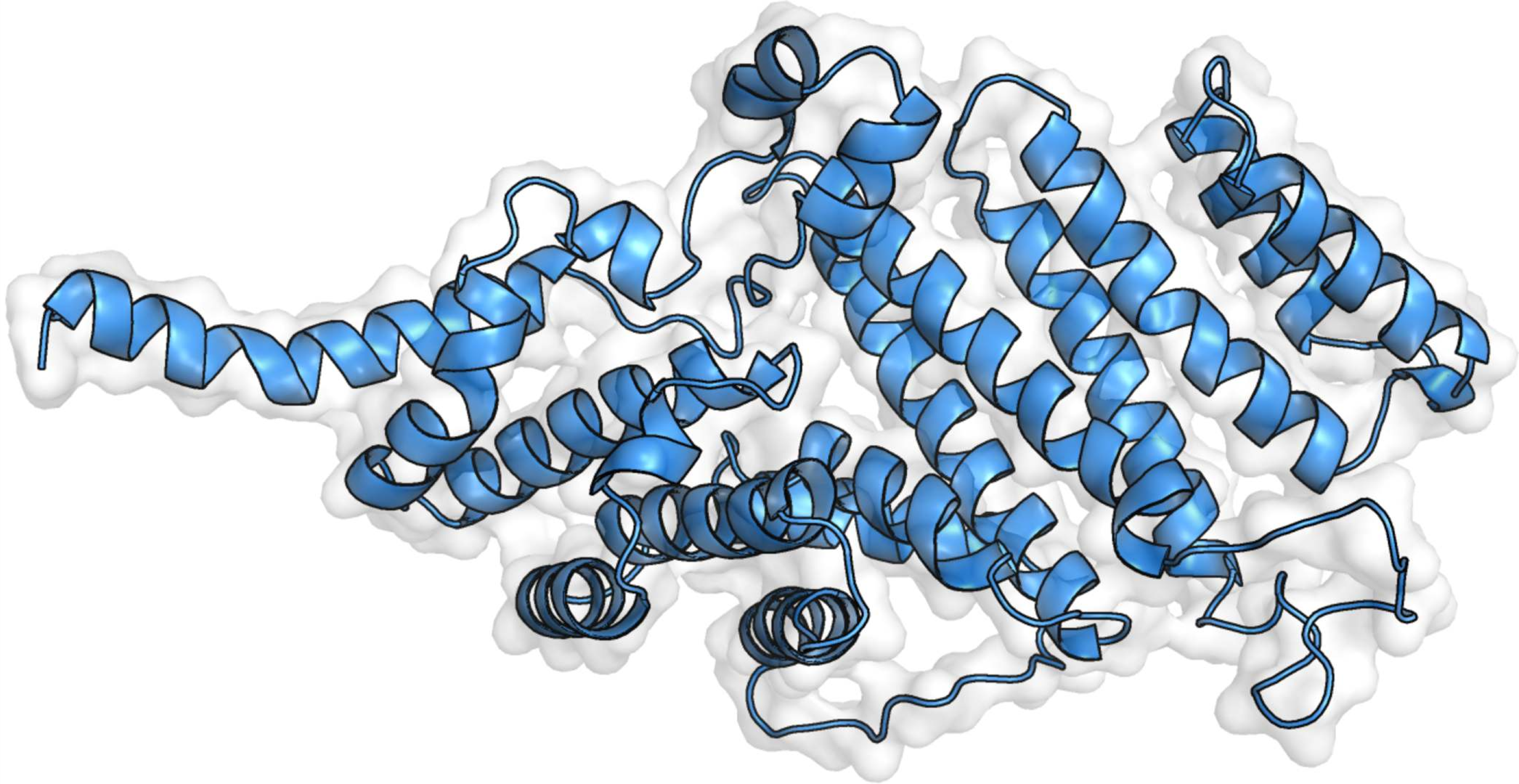

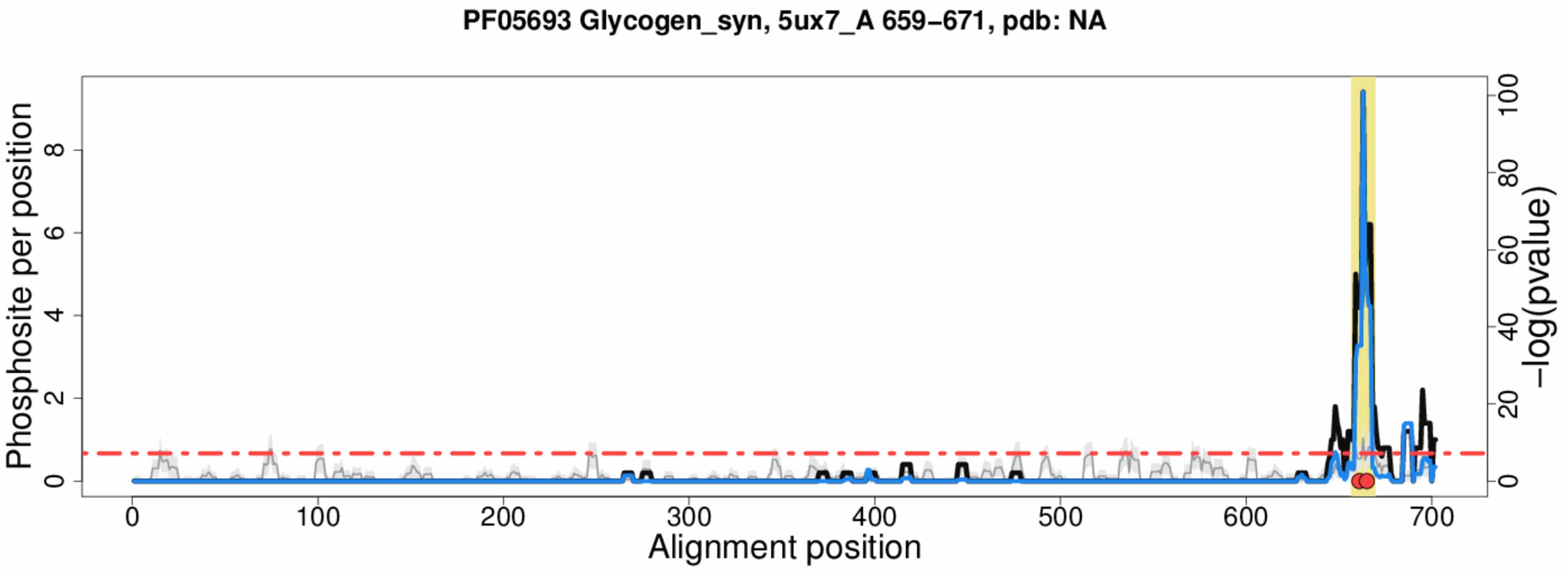
PF05693 Glycogen_syn, 5ux7_A 659−671, pdb: NA
Phosphosite per position
−log(pvalue)
Alignment position
0
2
4
6
8
0
20
40
60
80
100
0
100
200
300
400
500
600
700

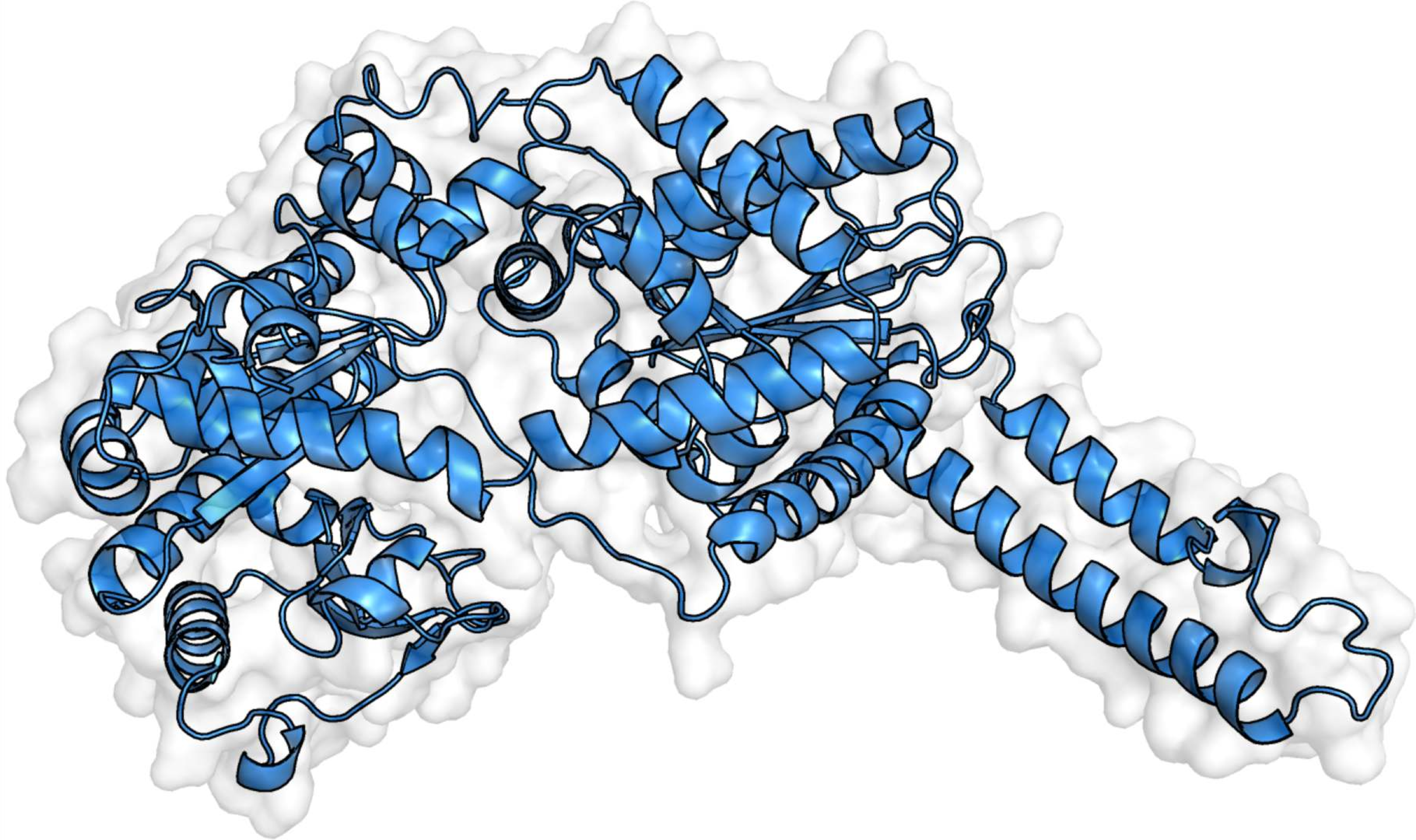

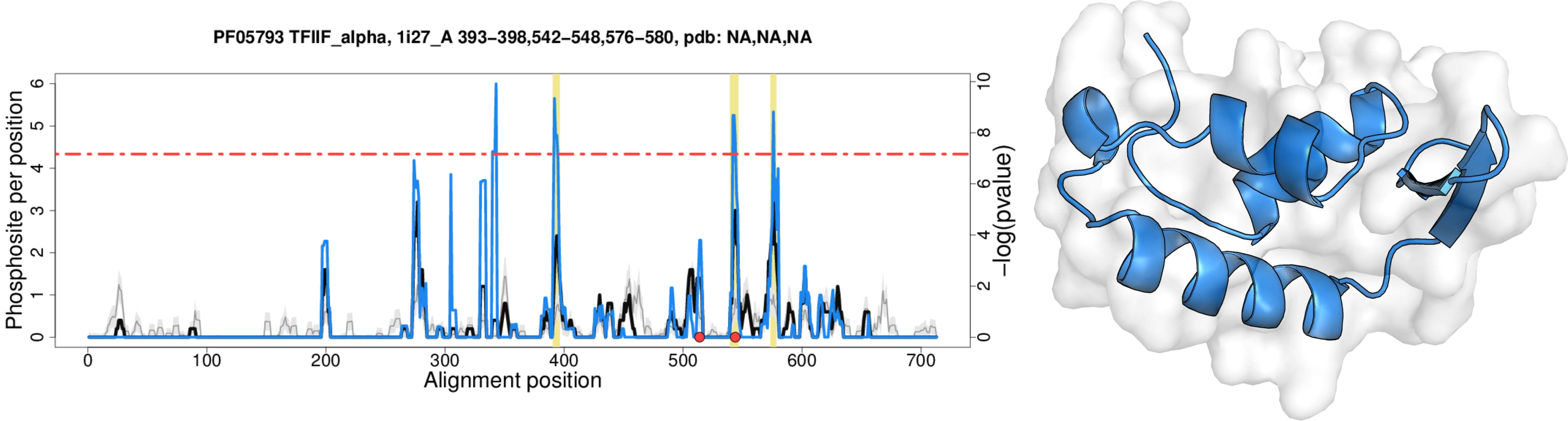

PF05793 TFIIF_alpha, 1i27_A 393−398,542−548,576−580, pdb: NA,NA,NA
Phosphosite per position
Alignment position
−log(pvalue)

PF06371 Drf_GBD, 3o4x_A 141–146, pdb: NA

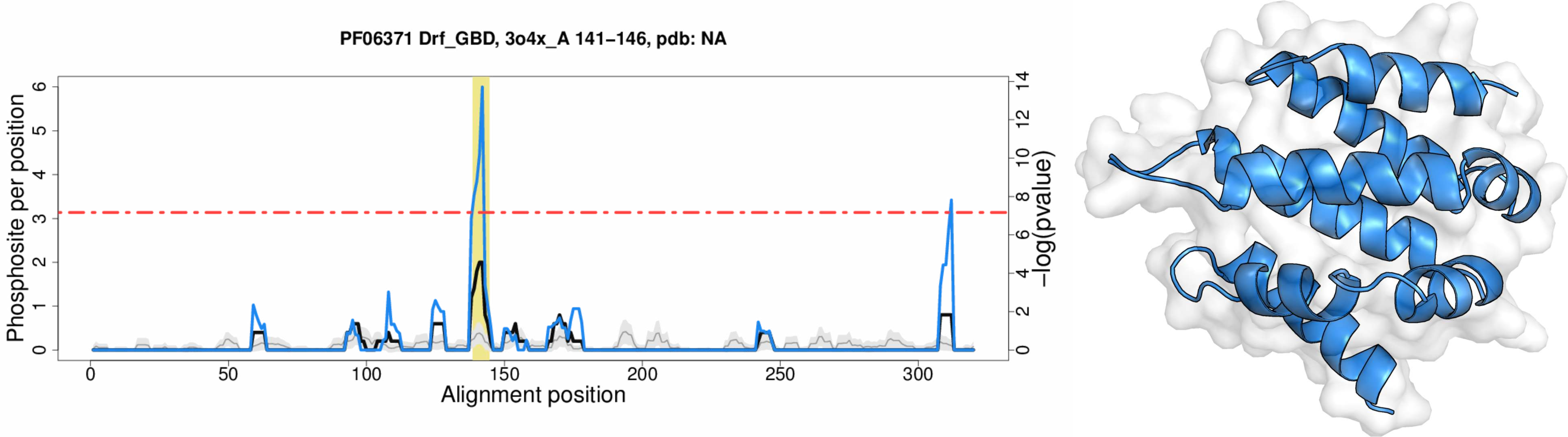

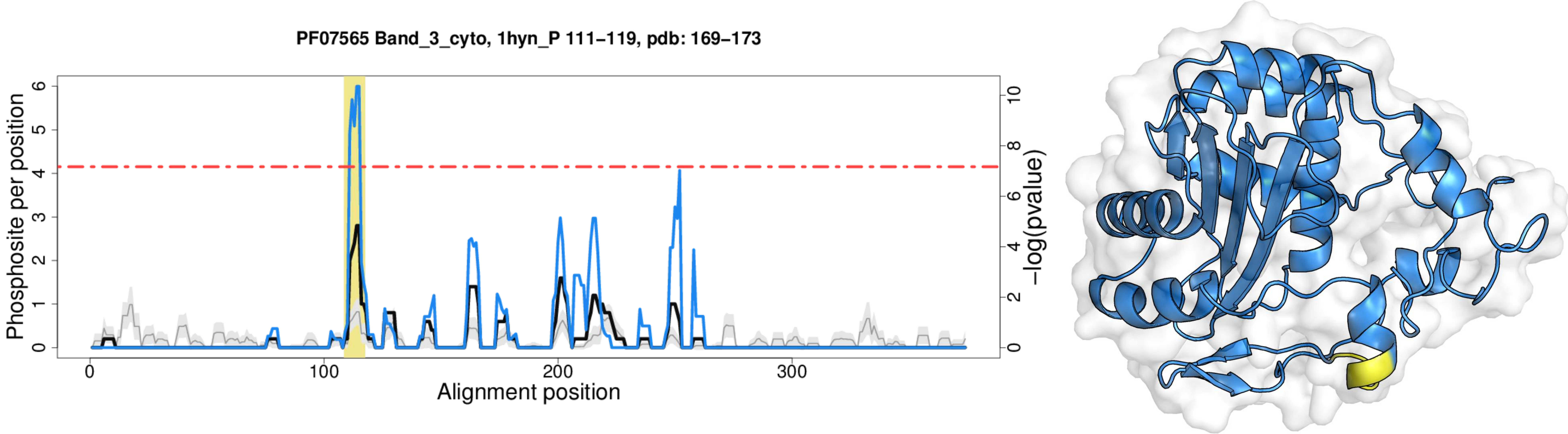
PF07565 Band_3_cyto, 1hyn_P 111–119, pdb: 169–173
Phosphosite per position
0
1
2
3
4
5
6
Alignment position
0
100
200
300
–log(pvalue)
0
2
4
6
8
10

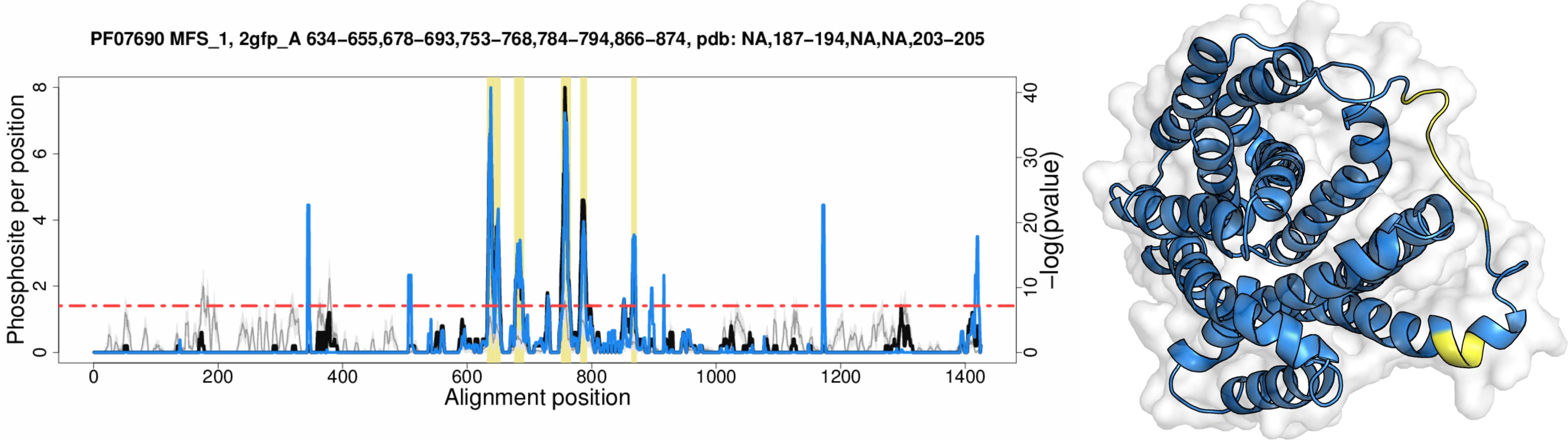
PF07690 MFS_1, 2gfp_A 634−655,678−693,753−768,784−794,866−874, pdb: NA,187−194,NA,NA,203−205
Phosphosite per position
0
2
4
6
8
Alignment position
0
200
400
600
800
1000
1200
1400
−log(pvalue)
0
10
20
30
40

PF07714 Pkinase_Tyr, 4rwk_A 9–13,347–354,648–656,665–683, pdb: 480–484,580–585,647–650,651–661

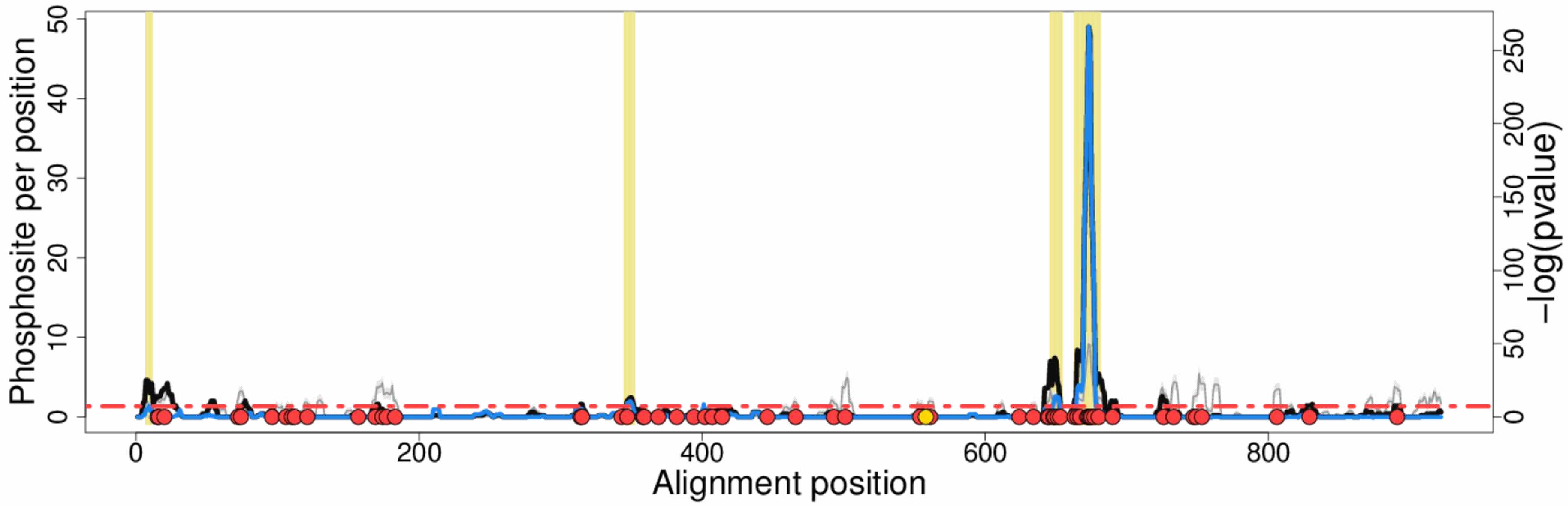

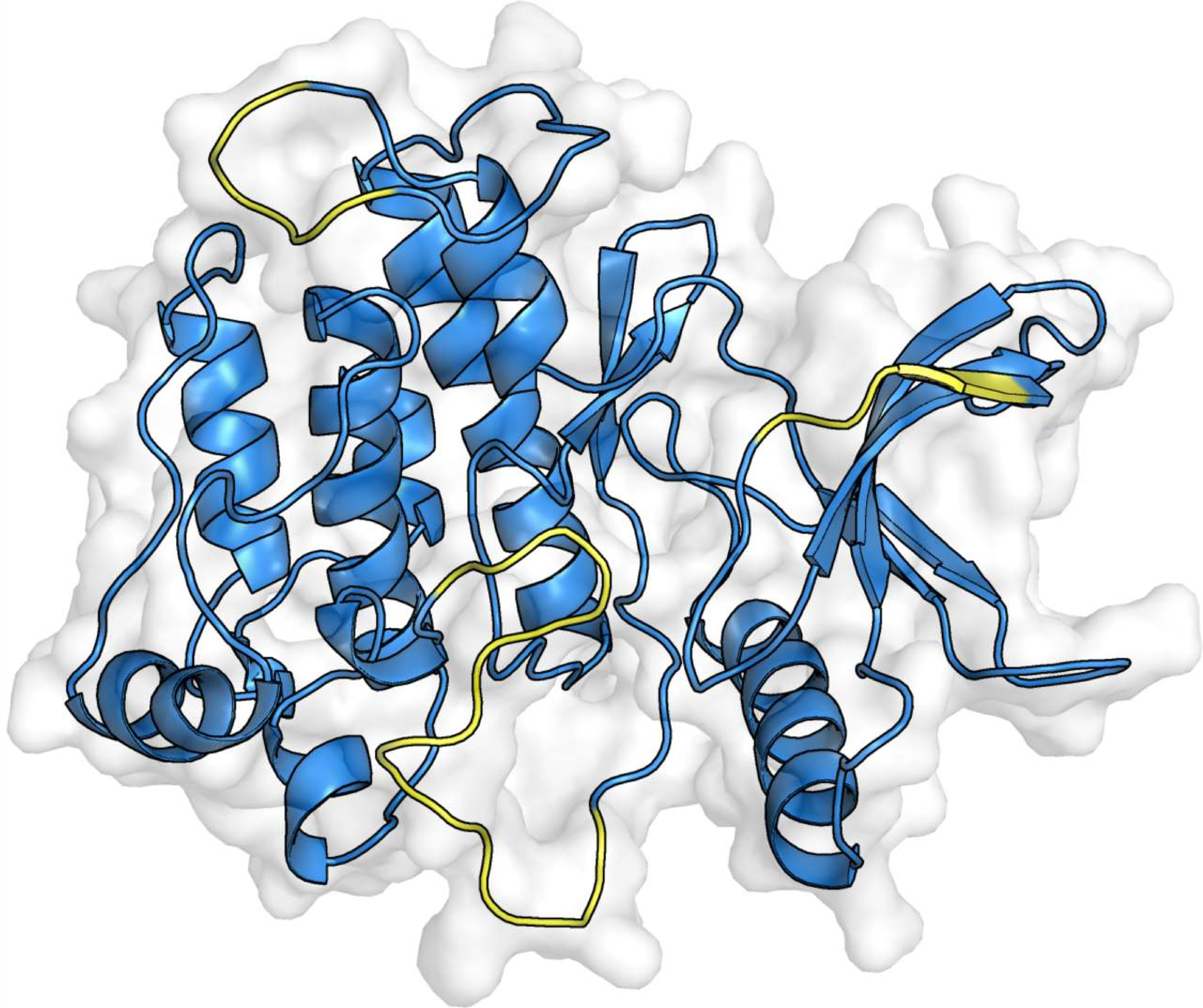

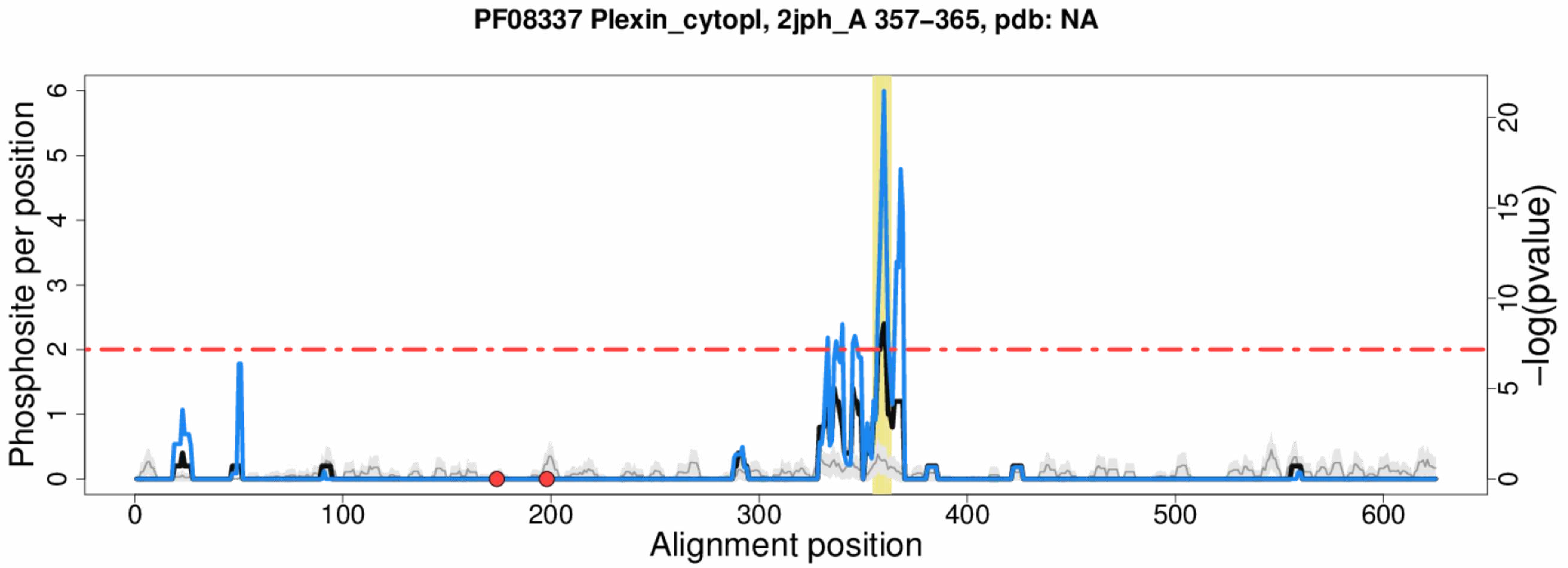
PF08337 Plexin_cytopl, 2jph_A 357−365, pdb: NA
Phosphosite per position
0
1
2
3
4
5
6
0
100
200
300
400
500
600
Alignment position
−log(pvalue)
0
5
10
15
20

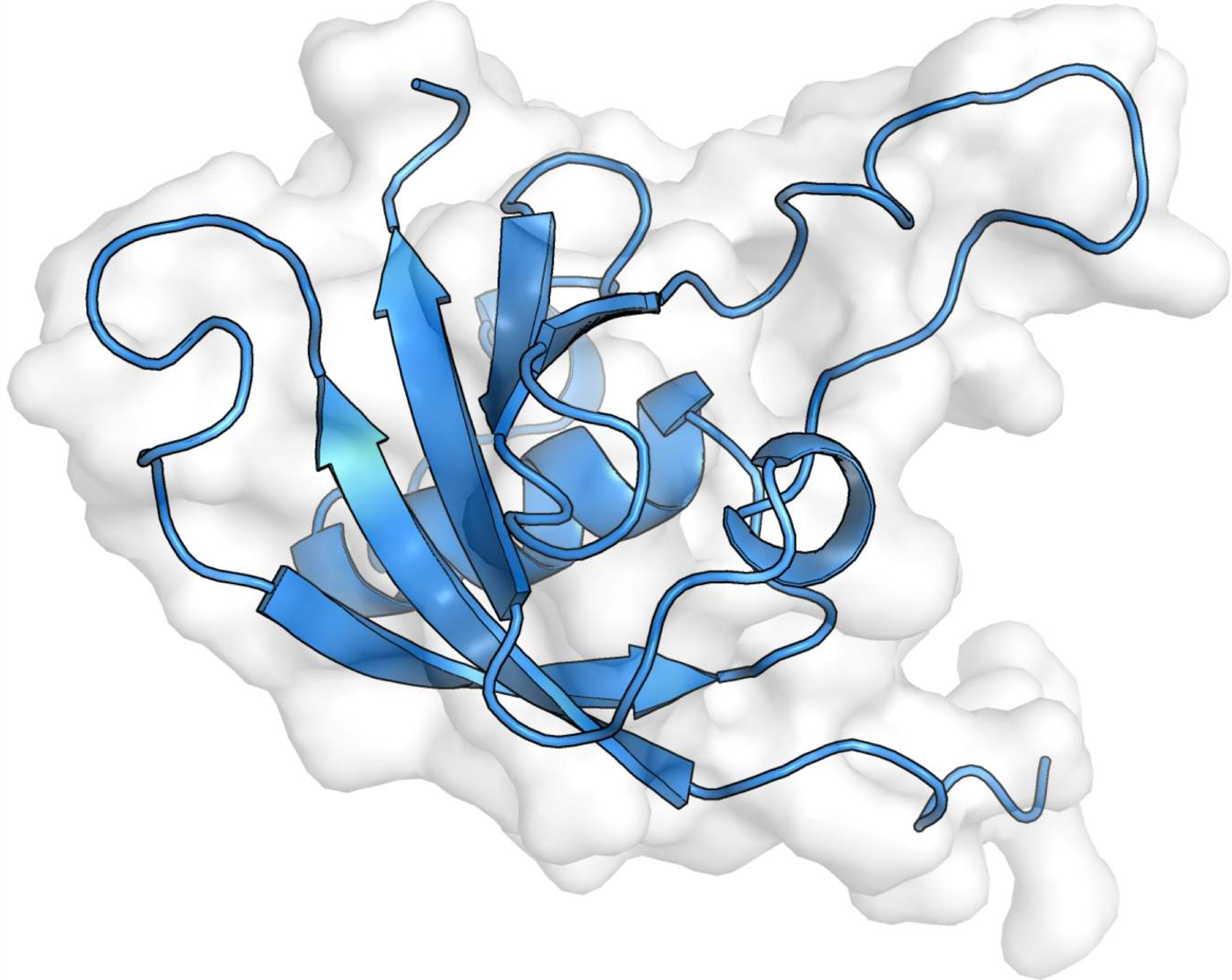

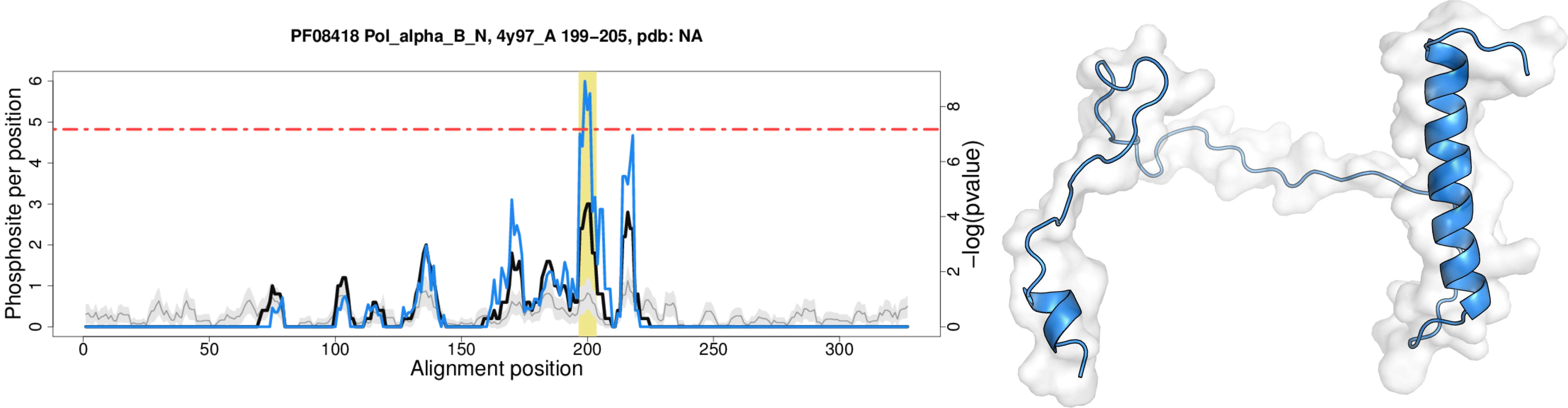
PF08418 Pol_alpha_B_N, 4y97_A 199−205, pdb: NA
Phosphosite per position
−log(pvalue)
Alignment position
0
50
100
150
200
250
300
1
2
3
4
5
6
8

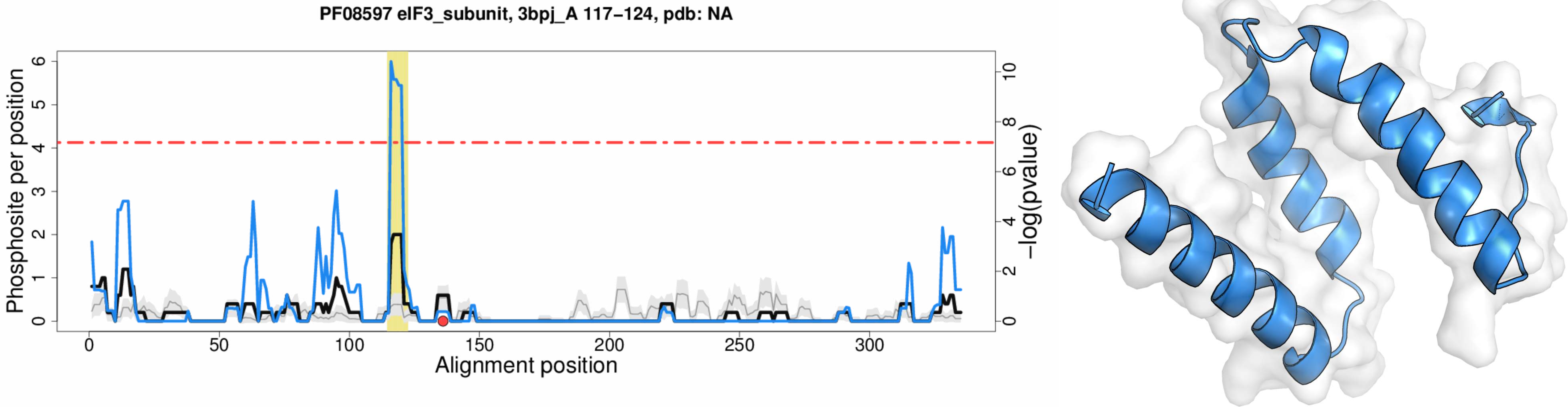
PF08597 eIF3_subunit, 3bpj_A 117–124, pdb: NA
Phosphosite per position
–log(pvalue)
Alignment position
0
1
2
3
4
5
6
0
2
4
6
8
10
0
50
100
150
200
250
300

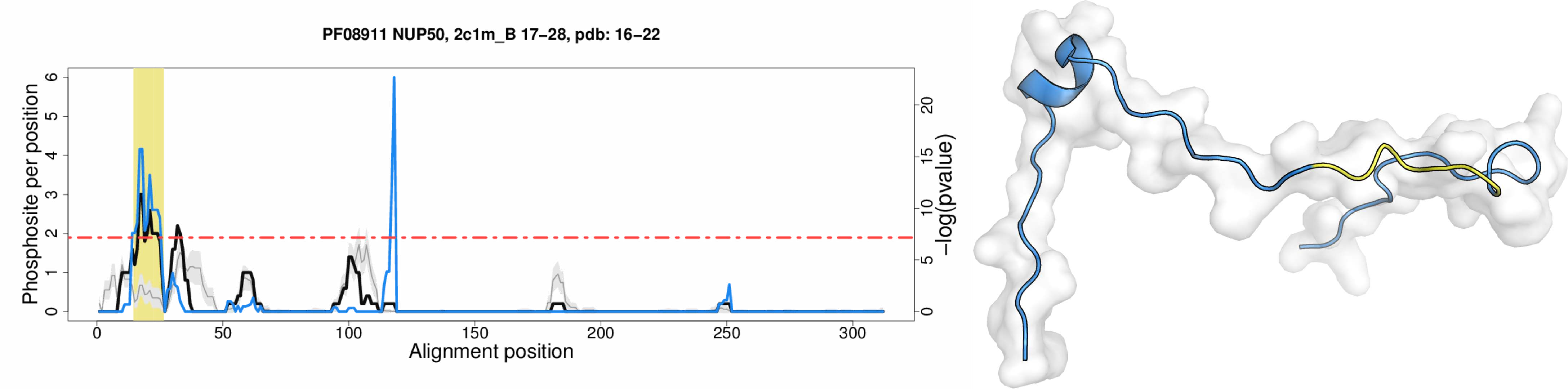

PF08911 NUP50, 2c1m_B 17−28, pdb: 16−22
Phosphosite per position
−log(pvalue)
Alignment position
0
1
2
3
4
5
6
0
5
10
15
20
50
100
150
200
250
300

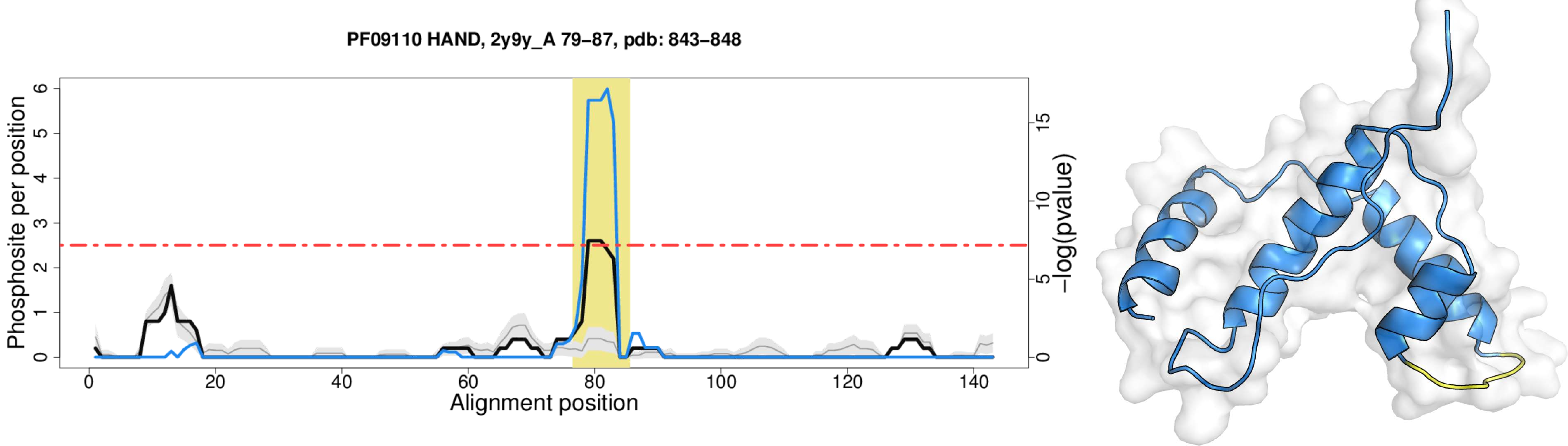
PF09110 HAND, 2y9y_A 79−87, pdb: 843−848
Phosphosite per position
−log(pvalue)
Alignment position
0
1
2
3
4
5
6
0
5
10
15
0
20
40
60
80
100
120
140

PF09359 VTC, 3g3q_A 71–79, pdb: NA

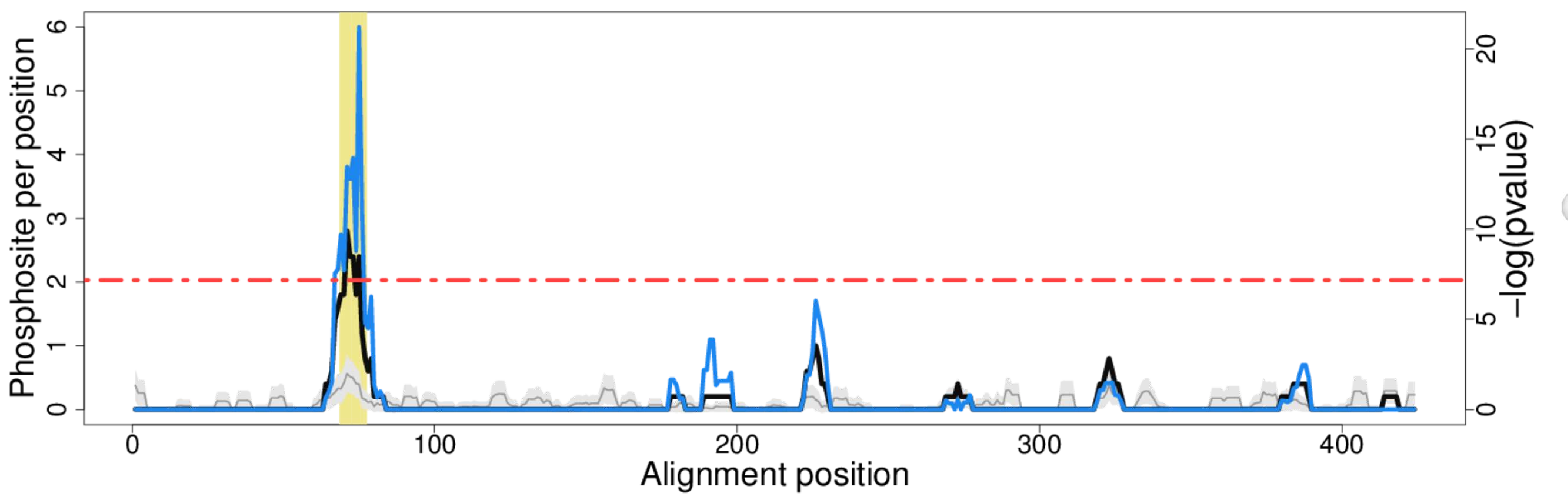

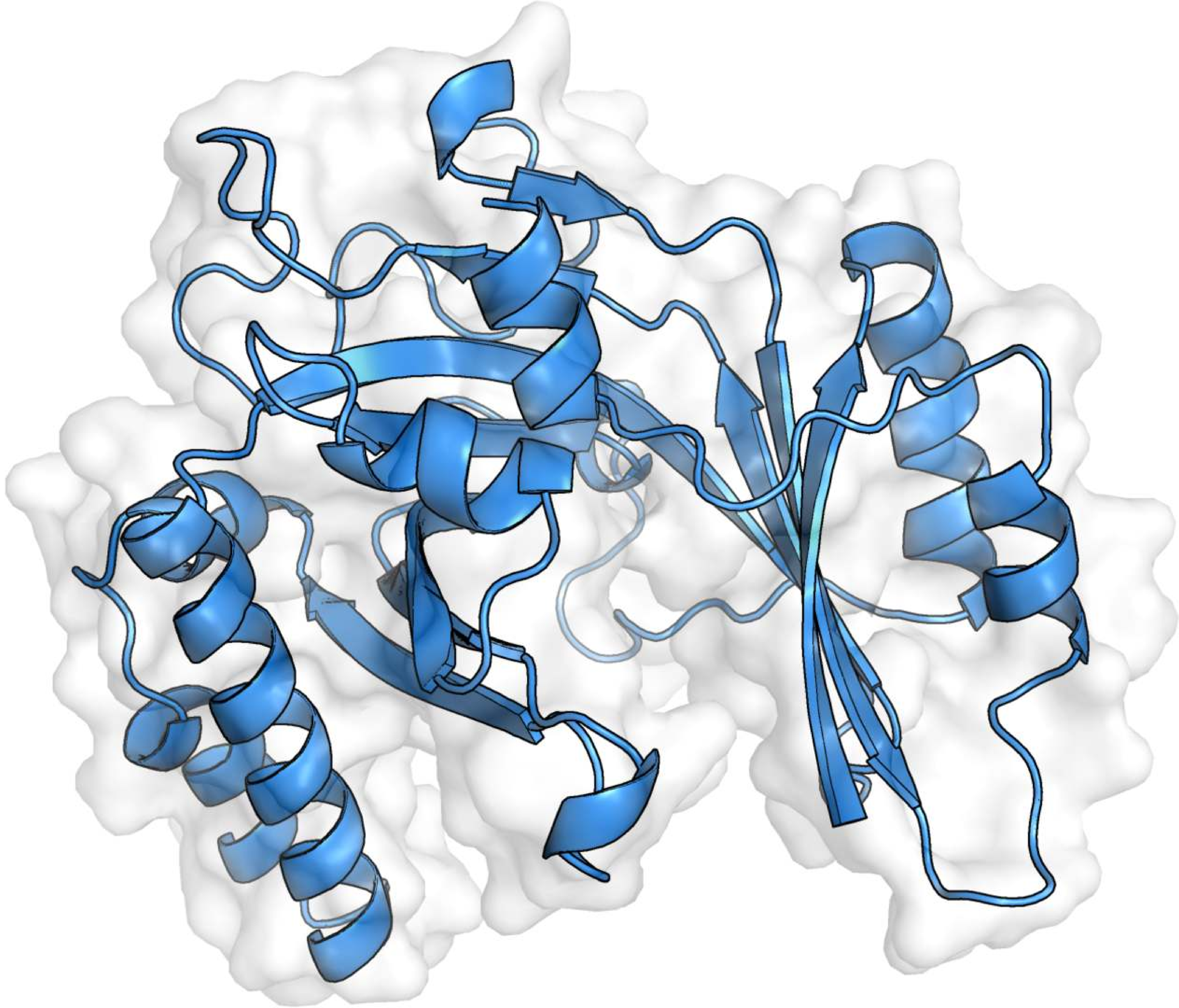

PF09380 FERM_C, 2yvc_A 91−93, pdb: 295−297

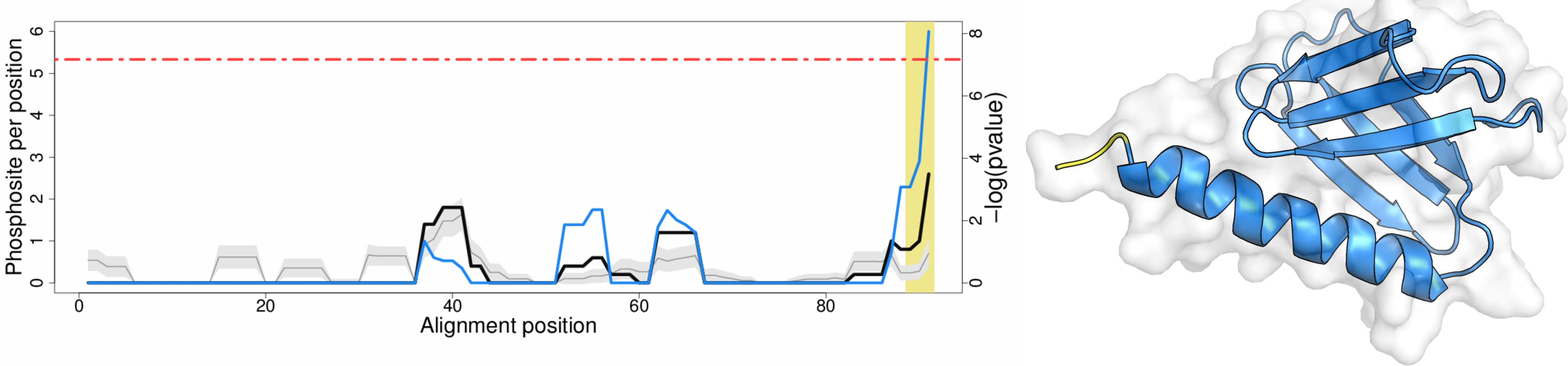

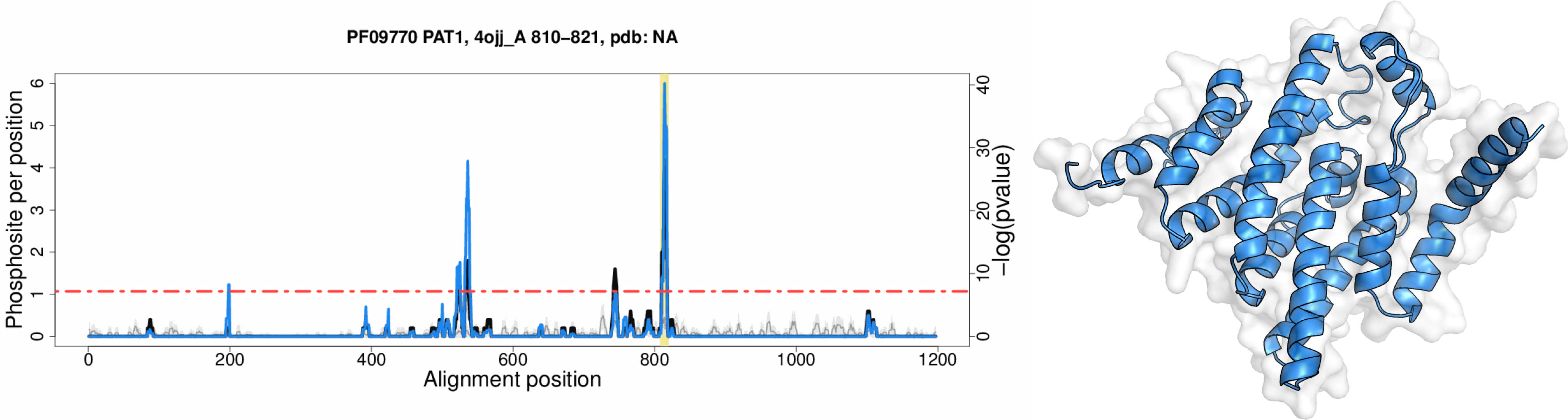

PF09770 PAT1, 4ojj_A 810–821, pdb: NA
Phosphosite per position
–log(pvalue)
Alignment position
0
1
2
3
4
5
6
0
200
400
600
800
1000
1200
0
10
20
30
40

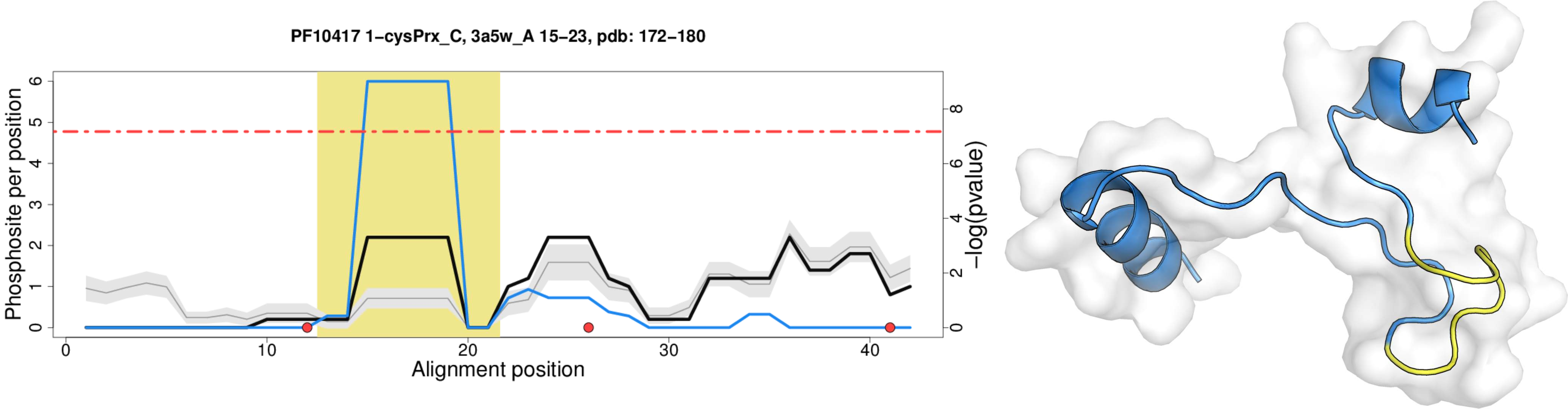
PF10417 1−cysPrx_C, 3a5w_A 15−23, pdb: 172−180
Phosphosite per position
0
1
2
3
4
5
6
0
10
20
30
40
Alignment position
−log(pvalue)
0
2
4
6
8

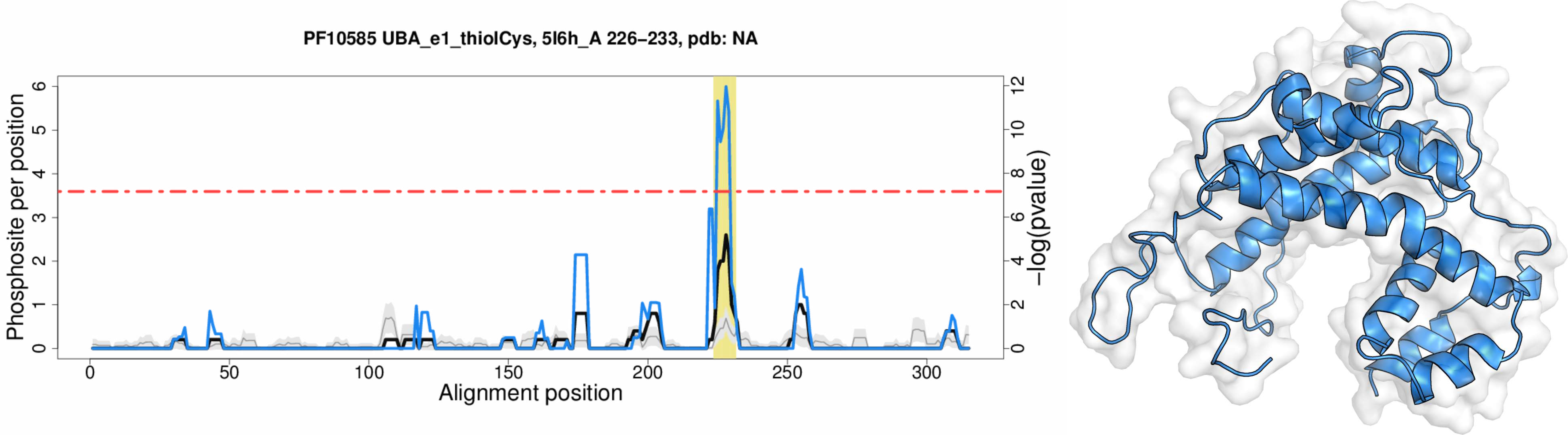
PF10585 UBA_e1_thiolCys, 5l6h_A 226–233, pdb: NA
Phosphosite per position
−log(pvalue)
Alignment position
0
50
100
150
200
250
300
0
1
2
3
4
5
6
0
2
4
6
8
10
12

PF13246 Cation_ATPase, 5xab_A 91–95, pdb: NA

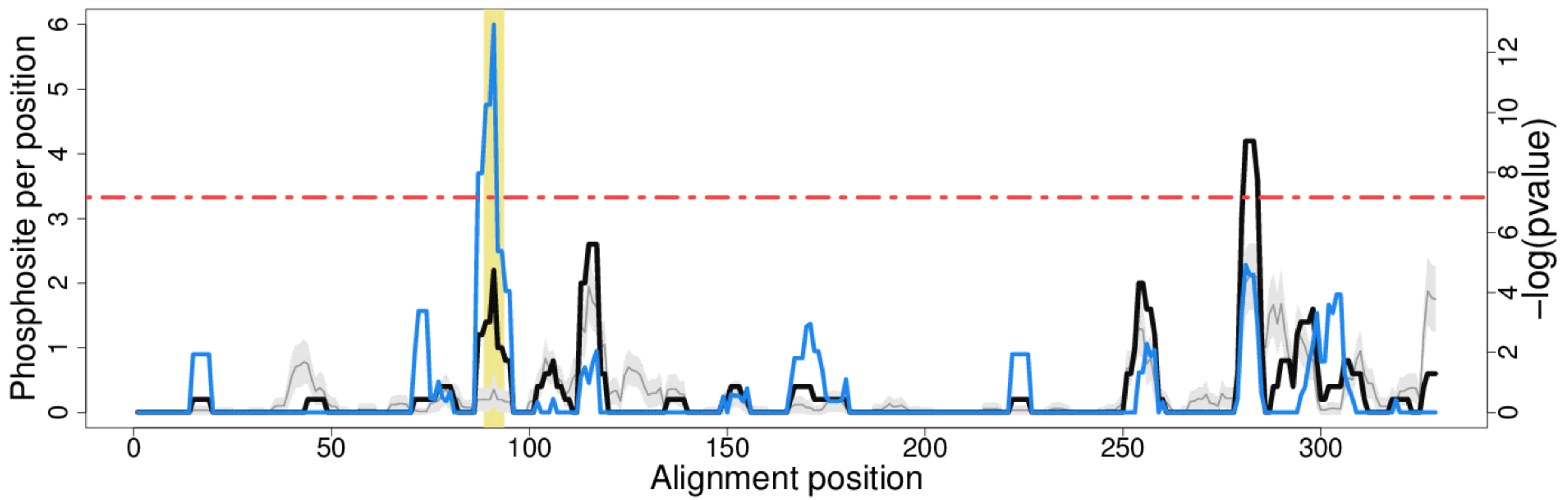

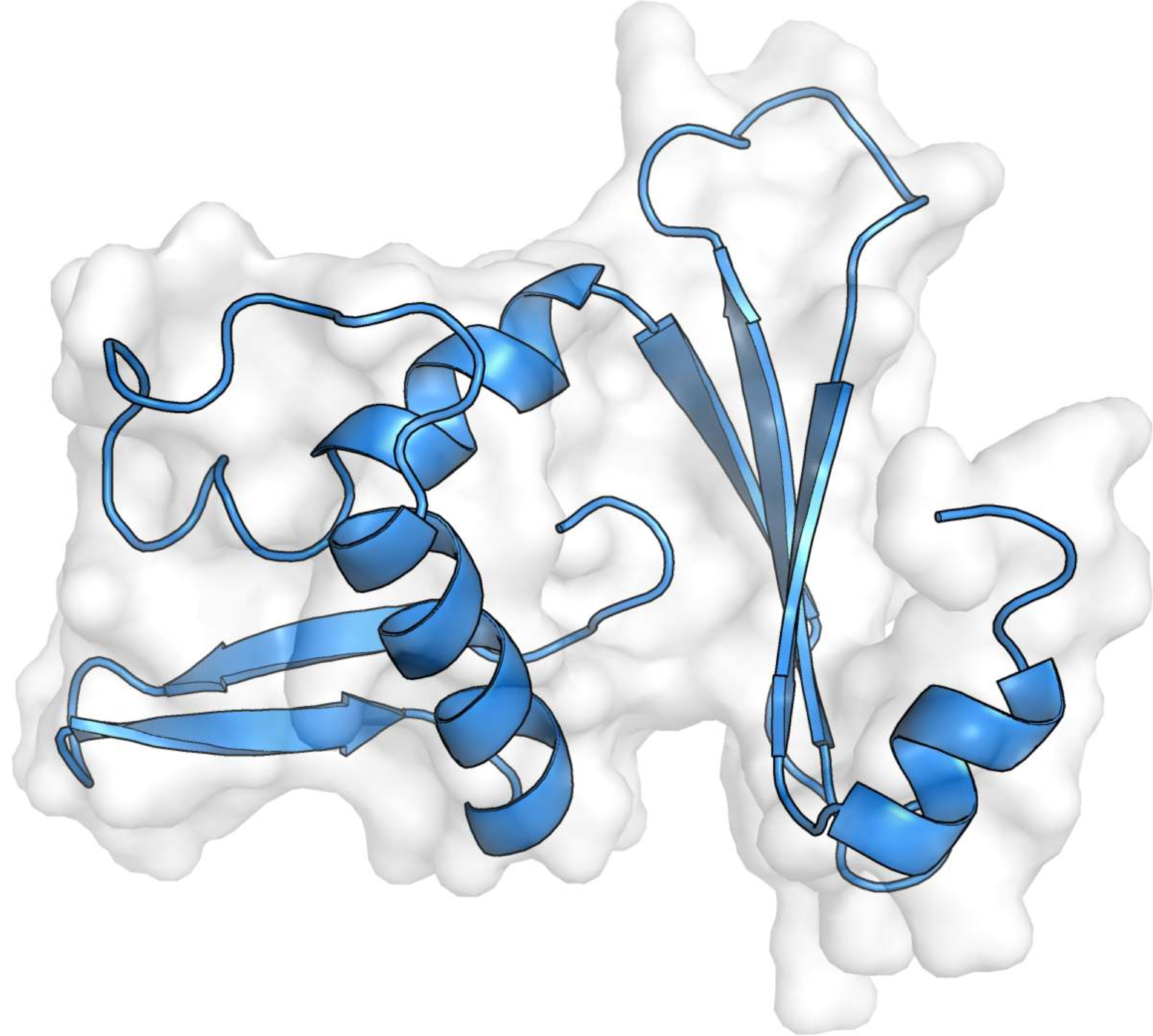

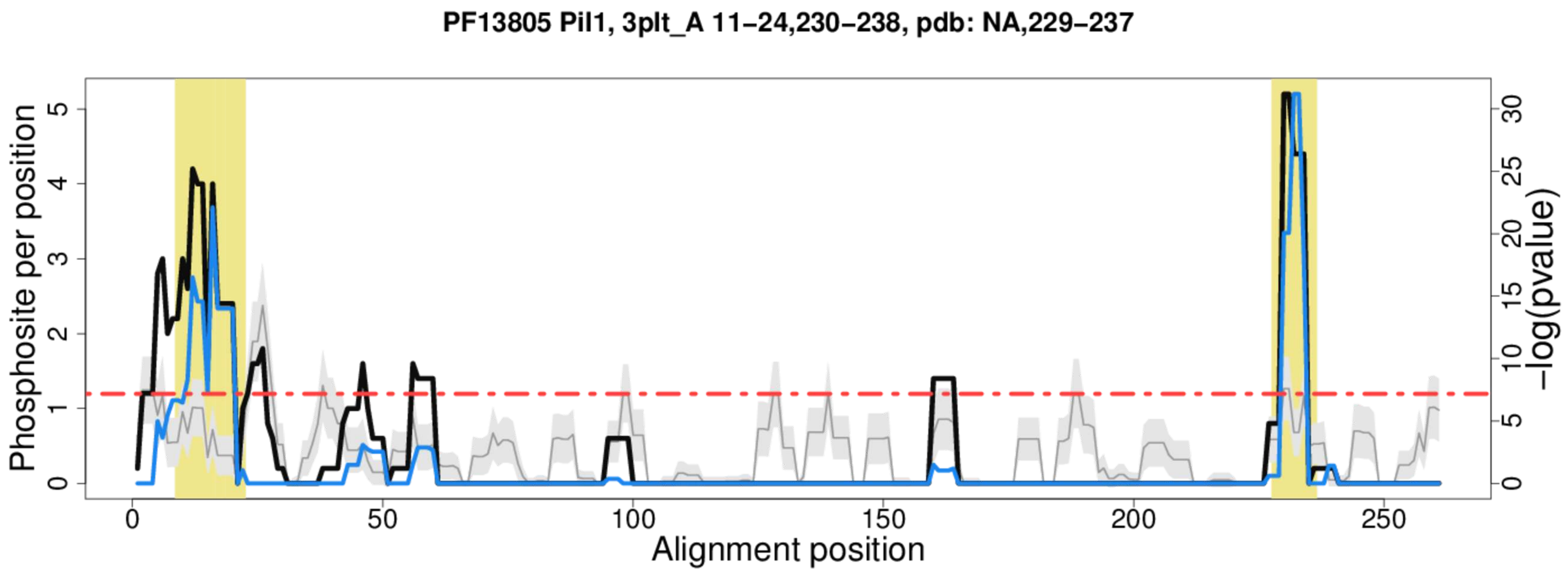
PF13805 Pil1, 3plt_A 11−24,230−238, pdb: NA,229−237
Phosphosite per position
−log(pvalue)
Alignment position
0
50
100
150
200
250
0
1
2
3
4
5
0
5
10
15
20
25
30

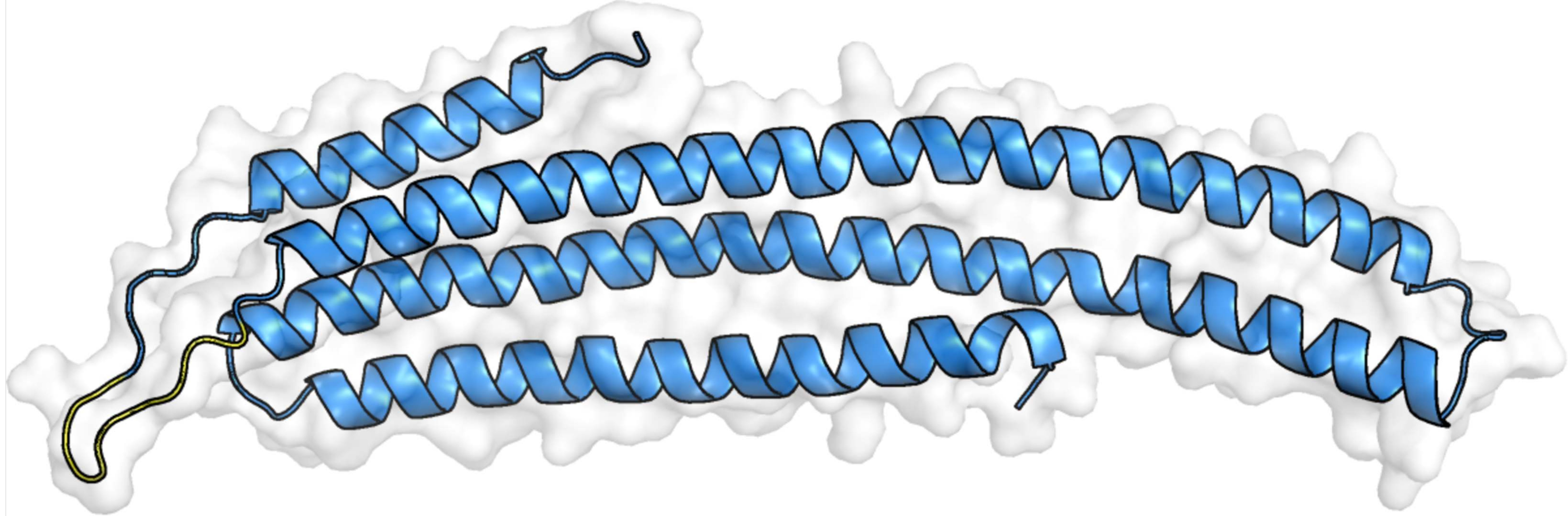

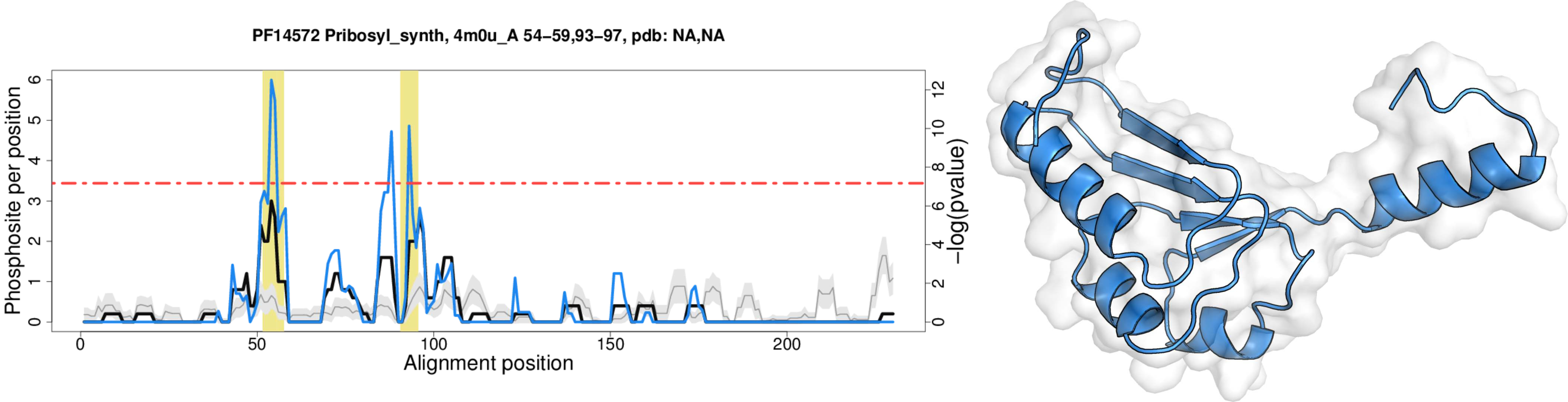
PF14572 Pribosyl_synth, 4m0u_A 54–59,93–97, pdb: NA,NA
Phosphosite per position
0
1
2
3
4
5
6
−log(pvalue)
0
2
4
6
8
10
12
Alignment position
0
50
100
150
200

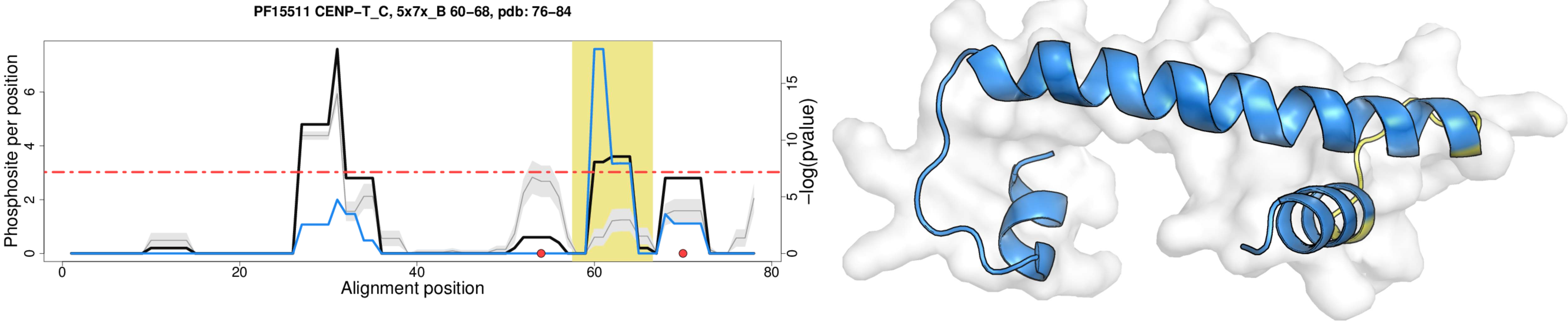

PF15511 CENP−T_C, 5x7x_B 60−68, pdb: 76−84
Phosphosite per position
−log(pvalue)
Alignment position

PF16275 SF1-HH, 2m09_A 79-90, pdb: 78-87

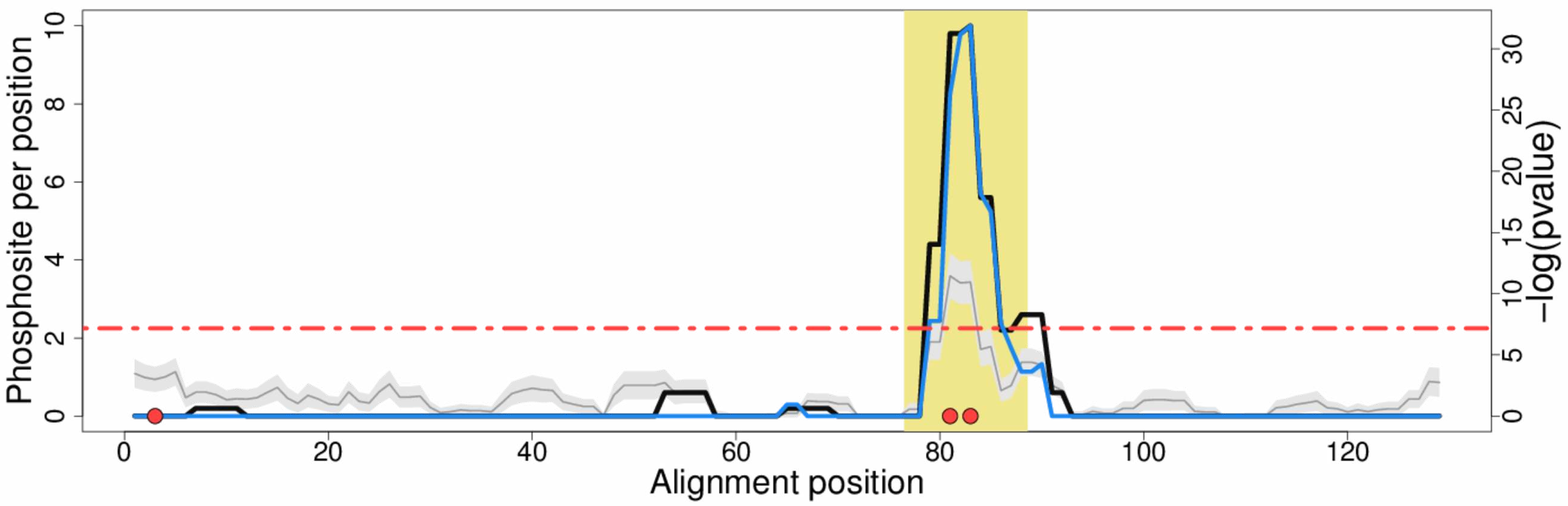

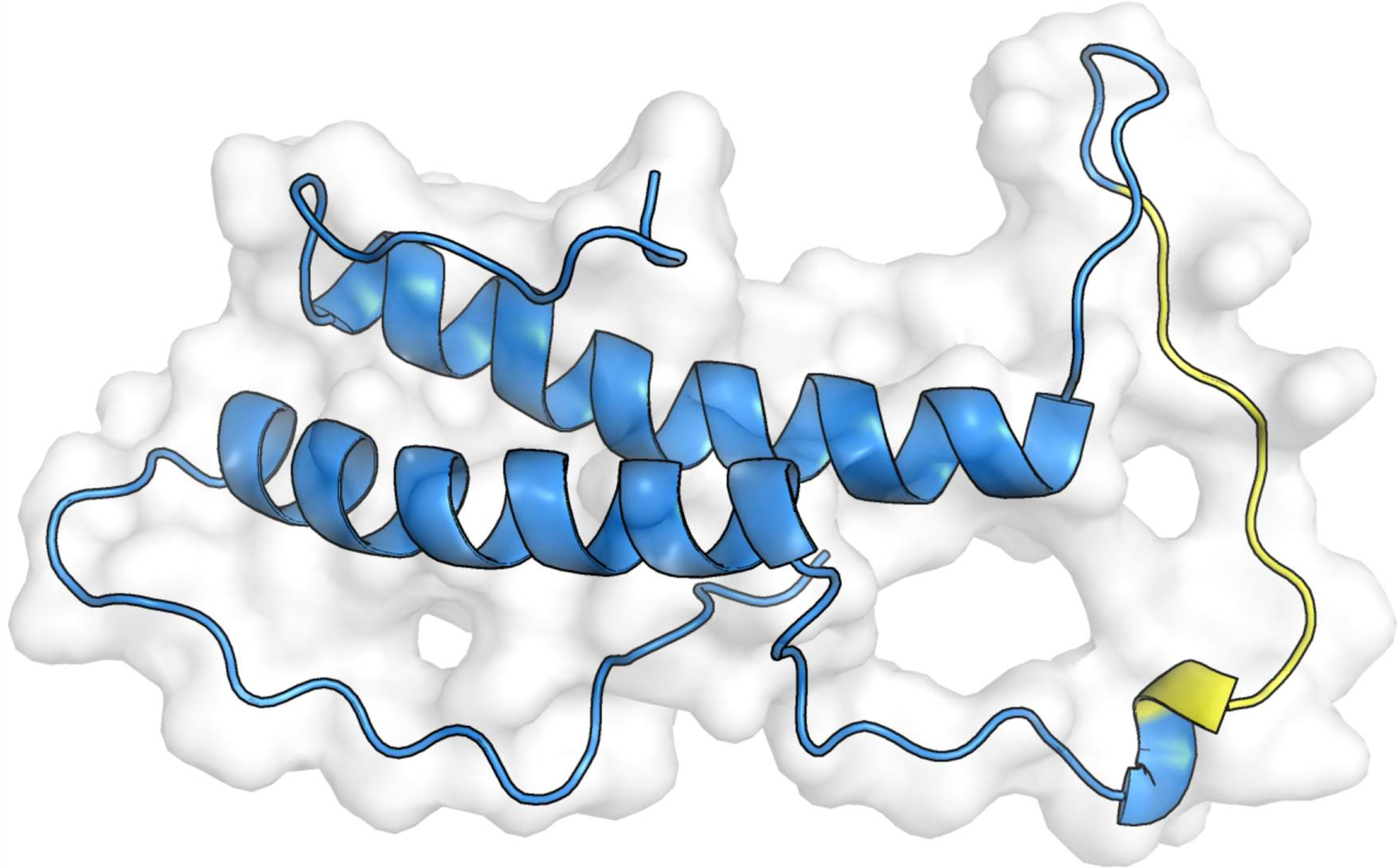

Supplement: Supplementary file 5 — Supplementary Data 2 [file 41467_2019_9952_MOESM5_ESM.pdf]
