# Supplementary material for: Conserved phosphorylation hotspots in eukaryotic protein domain families
Source: Nat Commun. 2019 Apr 29;10:1977. doi: 10.1038/s41467-019-09952-x (PMC6488607; doi:10.1038/s41467-019-09952-x)
Supplement: Supplementary file 7 — Source Data [file 41467_2019_9952_MOESM7_ESM.zip › sourcedata_supp2b3c.pdf]

Supplementary Figure 2B upper panel

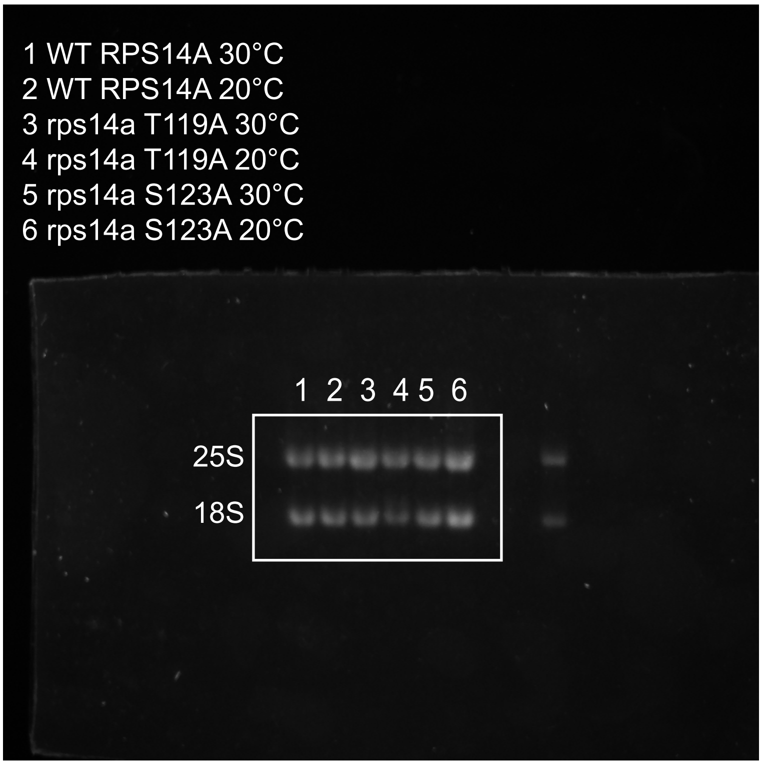

Supplementary Figure 3C upper panel

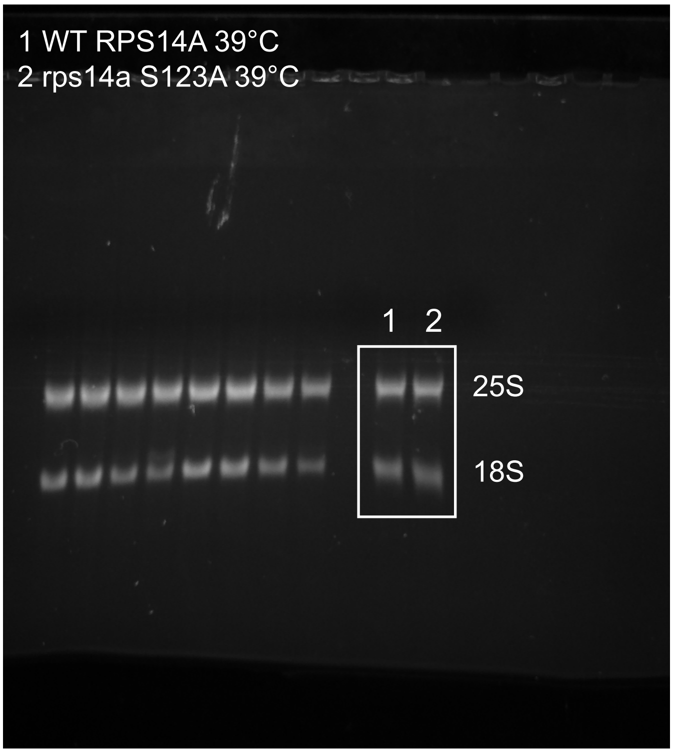

Supplementary Figure 2B lower panel

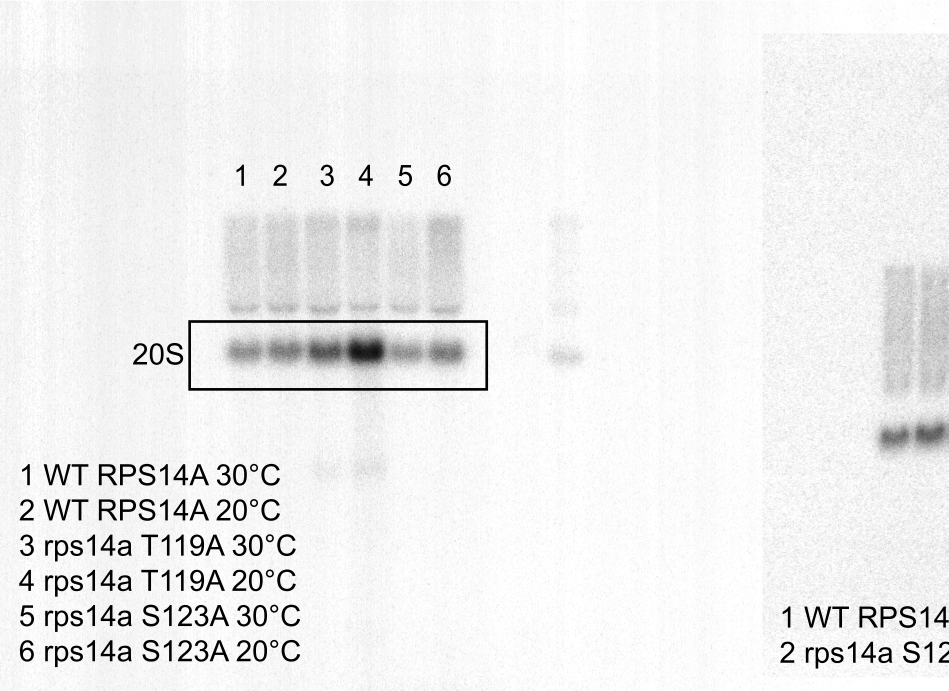

Supplementary Figure 3C lower panel

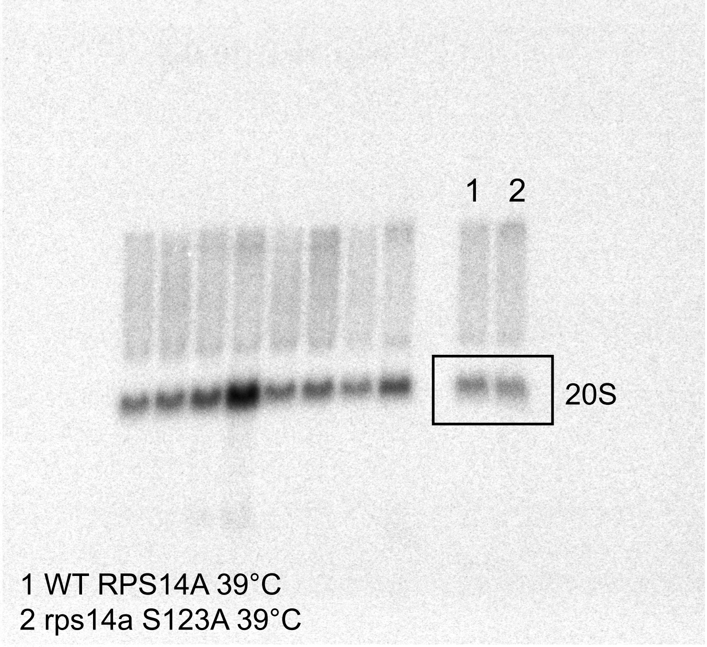

To detect 20S - probe complementary to ITS1 named 004a (mod. From Oeffinger et al 2009) was used, synthesized by Microsynth.
Probe was radiactively labelled with P^{32} isotop on its 5’end using gamma P^{32}ATP
